# Cafestol-derivatives as potential FXR agonists and CYP7A1 inhibitors and their impact on hypercholesterolemia: an in silico study

**DOI:** 10.1038/s41598-026-37519-6

**Published:** 2026-02-03

**Authors:** Maria Alice Esteves da Silva, Priscila Goes Camargo, Camilo Henrique da Silva Lima, Carlos Rangel Rodrigues, Magaly Girão Albuquerque, Claudia Moraes de Rezende

**Affiliations:** 1 Instituto de Química, Departamento de Química Orgânica, Universidade Federal do Rio de Janeiro, Programa de Pós-Graduação em Ciência de Alimentos (PPGCAL), Laboratório de Análise de Aromas CEP 21941-909, Cidade Universitária, Rio de Janeiro, RJ Brazil; 2 Instituto de Química, Departamento de Química Orgânica, Laboratório de Modelagem Molecular (LabMMol), Universidade Federal do Rio de Janeiro, Cidade Universitária, Rio de Janeiro, CEP 21941-909 RJ Brazil; 3 Departamento de Fármacos e Medicamentos, Faculdade de Farmácia, Laboratório de Modelagem Molecular e QSAR (ModMolQSAR), Universidade Federal do Rio de Janeiro, Cidade Universitária, Rio de Janeiro, CEP 21941-170 RJ Brazil

**Keywords:** Coffee diterpenes, Unfiltered coffee, Molecular modelling, Cholesterol-raising, *Ent*-kaurane, Roasted coffee, Biochemistry, Chemical biology, Chemistry, Computational biology and bioinformatics, Drug discovery

## Abstract

Cafestol is a hypercholesterolemic *ent*-kaurane diterpene found in unfiltered coffee, a regular component of the human diet. This diterpene is reported to modulate macromolecules involved in cholesterol metabolism, such as the farnesoid X receptor (FXR) and the cytochrome P450 7A1 enzyme (CYP7A1) in both in vitro and in vivo models. However, there are no studies on the hypercholesterolemic potential of cafestol roasting derivatives and phase I metabolites of cafestol, which share the same *ent*-kaurane scaffolds. In the present work, we compared binding modes of cafestol roasting derivatives and phase I metabolites of cafestol identified in a zebrafish model with two human protein-targets, FXR and CYP7A1, using molecular docking and molecular dynamics simulations (MDS) guided by cavity detection studies. We also performed a pharmacophore map focusing on the steroidal skeleton of co-crystallized ligands of these two protein targets and compared it with the *ent*-kaurane nucleus, along with an in silico ADME analysis of cafestol roasting derivatives to predict their physicochemical and pharmacokinetic properties. Based on their structural similarity to cafestol, this study preliminarily supports that these compounds may contribute to increased serum cholesterol, through interaction with these targets, thereby intensifying the cardiovascular risk associated with the consumption of unfiltered coffee beverages.

**Supplementary Information:**

The online version contains supplementary material available at 10.1038/s41598-026-37519-6.

## Introduction

**Fig. 1:**
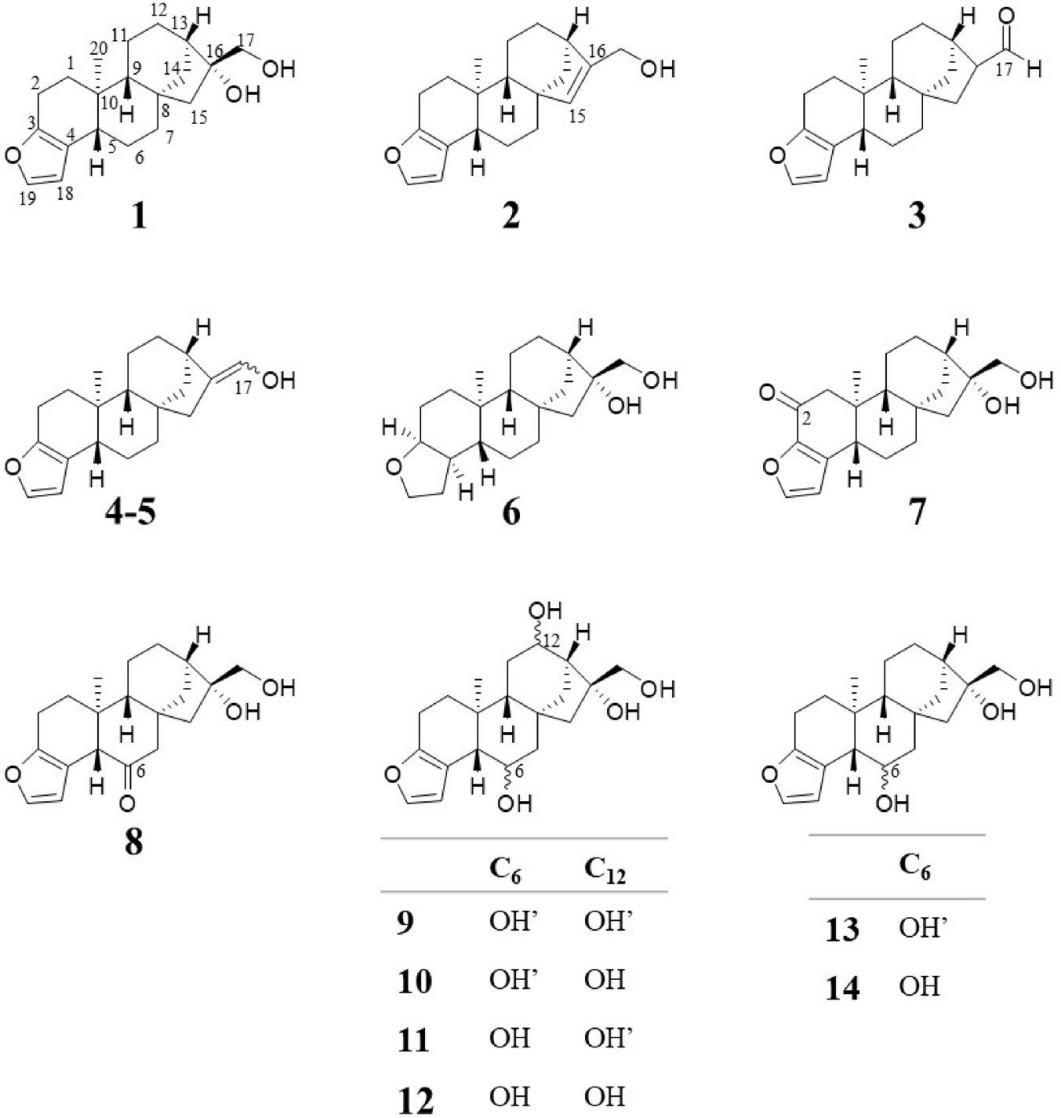
Chemical structures of cafestol (**1**); cafestol roasting products (**2**–**5**), 15,16-dehydrocafestol (**2**), cafestal (**3**), and (*E* / *Z*) 16,17-dehydrocafestol (**4** and **5**); hydrogenated cafestol (**6**); and cafestol phase I metabolites (**7**–**14**) from a zebrafish model. OH’ represents the hydroxyl group oriented towards the front of the plane.

Cafestol (**1**) (Fig. 1) is an *ent*-kaurane furan-diterpenoid present in the lipid fraction of coffee beans and one of the main bioactive compounds in coffee beverages. Despite the potentially beneficial effects, such as antitumor, antioxidant, and anti-diabetes, reported in pre-clinical studies, cafestol has deleterious effects on lipid metabolism in humans^1^. Excessive consumption of unfiltered coffee drinks may lead to cholesterol-raising^2^. Coffee brewing method is a determinant factor for this deleterious effect, since beverages such as espresso, mocha, French press, and boiled coffee contain higher concentrations of cafestol and other lipids than filtered coffee. Cafestol is the main compound in coffee beans that shows a potent hypercholesterolemic activity^3^. The cholesterol-raising effect of cafestol was established in the 1990s after epidemiological studies in Norway demonstrated an increase in serum cholesterol associated with the consumption of boiled coffee beverages^4^. A daily intake of 10 mg of cafestol over a period of four weeks results in an increase of 5 mg dL^− 1^ in serum cholesterol levels^3^.

Although numerous positive biological activities have been reported for cafestol,^1^ its impact on increasing serum cholesterol was the first to progress to clinical studies and is considered the most notable adverse effect observed so far^5,6^. Different proposals suggested the mechanism of cafestol on cholesterol metabolism in literature^2,7^. The inhibition of cholesterol biotransformation into bile acids and reduction of this steroid excretion due to cafestol’s farnesoid X receptor (FXR) agonist activity and a putative direct cytochrome P450 7A1 enzyme (CYP7A1) inhibition activity, respectively, may represent a comprehensive explanation^8,9^. In the context of animal models for studies of cafestol effects on cholesterol metabolism, only the validated apolipoprotein E*3-Leiden transgenic mice model has a human-like hypercholesterolemic response^10,11^.

FXR, also known as bile acid receptor, is a member of the nuclear receptor superfamily expressed in the liver and intestine, acting in the regulation of bile acid metabolism and lipid homeostasis^12,13^. When activated by bile acids (endogenous agonists), the signaling cascade results in the activation of the orphan nuclear receptor small heterodimer partner (SHP), a regulator of gene expression of bile acid metabolism^14^. Cholesterol-7-α-hydroxylase (CYP7A1) is a cytochrome P450 (CYP) monooxygenase enzyme involved in the metabolism of endogenous cholesterol and its oxygenated derivatives (oxysterols)^15^. Modulated by FXR, this enzyme catalyzes the rate-limiting step in the hepatic biotransformation of cholesterol into cholic acid (CA) and chenodeoxycholic acid (CDCA). As an FXR agonist, cafestol indirectly down-regulates CYP7A1 expression. Furthermore, bile acid synthesis may be also affected by a direct inhibitory effect of cafestol on CYP7A1. This effect may be due to cafestol’s structural similarity to oxysterols^16^.

The FXR-cafestol binding was experimentally evaluated by fluorescence spectroscopy, circular dichroism, and isothermal titration calorimetry, using a mutant stabilized ligand-binding domain (LBD) of human FXR, and complemented by molecular docking and molecular dynamics simulations (MDS) to characterize the protein-ligand interactions in the complex at the molecular level, which showed an interaction occurring in the close environment of Trp454^9^. Although the binding mode between cafestol and FXR has already been investigated^9^, there are no available experimentally determined 3D structures (e.g., X-ray crystallography, electron microscopy, and NMR spectroscopy) of the FXR-cafestol complex at the Protein Data Bank (PDB)^17^. Moreover, the binding mode of cafestol with CYP7A1 are unknown. Furthermore, there are different cafestol derivatives described in the literature^18–20^ whose hypercholesterolemic potential associated with FXR and CYP7A1 modulation has not yet been investigated.

Cafestol (1) undergoes thermal degradation during the coffee roasting process, mainly forming dehydration derivatives, such as 15,16-dehydrocafestol (∆^15,16^) (**2**), cafestal (an aldehyde, **3**), and its tautomer, 16,17-dehydrocafestol (∆^16,17^) (**4** and **5**, *E* and *Z* isomers, respectively) (Fig. 1)^18,21^. These degradation products, resulting from the loss of a water molecule relative to the tertiary hydroxyl group at the C-16 position of cafestol, preserve the *ent*-kaurane skeleton and are transferred to unfiltered beverages, along with other lipids, which may present higher concentrations than the original diterpene, depending on the degree of roasting (medium and dark)^18,22^.

Although these compounds are found in roasted coffee, there is a notable gap in the literature regarding their isolation. Furthermore, few synthetic cafestol derivatives have been reported, so the structure-activity relationship of this diterpene remains poorly understood. Lam et al.^20^ showed that fully hydrogenating cafestol’s furan ring produces an inactive product (**6**) (Fig. 1), highlighting the essential role of this aromatic system in stimulating glutathione S-transferase, a key enzyme in the chemopreventive effect of cafestol. However, no study has evaluated the effect of structural modifications of this *ent*-kaurane diterpene targeting hypercholesterolemic activity.

Natural product metabolites are important sources for discovering new bioactive compounds because they offer wide structural variability. However, the pharmacokinetics of these compounds are complex and reflect discrepancies between results in preclinical and clinical trials^23,24^. Metabolites may exhibit same or different bioactivities from the parent compound, especially those from phase I metabolism, considered a bioactivating step^25,26^.

CYP phase I metabolites of cafestol, produced through hydroxylation reactions (with or without subsequent oxidation of the alcohol group to a ketone), such as 2-oxo-cafestol (**7**), 6-oxo-cafestol (**8**), 6,12-hydroxy-cafestol (**9**–**12**), and 6-hydroxy-cafestol (**13**–**14**) (Fig. 1), have been identified in studies utilizing a zebrafish (*Danio rerio*) model^19^. Many of the members of the zebrafish CYP complex families 5–51 are direct orthologs of human CYPs, including CYP7A1^27^. To date, there are no reports or proposals of bioactivity for these metabolites. Although human cafestol metabolites are mainly described as glucuronide conjugate or sulfate salts forms, these phase II metabolites are commonly inactive, water-soluble, and consequently easily excretable.

Computational methods, such as molecular docking, are useful for the preliminary investigation of compounds whose biological activity has not been described. Besides traditional ligand-site focused docking approach, cavity-guided docking offers an alternative to evaluating other possible interaction sites in the protein structure beyond the orthosteric one. However, molecular docking generally considers the target protein as a static model, not reflecting the complexity of biological systems. Thus, molecular dynamics simulations (MDS) can be used to understand the dynamic behavior of the protein-ligand complex^28^. When integrated with molecular docking/dynamics simulations, other computational methods, such as pharmacophore mapping and ADME (absorption, distribution, metabolism, and excretion) analysis of compounds under study, allow visualization of structural features essential for interaction with target proteins and the pharmacokinetic profile, respectively^29^.

Considering the role of investigating the hypercholesterolemic potential of cafestol structural analogues for deep understanding between unfiltered coffee consumption and the development of dyslipidemia, this study aimed to explore the potential binding modes of cafestol (**1**), cafestol roasting derivatives (**2**–**5**), hydrogenated cafestol (**6**), and cafestol phase I metabolites from zebrafish (**7**–**14**) (Fig. 1) with two human protein-targets, i.e., the FXR nuclear receptor and the CYP7A1 enzyme, through molecular docking and molecular dynamics simulations (MDS) guided by cavity detection.

## Computational methods

### Proteins and ligands sources

The primary structures (i.e., linear amino acid residue sequence) of the protein targets were retrieved from the UniProt database (https://www.uniprot.org/)^30^. The sequence of the human (*Homo sapiens*) bile acid/farnesoid X receptor (FXR) is available in UniProt under the code Q96RI1 (NR1H4_HUMAN) (https://www.uniprot.org/uniprotkb/Q96RI1/), containing 486 amino acid residues. According to other members of the nuclear receptor superfamily, FXR consists of the A/B (N-terminal) region containing an activation function-1 (AF1) domain, the central C region that has a DNA-binding domain (DBD, residues 134–209), while the D region hinge domain links the DBD and the ligand-binding domain (LBD, residues 262–486), which is located at the E (C-terminal) region, including the activation function-2 (AF-2)^31^.

The sequence of the human (*Homo sapiens*) cytochrome P450 7A1 (CYP7A1) enzyme, also known as cholesterol 7-α-hydroxylase, is available in UniProt under the code P22680 (CP7A1_HUMAN) (https://www.uniprot.org/uniprotkb/P22680/). The protein sequence contains 504 amino acid residues, characterized by an N-terminal transmembrane anchor domain (residues 4–24), which is critical for its localization to the endoplasmic reticulum membrane, and a catalytic domain (residues 25–504) containing the heme (Fe-protoporphyrin IX) prosthetic group (cofactor) at the catalytic site^32^.

The experimental tertiary (three-dimensional, 3D) structures of the protein targets were retrieved from the Research Collaboratory for Structural Bioinformatics Protein Data Bank (RCSB PDB) database (https://www.rcsb.org/)^17^. The selection of 3D structures of the target proteins from PDB was based mainly on the presence of ligands with a steroid nucleus to establish structural correlations and modulatory action against the targets, i.e., agonist effect on the receptor (FXR) and inhibitory effect on the enzyme (CYP7A1).

Therefore, for FXR, obeticholic acid (OCA), also known as 6α-ethyl-chenodeoxycholic acid (6-ECDCA), a potent and selective FXR agonist (EC_50_ = 99 nM)^33^, was considered. The experimental 3D structure of the FXR-OCA complex is available under the PDB code 1OSV (method: X-ray diffraction, resolution: 2.50 Å, organism: *Rattus norvegicus*)^34^, corresponding to the LBD from rat FXR bound to OCA (PDB ligand code: CHC).

For CYP7A1, 7-ketocholesterol (7KCh), a competitive inhibitor of CYP7A1 (IC_50_ ∼1 µM)^32^, was considered. The experimental 3D structure of the CYP7A1-7KCh complex is available under the PDB code 3V8D (method: X-ray diffraction, resolution: 1.90 Å, organism: *Homo sapiens*), corresponding to the human CYP7A1 enzyme, including the heme cofactor (PDB ligand code: HEM), bound to 7KCh (PDB ligand code: 0GV).

The experimental 3D structure of cafestol was retrieved from the Cambridge Crystallographic Data Centre (CCDC) database of crystal structures of organic compounds (https://www.ccdc.cam.ac.uk/)^35^. There is only one crystallographic structure of cafestol in CCDC named “cafestol monohydrate”, under deposition number 1,434,207, and database identifier (CSD entry) OPISOX^36^.

### Comparative modeling of proteins

**Fig. 2:**
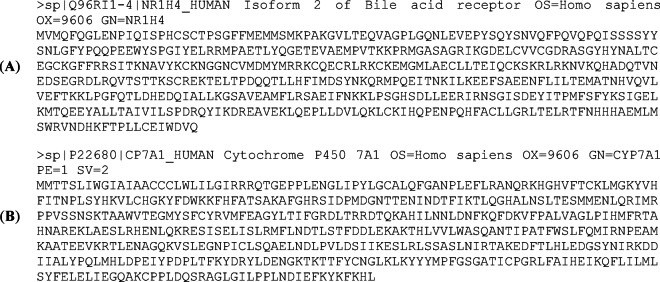
Amino acid residue sequences in FASTA format available from UniProt of the human target proteins: (**A**) FXR (UniProt code: Q96RI1-4 – NR1H4_HUMAN) and (**B**) CYP7A1 (UniProt code: P22680 – CP7A1_HUMAN). Amino acid residues in FASTA format are represented using the standard IUB/IUPAC one-letter code.

Comparative modeling (also known as homology modeling) is a technique used to build three-dimensional (3D) protein models from a target sequence and a 3D template sharing a minimum of 30% of sequence similarity^37^. This approach was adopted to obtain complete proteins after observing missing or modified atoms or residues in the structures of the rat FXR LBD (PBD ID: 1OSV)^34^ and human CYP7A1 (PBD ID: 3V8D)^32^. The human FXR and CYP7A1 sequences in FASTA format were obtained from the UniProt database (https://www.uniprot.org/), from the codes P22680 and Q96RI1-4 (Fig. 2), respectively.

The template used for FXR modeling was from rat origin (PDB 1D: 1OSV, method: X-ray diffraction, resolution: 2.50 Å, organism of the LBD: *Rattus norvegicus*)^34^, corresponding to the LBD bound to 6α-ethyl-chenodeoxycholic acid (6-ECDCA), also known as obeticholic acid (OCA). The U.S. Food and Drug Administration (FDA) approved OCA for treating primary biliary cholangitis in 2016, but ongoing monitoring continues following reports of serious liver injury since 2024^38^. The enzyme was modeled using the sole available template complexed with an inhibitor (PDB: 3V8D)^32^. Models construction and validation (via Ramachandran plot analysis) were carried out utilizing the SWISS-MODEL server (https://swissmodel.expasy.org/)^39^.

### Modeling of ligands structures

The 3D structure of the cafestol ligand as a CIF format was downloaded from the CCDC database under CSD code OPISOX (cafestol monohydrate)^40^. An isolated diterpene structure was converted from CIF to MOL2 format using the CCDC Mercury program (https://www.ccdc.cam.ac.uk/solutions/software/free-mercury/)^41^. The structure curation, including correction of bond orders, and addition of missing hydrogens, was carried out with the Spartan’10 program (https://www.wavefun.com/) (Wavefunction, Inc.). The Merck molecular mechanics force field (MMFF94), available in this program, was adopted for geometry optimization.

The 3D structures of the other ligands, including cafestol roasting degradation products (2–5), hydrogenated cafestol (6), and zebrafish cafestol phase I metabolites (7–14), were constructed from the cafestol diterpene structure (1) with a subsequent geometry optimization step with MMFF94. All structures were saved in both MOL2 and PDB formats. The SMILES (simplified molecular input line entry system) linear notation of each ligand (obeticholic acid, OCA; 7-ketocholesterol, 7KCh; and 1–14) is provided in Supplementary Information (Table S1).

### Protein cavity prediction

The detection of pockets in the 3D structures of both proteins (FXR and CYP7A1) was performed on the CavityPlus (v. 2022) web server (http://pkumdl.cn:8000/cavityplus/)^29^. The Cavity module of CavityPlus maps the three-dimensional surface of the protein, allowing the detection of pockets, using a geometry-based binding site detection method. In addition, the Cavity module analyzes the potential interaction of each detected cavity with ligands using criteria such as druggability (CavityDrugScore) and ligandability (CavityScore) scores.

The PDB files of the modeled human proteins (FXR and CYP7A1) were loaded (“Step 1: Provide a protein”) for cavity searching in the selected protein chain (“Step 2: Select chains”), without considering the ligand structure (i.e., in the “Step 3: Choose mode” the option “Use Ligand Mode” was set off). Using this default mode, the Cavity module detected potential binding sites of the whole protein. The remaining search parameters (“Step 4: Advanced parameters”) were kept in the default option for common cavity (i.e., cavity for drug-like compounds) (Table S2, Supplementary Information). The three best predicted cavities were selected considering the ligandability criterion based on the predicted (maximum and average) affinity (Pred Max p*K*_d_ and Pred Ave p*K*d). Among the selected cavities, at least one allosteric site was included, following the prediction by the CorrSite module of CavityPlus.

### Docking and pose analysis

The DockThor web server (https://dockthor.lncc.br/v2/)^42^ and AutoDock (v.4.2) program (https://autodock.scripps.edu/)^43^ were used for molecular docking of the ligands against the FXR receptor. Docking against CYP7A1 was caried out only using AutoDock, due to its compatibility with the heme group of the CYP enzyme. Grid box center (xyz coordinates) and grid box size (*a*, *b*, *c* lengths of the box) were based on the geometric data of predicted cavities in CavityPlus. The poses (i.e., biding modes or solutions) and intermolecular interactions in the protein-ligand complexes were analyzed with the BIOVIA Discovery Studio Visualizer (DSV) (v.2021) program (https://discover.3ds.com/).

### Structures preparation for docking

In the docking procedure using AutoDock on both proteins, all co-crystallized ligands and water molecules were removed, polar hydrogens were added, and Kollman charges were assigned using the AutoDockTools (ADT) interface. For CYP7A1, the heme group cofactor was maintained. Regarding the ligands, all rotatable bonds were kept free and the Gasteiger charges were assigned. Lamarckian genetic algorithm (LGA) was selected to explore the ligand’s translational, rotational, and conformational space. In the DockThor server, ionization states of the FXR residues were adjusted to physiological media (pH = 7). Ligands rotatable bonds were treated as free, followed by the addition of hydrogen atoms.

Among the roasting derivatives and phase I metabolites, fourteen ligands were constructed, including the *E*/*Z* diastereomers of 16,17-dehydrocafestol (**4** and **5**), 6,12-hydroxy-cafestol (**9–12**), and 6-hydroxy-cafestol (**13–14**). In addition, a derivative with a hydrogenated furan ring, i.e. hydrogenated cafestol (**6**), was included to investigate the importance of the aromatic system for protein-ligand interactions (Fig. 1).

Additionally, we attempted to construct and optimize 13,16-dehydrocafestol by molecular mechanics (MMFF94, Spartan), a compound that has been reported in the literature based on gas chromatography results, though it is likely an artifact^18,21^. However, we were unsuccessful, likely because synthesizing compounds with a double bond at the bridgehead position of a small bridged ring system is challenging due to Bredt’s rule, though there are some known exceptions called anti-Bredt olefins.

The validation of the docking protocol was achieved by redocking (i.e., self-docking), comparing the predicted and experimental poses of the original co-crystallized ligand based on the root-mean square deviation (RMSD) metric, with values ≤ 2.0 Å being accepted^45^. The coordinates of the centroids of the reference ligands obeticholic acid (OCA; x = 12.18 Å; y = 37.39 Å; z = 16.83 Å) and 7-ketocholesterol (7KCh; x = 11.265 Å; y = 7.469 Å; z = − 42.442 Å) of the the FXR and CYP7A1 complexes, respectively, were used as the center of the grid box. The grid box size and grid spacing were defined in AutoDock (44 ⋅ 44 ⋅ 44 Å³ and 0.375 Å) and DockThor (20 ⋅ 20 ⋅ 20 Å³ and 0.25 Å).

### Molecular dynamics simulations

Molecular dynamics simulations (MDS) were carried out with the GROMACS package (v.5.1.4 and v.2019)^46^ using the CHARMM36 force field^47^. This work followed the protocol established in our previous studies^48^. Force field parameters for ligands were generated through the CGenFF server using its default settings^49^. Each protein-ligand complex was placed in a cubic simulation box (100.8 ⋅ 89.1 ⋅ 77.6 Å³), solvated with TIP3P water, and neutralized by the addition of four Cl⁻ ions.

Energy minimization was conducted in two steps: first, a steepest descent algorithm was applied until the maximum force reached 1000 kJ·mol⁻¹·nm⁻¹, followed by a conjugate-gradient algorithm until convergence at tolerance of 100 kJ·mol⁻¹·nm⁻¹. The systems were submitted to two sequential equilibration steps with positional restraints applied to protein and ligand. First, as NVT ensemble (canonical) was performed for 1 ns at 300 K using the V-rescale thermostat with separate coupling groups for the protein and non-protein atoms (tau_t = 0.1 ps). Subsequently, an NPT ensemble (isothermal-isobaric) was conducted for 1 ns at 300 K and 1 bar using the same V-rescale thermostat and the Parrinello–Rahman barostat (tau_p = 2.0 ps, compressibility = 4.5 × 10⁻⁵ bar⁻¹). In both steps, hydrogen bond lengths were constrained using the LINCS algorithm (lincs_order = 4, lincs_iter = 1). Long-range electrostatic interactions were calculated using the PME method with a Fourier grid spacing of 0.16 nm and fourth-order cubic interpolation. Short-range van der Waals and Coulomb interactions employed a 1.2 nm cutoff with the Verlet cutoff scheme, and long-range van der Waals corrections were applied using the energy-pressure correction (EnerPres) method.

Production MDS were performed in the NPT ensemble in triplicate, each generating 200ns trajectories with a 2 fs integration timestep using the leap-frog integrator. Trajectory coordinates were saved every 10ps in compressed format, while energies and log files were updated at the same frequency.

The analyses of the MDS trajectories using root-mean-square deviation (RMSD) and root-mean-square fluctuation (RMSF) calculations were carried out using the “gmx rmsd” and “gmx rmsf” tools, respectively. Other trajectory analyses, including hydrogen bonding (H-bond) occupancies were computed with a 0.4 nm donor-acceptor distance cutoff, using the hbmap2grace package, retaining interactions with a persistence greater than 5% of the simulation time. All plots were generated using XMGRACE (v.5.1.19)^50^.

Principal component analysis (PCA) was employed to investigate the conformational dynamics of the complexes based on the Cα-atoms coordinates, following a previously described protocol from our group^51^. This analysis was carried out using the MDAnalysis library in conjunction with the publicly available “PCAauto” script^52^. The first two principal components (PC1 and PC2), which encompass most of the variance, were selected for free energy landscape (FEL) analysis via the inference of connectivity of states (InfleCS) method^53,54^. The FEL maps were built along the principal components (PCs) derived from the clustering object, consistent with our established procedure. FEL calculations were performed with the “InfleCS-analysis” Python script (https://github.com/brendaferrari/InfleCS-analysis).

The protein-ligand binding free energies (ΔG_bind_) were estimated via the molecular mechanics/Poisson-Boltzmann surface area (MM-PBSA) method as implemented in the “gmx_MMPBSA” tool^55^. The calculation utilized frames extracted from the most populated cluster identified in the PCA. Entropic contributions (− TΔS) were determined using the interaction entropy method within the “MM-PBSA.py” script (with the following parameters: PBRadii = 1, interaction_entropy = 1, ie_segment = 50, and temperature = 300 K). All molecular visualizations were prepared using the Visual Molecular Dynamics (VMD) (v.1.9.4) program^56^.

### Statistical analysis

Statistical analyses of RMSD and ΔG_bind_ from the three independent molecular dynamics simulations were performed using the software SISVAR^57^ (v.5.70) by the Scott-Knott (α = 0.05) test. For each residue of the protein-ligand complex, the RMSF values corresponding to the replicates were organized by ligand and subjected to the Scott-Knott clustering method with effect size difference consideration (Scott-Knott ESD)^59^, using a significance level of 5% (α = 0.05). Statistical analyses were performed in the R environment (https://www.r-project.org/) using the ScottKnottESD package (https://github.com/klainfo/ScottKnottESD). Distinct letters indicate a significant difference between treatments.

### Pharmacophore mapping

Pharmacophore maps were constructed in the PHARMIT server (https://pharmit.csb.pitt.edu/)^59^ based on both protein-ligand complexes: FXR (PBD ID: 1OSV) and CYP7A1 (PDB ID: 3V8D). CavPharmer, a module of CavityPlus, was used to identify the most relevant pharmacophore features (e.g., H-bond donor, H-bond acceptor, hydrophobic, and electrostatic) detected in PHARMIT for the structures of the co-crystallized ligands (OCA and 7KCh), as well as for cafestol (**1**) and its main roasting derivative 15,16-dehydrocafestol (**2**). No virtual screening in libraries was conducted. The analysis was focused on pharmacophore mapping for structural comparison and correlation between steroidal and *ent*-kaurane scaffolds.

### ADME profile of Cafestol roasting derivatives

Physicochemical, lipophilicity, and pharmacokinetic parameters related to the absorption, distribution, metabolism, and excretion (ADME) process were estimated from molecular structure (SMILES format) with the SwissADME server^60^ to investigate the pharmacokinetic profile of two cafestol derivatives, namely 15,16-dehydrocafestol (2) and cafestal (3). These two compounds generated during roasting were chosen from among all the ligands studied because they are the only ones quantified to date in coffee beverages. The in silico ADME parameters calculated were compared with data available in the literature for cafestol (**1**)^61^.

## Results and discussion

### Analysis of the 3D models of target proteins

**Fig. 3:**
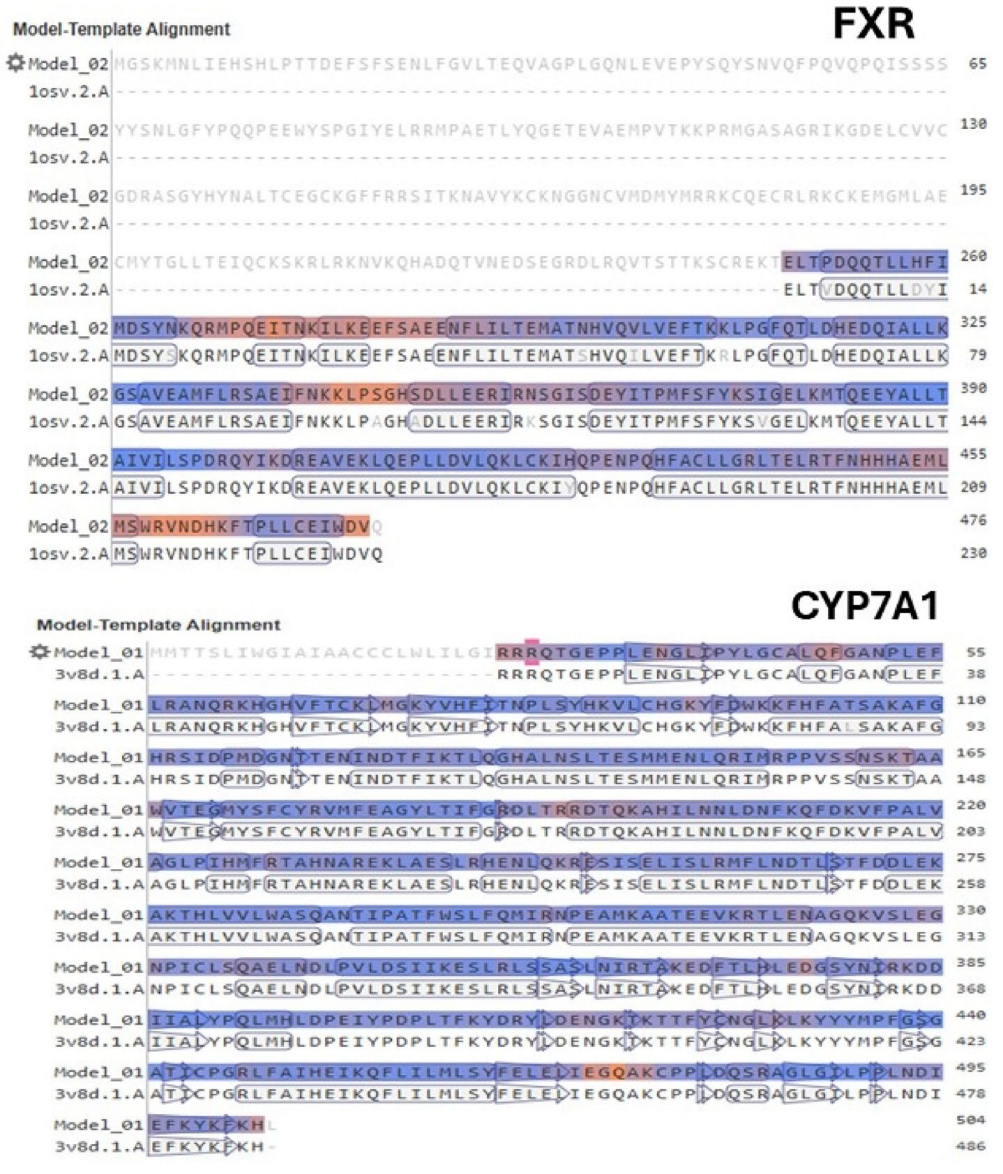
Model-template alignments of the sequences of the human target proteins (FXR UniProt: Q96RI1-4 – NR1H4_HUMAN) and CYP7A1 UniProt: P22680–CP7A1_HUMAN) with the sequences of the corresponding templates (PDB ID: 1OSV and PDB ID: 3V8D) using the SWISS-MODEL.

In the SWISS-MODEL server, the 3D models of the target proteins were constructed by comparative modeling considering the model-template alignments (Fig. 3) by aligning the sequences of the target proteins (FXR and CYP7A1) with the corresponding sequences of their templates. For FXR, the selected template sequence (PDB ID: 1OSV) showed partial coverage (48%) and high identity (94.76%) with the target sequence (UniProt: Q96RI1-4). This partial coverage was expected, since the sequence of the PDB structure (1OSV) corresponds only to the LDB region (residues 241–469), while the UniProt sequence (Q96RI1, NR1H4_HUMAN) corresponds to the complete receptor (residues 1–486). In the case of CYP7A1, the sequence of the selected template (PDB ID: 3V8D) exhibited high coverage (95%) and high identity (99.79%) with the target sequence (UniProt: P22680).

**Fig. 4:**
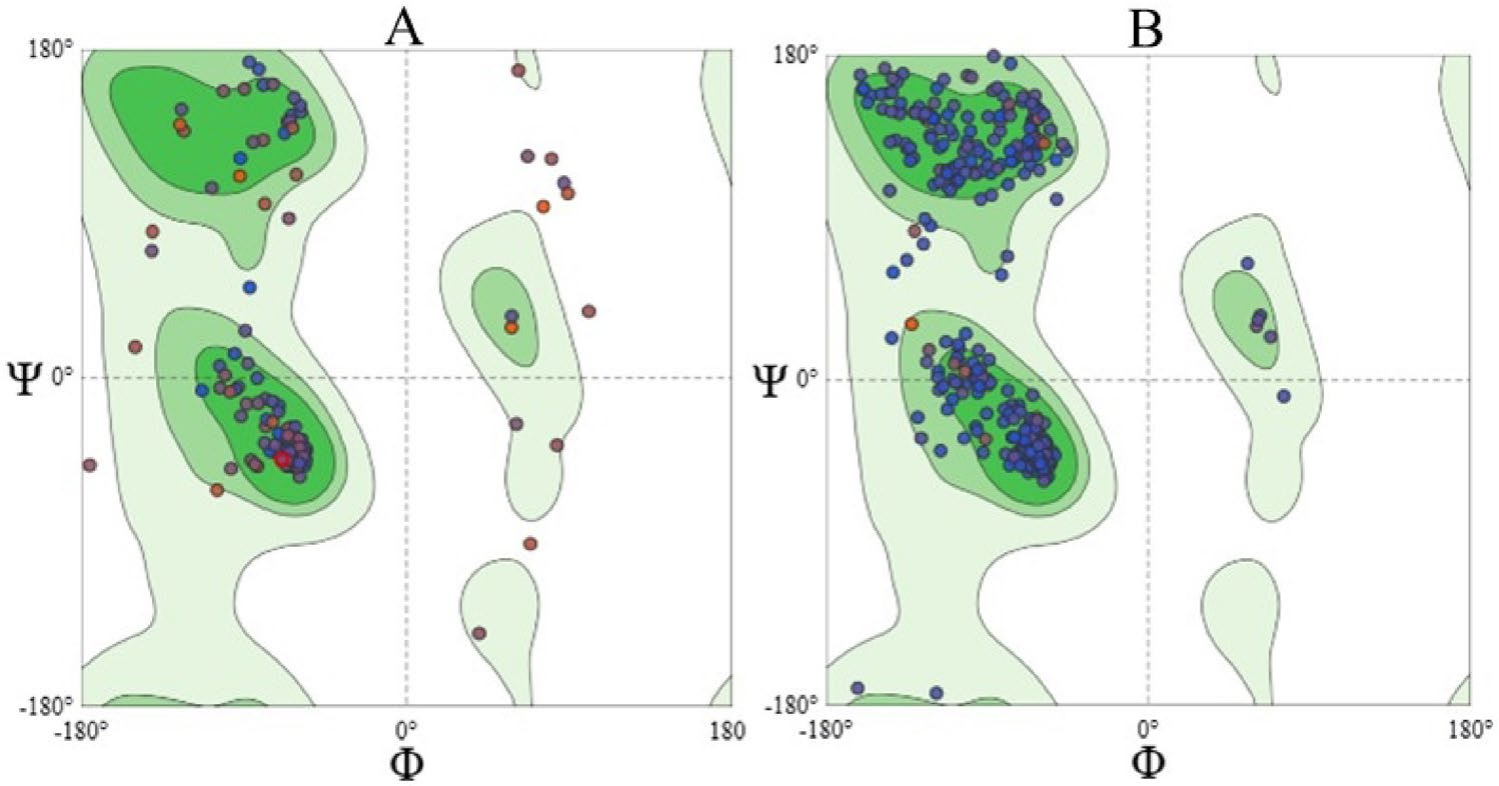
Ramachandran plots of the human 3D models of FXR (**A**) and CYP7A1 (**B**) proteins generated by the MolProbity tool in the SWISS-MODEL server. Dark green and light green exhibit the most favorable and favorable regions, respectively.

The constructed 3D protein-ligand models are represented in Figure S1 (Supplementary Information) and they are available in the ModelArchive (modelarchive.org) repository with the accession codes ma-japwz (FXR model) and ma-qyq0l (CYP7A1 model). The Ramachandran plots obtained using MolProbity (v.4.4) available in the SWISS-MODEL server demonstrated the quality of the models, indicating that most residues were in favored regions for both targets (Fig. 4), with favorable region percentages of 90.71% for FXR and 98.53% for CYP7A1.

In summary, the human CYP7A1 model demonstrates superior overall quality compared to the human FXR model. This expected result is attributable to the use of a human enzyme template with higher resolution (PDB ID: 3V8D, 1.90 Å), whereas the receptor template is derived from rat and exhibits lower resolution (PDB ID: 1OSV, 2.50 Å). These differences are reflected accordingly in the resulting models.

Although AlphaFold2 (AF2) uses artificial intelligence (AI) and machine learning (ML) to compute with high accuracy protein models, which are available in the RCSB website along with PDB experimental models, it considers only one conformational state of the protein and does not contain ligands and cofactors in models^62^. Therefore, the preference for comparative modeling was supported by the necessity of establishing structural comparisons between tested and reference ligands sharing the same steroidal skeleton. For the sake of comparison, the superpositions between the target proteins models by comparative modeling and predicted by AF2 are presented in Figure S2 (Supplementary Information).

**Fig. 5:**
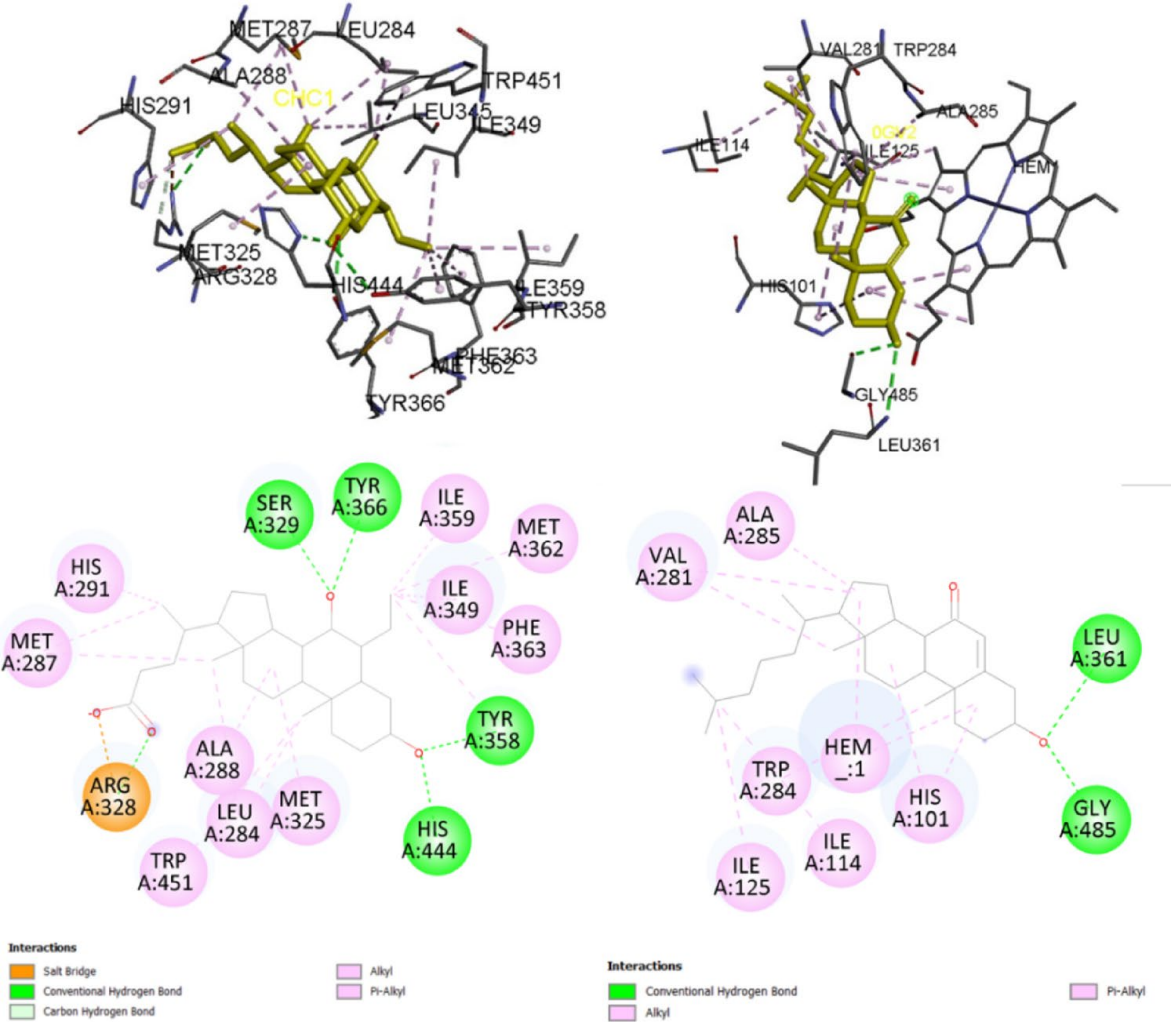
3D (up) and 2D (down) representations of the intermolecular interactions observed in the ligand binding site of the modeled protein-ligand complexes of FXR-OCA (left) and CYP7A1-7KCh (right).

The protein-ligand interactions detected in the modeled FXR-OCA and CYP7A1-7KCh complexes (Fig. 5) were compared with those observed in the PDB experimental structures 1OSV and 3V8D, respectively.

In the modeled human FXR-OCA complex (Fig. 5), most of the protein-ligand interactions detected within up to 5.5 Å are the same as those observed in the X-ray complex (1OSV), including a salt bridge (ionic and H-bond) between the OCA carboxylate group and the Arg328 guanidinium group, H-bond between the two hydroxyl groups of OCA and the phenolic hydroxyl groups of Tyr358 and Tyr366, and the imidazole ring of His444, along with hydrophobic contacts of the steroidal backbone and alkyl substituents of OCA with several receptor residues.

Similarly, in the modeled human CYP7A1-7KCh complex (Fig. 5), most of the interactions detected up to 5.5 Å are the same as those observed in the X-ray complex (3V8D), including two H-bonds between the 7KCh hydroxyl group and the main chain (NH and CO groups) of Leu361 and Gly485, respectively, along with hydrophobic contacts between the steroidal backbone and alkyl substituents of 7KCh with several enzyme residues (e.g., His101, Ile114, Ile125, Val281, Trp284, and Ala285) and carbon atoms of the heme cofactor. However, a H-bond between the carbonyl oxygen atom of 7KCh and a water molecule whose oxygen atom is coordinated to the Fe-atom of the heme group in the X-ray structure (PDB ID: 3V8D) was not detected in the modeled complex, which was expected, since all water molecules were excluded in the comparative modeling step.

### Determination of docking sites by cavity prediction

Table 1 summarizes the data from the three top cavities predicted by the Cavity module of the CavityPlus server for each protein target. These three best predicted cavities for FXR and CYP7A1 were selected from a total of seven and twenty-three, respectively (Figure S3, Supplementary Information). Table 1 includes predicted affinity data, such as complex dissociation constant (p*K*_d_), maximum p*K*_d_ (p*K*_d max_), and average p*K*_d_ (p*K*_d ave_); and cavity geometric data, such as center coordinates (xyz, Å), edge sizes (*a*, *b*, and *c*, Å), and volumes (Å^2^).

Cavity #1 of each protein target, FXR and CYP7A1, corresponds to the orthosteric site, i.e., the receptor’s endogenous agonist binding site and the enzyme’s substrate biding (active) site, respectively. Consequently, these cavities aligned with the position of the respective reference ligands, i.e., OCA (FXR agonist) and 7KCh (CYP7A1 inhibitor). The data of the three top cavities from both targets are summarized in Tables S3–S8 (Supplementary Information). In the CorrSite module for searching allosteric sites, both cavities #2 and #3 were also identified as allosteric sites for both target proteins (FXR and CYP7A1), considering the computed values of ID score (Tables S4–S8, Supplementary Information).

For FXR, residues Asn21, Asn53, Val57, and Ser105, located within cavity #1, were missing from the templates. In the case of orthosteric site of CYP7A1, only a single mismatch was identified, with Thr104 in the target sequence replaced by Leu104 in the template. All these original residues were incorporated into the comparative protein models.

**Table 1:**
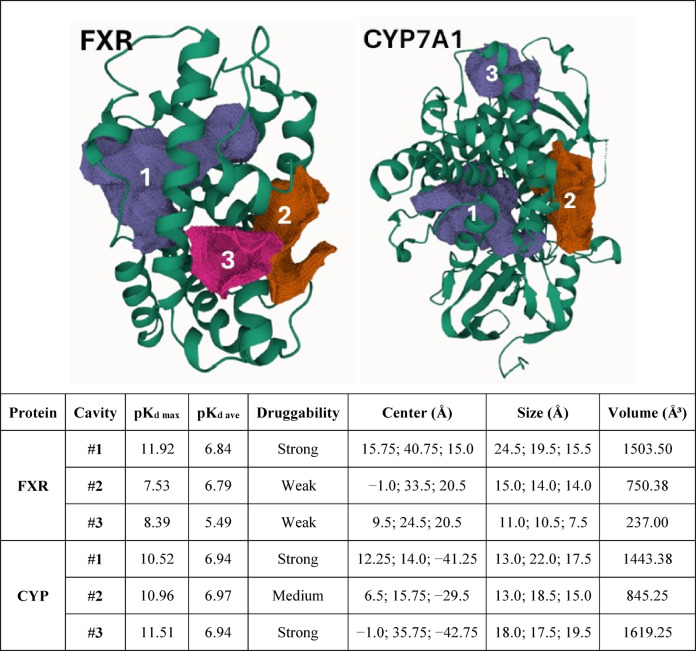
Ranking of the three best predicted cavities (Cav) by the CavityPlus web server of the target proteins, the farnesoid X receptor (FXR) and the cholesterol-7-α-hydroxylase enzyme (CYP7A1), along with the predicted values of complex dissociation constant (pKd), maximum pKd (pKd max), and average pKd (pKd ave), and geometric data of the cavities: center coordinates (xyz, Å), edge sizes (*a*, *b*, and c, Å), and volumes (Å^2^).

### Docking protocol validation

**Fig. 6:**
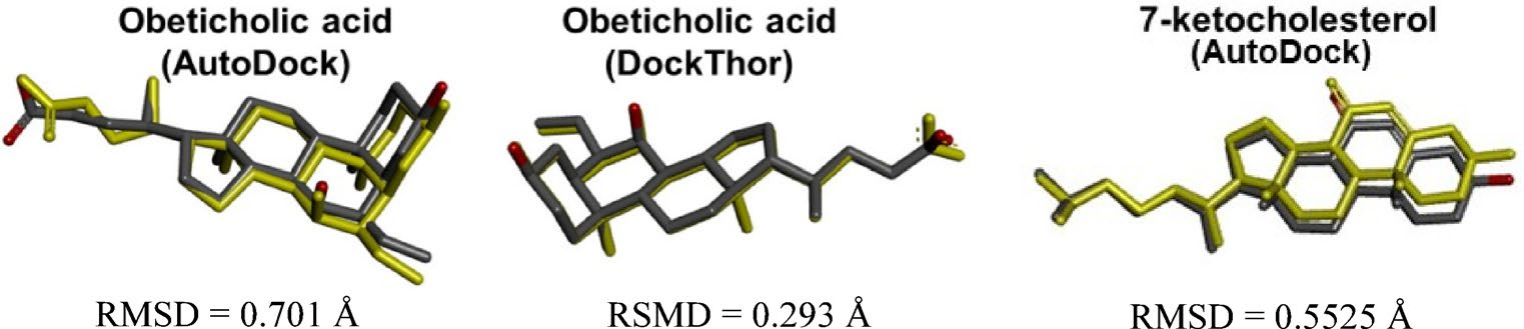
Superposition of the three-dimensional structures of the original FXR (PDB: 1OSV) and CYP7A1 (PDB: 3V8D) obeticholic acid (OCA) and 7-ketocholesterol (7KCh) ligands, respectively, with the corresponding poses obtained by redocking (yellow) using the AutoDock and DockThor programs.

The validation of the docking protocol by redocking adhered to the criterion of RMSD ≤ 2.0 Å for both protein targets, indicating a good match between predicted and experimental poses (Fig. 6)^63^. In the complex with FXR, OCA redocking poses exhibited RMSD values of 0.701Å (AutoDock) and 0.293Å (DockThor). Interactions between OCA and Arg328 (via salt bridge), as well as H-bonds with Tyr358, Tyr366, and His444, were detected in the FXR complex by both programs (Fig. 6), consistent with observations reported in the comparative modeling results (Fig. 5).

In the complex with CYP7A1, 7KCh redocking pose (AutoDock) exhibited an RMSD value of 0.5525Å (Fig. 6). Consistent with the comparative model, hydrophobic contacts were detected between 7KCh and some enzyme residues (e.g., His101, Ile114, Ile125, Val281, Trp284, and Ala285). Notably, only one H-bond originally involving Leu361 was substituted by Ser360, while the H-bond with Gly485 remained unchanged.

The difference in RMSD values between the docking programs was expected, considering the particularities of each one for rotational, translational, and conformational search (best pose). Differences include parameterization, search algorithms, number of populations and executions, search for global and local minimum, scoring functions, in addition to the differences in ligand flexibility in each program^43,64^.

### Ligand docking into FXR and CYP7A1 pockets

Through molecular docking, we compared the binding modes (poses) of roasting derivatives and phase I metabolites of cafestol in complexes with FXR and CYP7A1, two diterpene-modulated proteins associated with hypercholesterolemia. Both classes of compounds fully preserve the pentacyclic *ent*-kaurane skeleton. Roasting derivatives are modified only in the side chain. Biological data on these compounds present in roasted beans and, consequently, in unfiltered coffee beverages, are scarce^65,66^.

The inclusion of cafestol metabolites was considered because in vivo models with active metabolic pathways, the phase I metabolites may exhibit different bioactivities than the parent compound. The binding energy values of the best poses of all ligands with FXR and CYP7A1 are described in Tables 2 and 3, respectively. Additional information related to docking results is provided in Tables S9–S17 (Supplementary Information). After corrections to the stereochemistry of some ligands, some docking runs were not performed due to the unavailability of the submissions on the DockThor server; therefore, they are listed as undetermined (n.d., no date).

**Table 2:** Binding energies of the reference ligand (OCA, FXR agonist), Cafestol (1) and its structural analogues (2–14) for the poses obtained by Docking with the AutoDock and DockThor programs in the three best predicted cavities (orthosteric site, cavity #1; and allosteric sites, cavity #2 and cavity #3) in the CavityPlus server for the farnesoid X receptor (FXR).

# ^(a)^	Binding energy (AutoDock)			Binding energy (DockThor)		
	Cavity #1	Cavity #2	Cavity #3	Cavity #1	Cavity #2	Cavity #3
OCA	– 12.930	– 3.95	– 2.41	−11.309	n.d.	n.d.
1	– 9.16	– 5.57	– 4.30	−10.062	−8.303	−8.311
2	– 9.22	– 5.48	– 5.11	−9.835	−7.759	−8.274
3	– 9.23	– 5.58	– 5.30	−9.874	−8.207	−8.280
4	– 9.01	– 6.32	– 5.34	n.d.	n.d.	n.d.
5	– 9.29	– 5.60	– 5.29	−10.095	−8.479	−7.419
6	– 9.39	– 6.16	– 4.97	−9.731	−7.589	−7.332
7	– 9.22	– 6.66	−6.44	−9.989	−7.896	n.d.
8	– 9.24	– 6.40	−6.92	−9.957	−7.989	n.d.
9	– 8.21	– 5.40	−6.72	−10.132	−7.964	n.d.
10	– 8.44	– 6.25	−6.17	−10.086	n.d.	n.d.
11	– 8.95	– 6.38	−6.26	−10.124	n.d.	n.d.
12	– 8.05	– 5.70	−6.46	−10.131	−7.000	n.d.
13	– 8.08	– 6.25	−7.06	−10.063	−7.176	n.d.
14	– 8.26	– 6.07	−6.22	−10.132	−6.984	n.d.

(a) the numbering (#) of the compounds is according to Fig. 1; OCA = obeticholic acid (ligand co-crystallized with the FXR receptor); n.d. = no date.

For FXR-ligand complexes, the criterion for selecting the best poses was the predicted affinity values by DockThor, while for AutoDock, it was the lowest binding energy values in the most populated cluster. Due to the reduced conformational freedom of the ligands, there was no great energetic and spatial variation observed between the predicted poses of the evaluated ligands. Comparing the dockings in different cavities, poses with lower energy values were consistently observed for cavity #1 (orthosteric site).

Regarding the investigation of allosteric modulation, although energy difference between cavities is not sufficient criterion to suggest some selectivity of these *ent*-kaurane ligands for the orthosteric site, cavity #1 exhibited strong drugability and a larger volume than other cavities, indicating favorable ligands accommodation and interactions in this region. However, all energy values were higher than the reference ligand OCA, a potent semisynthetic FXR agonist about 100 times more potent than the endogenous ligand chenodeoxycholic acid (CDCA)^66^. Cafestol and its derivatives were expected to have higher energy than OCA, since cafestol has lower affinity for the receptor than CDCA^10^. OCA is a drug approved for the treatment of primary biliary cholangitis and is undergoing clinical trials for nonalcoholic steatohepatitis (NASH). One of OCA adverse effects is an increase in LDL cholesterol, which may be a characteristic of steroid FXR agonists^67,68^.

**Fig. 7:**
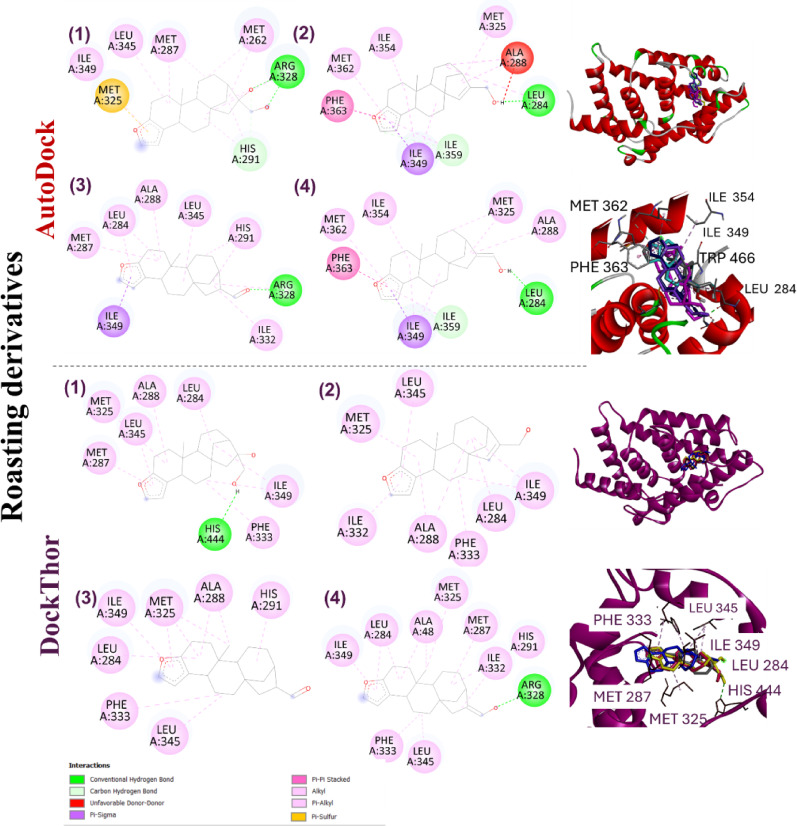
Best poses of cafestol and selected roasting derivatives and phase I metabolites on the ligand binding domain (LBD) of the farnesoid X receptor (FXR) obtained by the DockThor and AutoDock programs for cavity #1 (orthosteric site). Selected compounds: cafestol (**1**), 15,16-dehydrocafestol (**2**), cafestal (**3**), and 16,17-dehydrocafestol (**4**-*Z*).

**Fig. 8:**
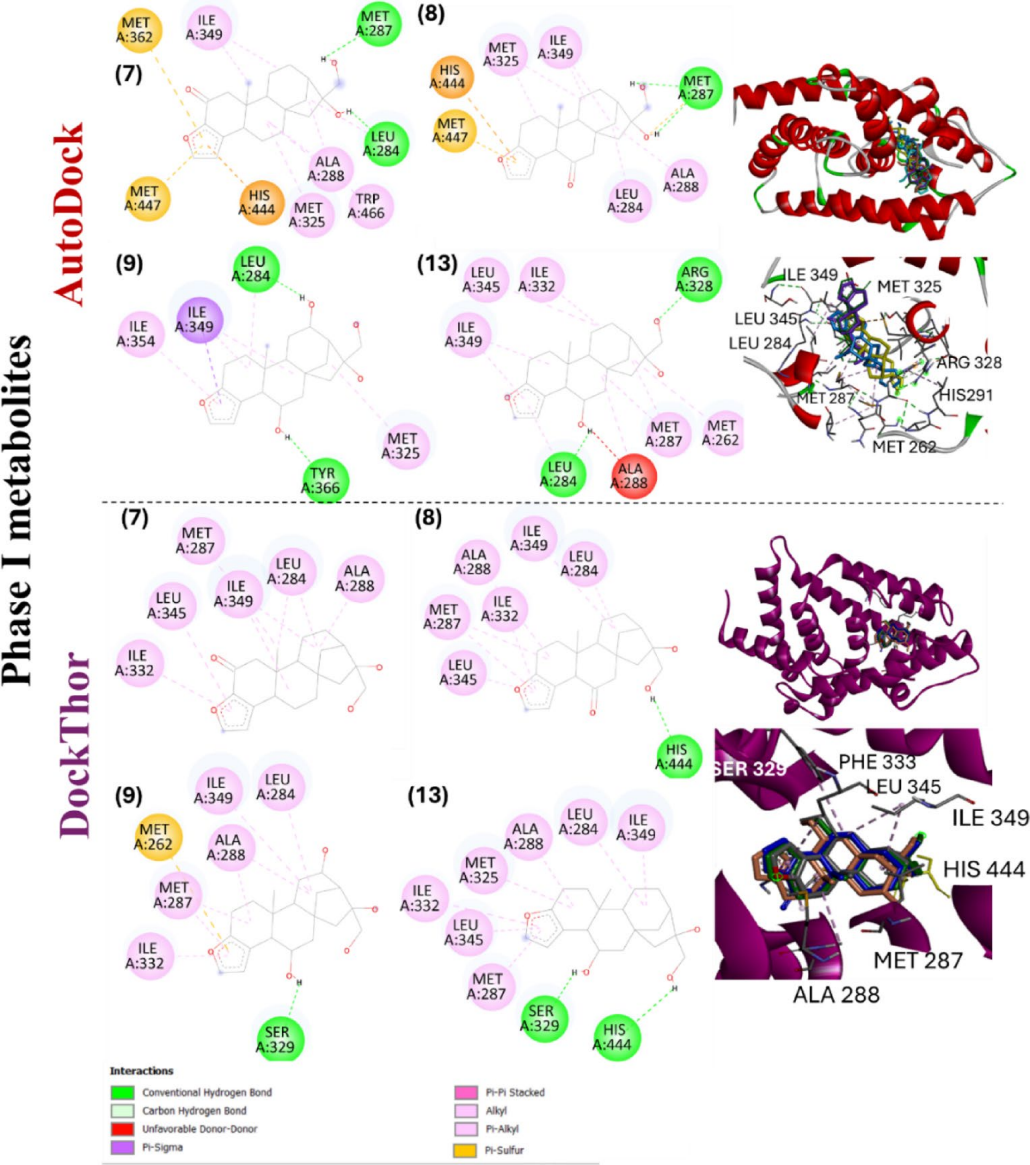
Best poses of selected cafestol phase I metabolites on the ligand binding domain (LBD) of the farnesoid X receptor (FXR) obtained by the DockThor and AutoDock programs for cavity #1 (orthosteric site). Selected compounds: 2-oxocafestol (**7**), 6-oxocafestol (**8**), 6,12-dihydroxycafestol (**19**), and 6-hydroxycafestol (**13**). The specification of the enantiomers (**9** and **13**) is according to the numbering in Fig. 1.

In the DockThor and AutoDock poses, cafestol and its analogues showed similar hydrophobic contacts in the orthosteric site, mainly due to the interactions of the three central rings of the *ent*-kaurane skeleton and furan ring of cafestol with residues Leu284, Met287, Ala288, His291, Met325, and Ile349 (Figs. 7 and 8). Comparing the AutoDock and DockThor poses of cafestol and its analogues, most H-bonds involved Arg328 (ligands 1, 3, 4, and 12) and His444 (ligands 1, 8, and 12), respectively. Cafestol and its analogues displayed a binding mode very similar to that of the OCA ligand observed earlier in the modeled complexes. Superposition of the best poses of roasting derivatives and phase I metabolites of cafestol for cavity #1 are illustrated in Figs. 7 and 8, respectively.

For complexes with FXR, different pi-type interactions from the furan of the ligands were observed, such as pi-sigma, pi-alkyl, pi-sulfur, pi-pi stacked (Figs. 7 and 8). However, compound **6** (with hydrogenated furan) exhibited a lower energy than cafestol (**1**) (Table 2), suggesting that this structural modification does not consistently impair the receptor-ligand binding affinity.

In a docking study of cafestol with FXR, Guercia et al.^9^ detected mainly hydrophobic interactions (Leu284, Ala288, Met297, and Met325), and only one H-bond with His444, while it was not detected pi-interaction through the furan ring. For roasting derivatives and phase I metabolite predictions, the same hydrophobic interactions were detected by DockThor and AutoDock (Figs. 7 and 8).

**Fig. 9:**
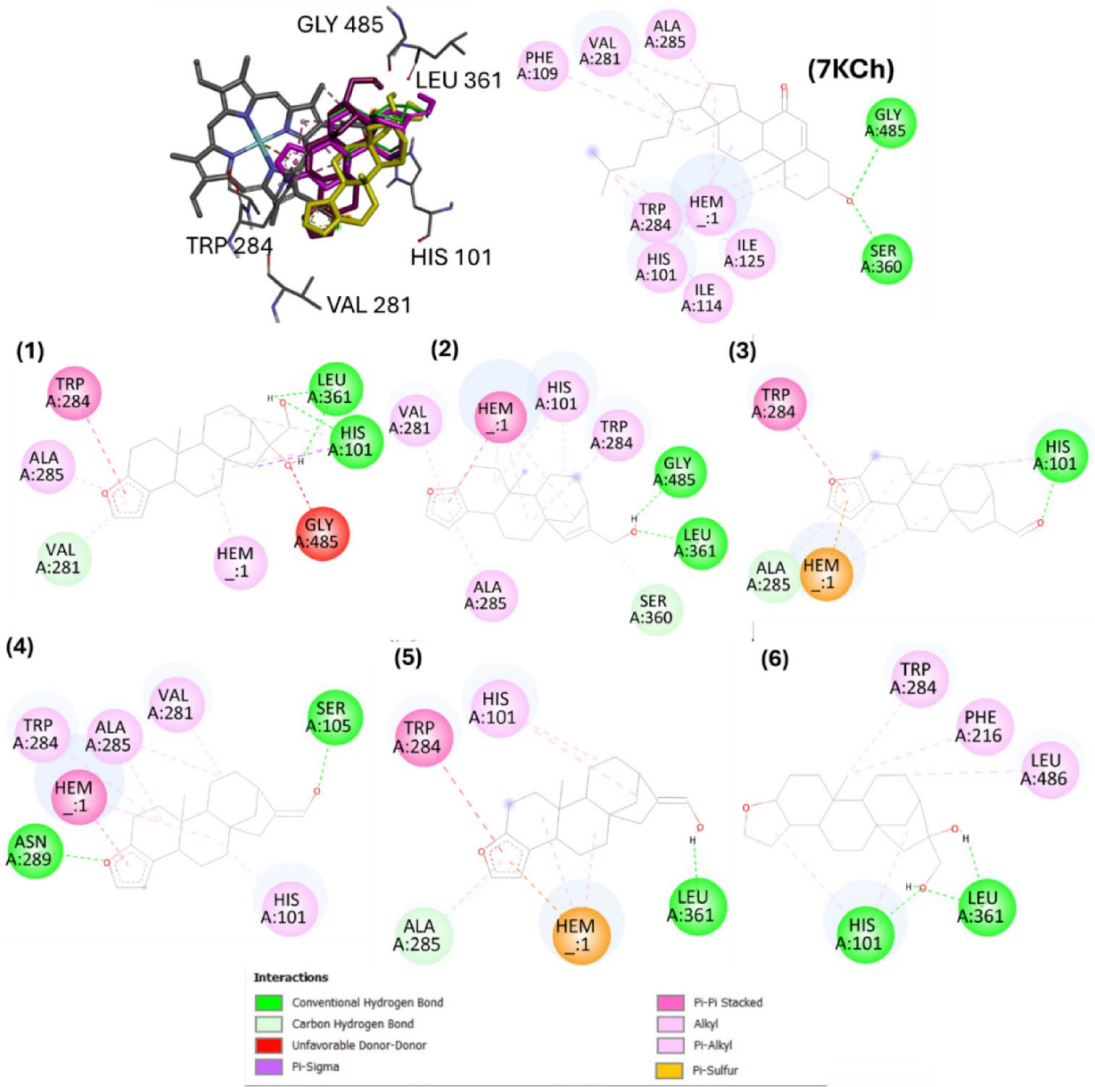
Best poses of cafestol (**1**), roasting derivatives (**2**–**5**), hydrogenated cafestol (**6**) and 7-ketocholesterol (7KCh) on the cavity #1 (orthosteric site) of cholesterol-7-alpha-hidroxylase (CYP7A1) obtained by AutoDock. The compounds were numbered according to Fig. 1.

**Fig. 10:**
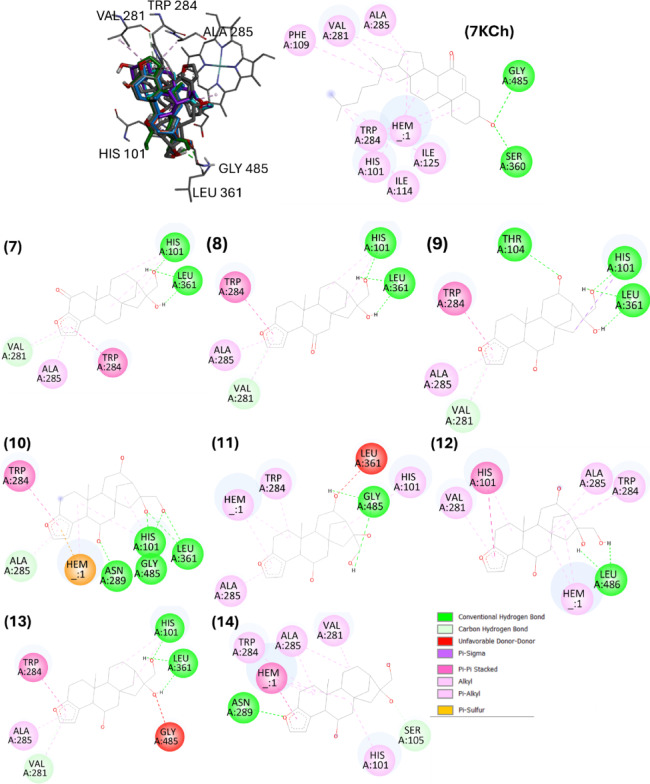
Superposition of best poses of cafestol phase I metabolites: 2-oxocafestol (**7**), 6-oxocafestol (**8**), 6,12-dihydroxycafestol (**9**–**12**) and 6-hydroxycafestol (**13**–**14**). The specification of the enantiomers (**9**–**14**) is according to the numbering in Fig. 1.

For CYP7A1, docking poses of cafestol analogues showed the lowest binding energies in cavity #1 (Table 3), corresponding to the 7KCh binding site from experimental pose. Hydrophobic contacts with His101, Val281, Trp284, and Ala285 were similar among roasting derivatives, metabolites, and 7KCh (Figs. 9 and 10). For roasting derivatives (**2–5**), pi-pi stacking with the heme cofactor occurred through the furan ring, highlighting the role of the aromatic system in the interaction with the enzyme. The absence of the aromatic ring in **6** (hydrogenated cafestol) resulted in a binding mode without interaction with the cofactor. Furthermore, compound **6** showed higher binding energy value than cafestol (**1**) (Table 2).

Phase I metabolites **10**, **11**, **12**, and **14** (mono or di-hydroxylated metabolites) interacted with the heme group, a behavior not observed for **7** and **8**, suggesting that the introduction of carbonyl groups at C6 or C2 in the cafestol structure affects the interaction profile of these *ent*-kauranes at the enzyme’s orthosteric site. Although metabolites **7** and **8** exhibited the lowest predicted docking energies among all metabolites (**7–14**) and cafestol, their lack of interaction with the heme group highlights the limitation of docking when considers only binding scores to characterize protein-ligand affinity.

**Table 3:** Binding energies of the reference ligand (7KCh, CYP7A1 inhibitor), Cafestol (**1**) and its structural analogues for the poses obtained by Docking with the AutoDock program in the three best predicted cavities (orthosteric site, cavity #1; and allosteric sites, cavity #2 and cavity #3) in the CavityPlus server for the cholesterol-7-α-hydroxylase (CYP7A1) enzyme.

#^(a)^	Binding energy (AutoDock)		
	Cavity #1	Cavity #2	Cavity #3
7KCh	– 13.98	– 8.39	– 8.89
1	– 9.63	– 6.95	– 7.04
2	– 9.82	– 6.81	– 7.28
3	– 9.85	– 7.32	– 6.40
4	– 9.86	– 7.10	– 6.58
5	– 9.72	– 6.93	– 7.51
6	– 9.24	– 7.94	– 6.33
7	– 9.78	– 6.88	– 6.92
8	– 9.72	– 6.89	– 6.85
9	– 8.80	– 5.96	– 6.65
10	– 8.25	– 6.38	– 7.58
11	– 8.29	– 6.79	– 5.84
12	– 8.33	– 6.75	– 6.58
13	– 8.42	– 6.64	– 6.89
14	– 8.46	– 6.54	– 6.36

(a) the numbering (#) of the Compounds Corresponds with Fig. 1 and 7KCh = (ligand Co crystallized with the CYP7A1 enzyme); n.d. = no date.

**Fig. 11:**
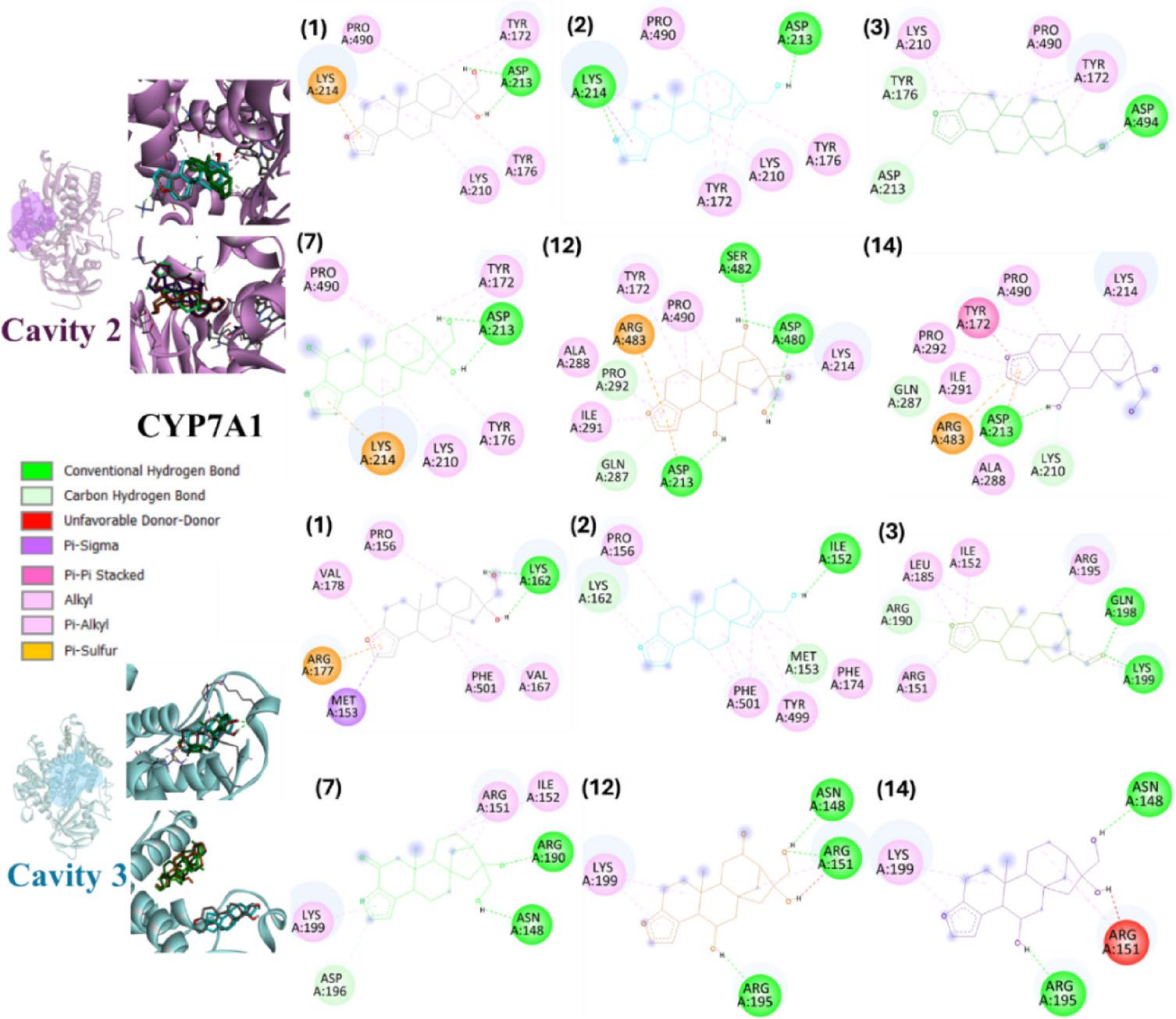
Best pose of selected roasting derivatives and phase I metabolites of cafestol (**1**) for cavities #2 and #3 of the cholesterol-7-alpha-hydroxylase enzyme (CYP7A1) obtained by AutoDock. Selected compounds: 15,16-dehydrocafestol (**2**), cafestal (**3**), 2-oxocafestol (**7**), 6,12-dihydroxycafestol (**12**) and 6-hydroxycafestol (**14**). The specification of the enantiomers (**12** and **14**) is according to the numbering in Fig. 1.

Docking on the CYP7A1 putative allosteric sites (cavities #2 and #3) corresponded to binding modes with a lower number of hydrophobic interactions compared to the orthosteric site (cavity #1). The best poses of cafestol (**1**) and its roasting derivatives (**2** and **3**), as well as metabolites 2-oxocafestol (**7**) and one stereoisomer representing 6,12-dihydroxycafestol (**12**) and 6-hydroxycafestol (**14**) are shown in Fig. 11. Specifically for cavity #2, for all ligands, the furan ring was the region responsible for the highest number of hydrophobic and pi-type interactions. However, even with the proximity of cavity #2 to the heme group (Table 1), no interactions with the cofactor were observed (Fig. 11), reinforcing the selectivity for cavity #1. Interactions of these ligands in cavity #3 (Table 1) were characterized by the low contribution of the central rings of the *ent*-kaurane skeleton, with predominant interaction via hydroxyls group and the furan ring (Fig. 11).

Unlike FXR, CYP7A1 is a protein with few molecular modeling studies. This is the first study that analyzed complexes between cafestol and its derivatives with CYP7A1 by molecular docking. The study of CYP7A1 inhibitors is not common for drug discovery, due to the association with hypercholesterolemia, also considered a negative outcome linked to enzyme deficiency^69^. 7-ketocholesterol (7KCh) is a potent competitive inhibitor of CYP7A1 reported in rat liver microsomes^70^. Tempel et al.^32^, characterized CYP7A1-7KCh complex by X-ray diffraction and established the importance of the structural rigidity of the ligand to its allocation in a site parallel to the heme group. Also, these authors highlighted the importance of interactions with the Trp284 residue for the correct position of the inhibitor.

According to the docking poses (AutoDock) in the orthosteric site of CYP7A1, all cafestol roasting derivatives interacted with the heme group and Trp284. For phase I metabolites, all interacted with Trp284, but only metabolites **10**, **11**, **12** and **14** interacted with the cofactor, suggesting that hydroxyl group behind the plane at C6 is favorable for the interaction.

CYP7A1 has been established as an important target for the hypercholesterolemic mechanism of cafestol by *in vitro* (liver microsomal fractions) and* in vivo* (APOE3Leiden mice) models^10,16^. However, subsequent clinical studies did not support this hypothesis^71^. After the consumption of coffee oil (69 mg cafestol/day) by volunteers, Boekschoten et al.^71^ indirectly monitored CYP7A1 activity by substrate (7-α-hydroxy-4-cholesten-3-one) dosage for 5 weeks..

After that, authors observed an increase in substrate level, suggesting that in humans, cafestol does not inhibit the initial step of cholesterol biotransformation into bile acids through CYP7A1. However, it was not suggested that cafestol could be rapidly metabolized and that the metabolites generated would not be inhibitors of CYP7A1.

Within the literature on cafestol metabolism, phase II metabolites, primarily as glucuronide and sulfate conjugates, have been identified as the principal forms^1^. Andriolo et al.^19^ were pioneers in describing phase I metabolites in the zebrafish water tank model using extensive characterization by high-resolution mass spectrometry combined with in silico analysis for identity confirmation of metabolites. The present study suggests, based on the comparison of the binding modes of test and reference ligands, that some cafestol phase I metabolites described in zebrafish may interact with FXR, while showing an unfavorable binding profile toward CYP7A1. Although the binding energies of compounds **1–14** for CYP7A1 cavity #1 were closeness (Table 3), the absence of interactions with heme group (compounds **7**, **8**, **9** and **13**) is highlighted as an important criterion for the discrimination of ligands profile.

FXR receptors are highly expressed in the enterohepatic system and have an important role in the regulation of cholesterol metabolism^72^. In the liver, FXR activation results in CYP7A1 inhibition. Alternatively, in the intestine, this receptor regulates lipid transport pathways, increasing serum cholesterol without downregulation hepatic bile acid biosynthesis^10,73^. In more complex organisms, such as humans, FXR may be modulated by cafestol in the intestine by alternatives pathways^10^. To elucidate the contribution of structural analogues of cafestol to hypercholesterolemia involving these target proteins, biological studies and binding assays will be essential for comparison with existing data on the diterpene.

### Molecular dynamics simulations

To elucidate the binding behavior of cafestol (**1**) and its derivative 15,16-dehydrocafestol (**2**) within the CYP7A1 and FXR binding sites previously studied by molecular docking, we performed molecular dynamics simulations (MDS) in an aqueous environment.

The structural stability of the protein was assessed by calculating the root-mean-square deviation (RMSD) of the Cα-atoms relative to the CYP7A1 holoenzyme (active unbound enzyme, i.e., apoenzyme + heme cofactor) structure (RMSD = 1.68 Å) (Figure S4 A, Supplementary Information). The complexes with ligand 1 showed RMSD values ranging from 2.0 to 2.2 Å, while those with ligand 2, ranged from 2.11 to 2.35 Å, indicating overall structural stability across all simulated cavities (#1, #2, and #3) (Figure S4 B–G and Table S18 for statistical data).

**Fig. 12:**
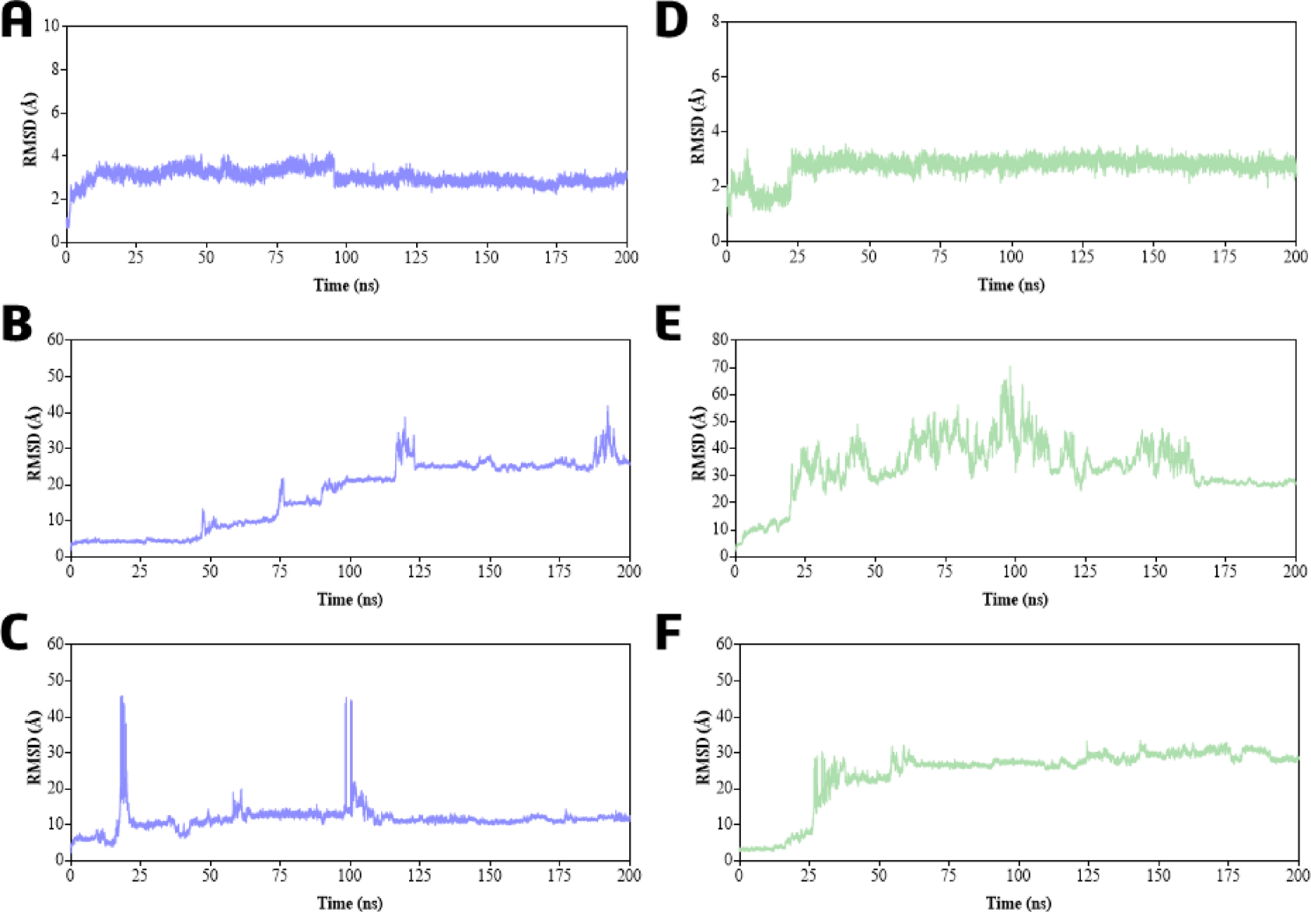
RMSD analysis of ligands atoms in molecular dynamics simulations (200 ns) on the CYP7A1 enzyme: (**A**) Cavity #1 within ligand **1**; (**B**) Cavity #2 within ligand **1**; (**C**) Cavity #3 within ligand **1**; (**D**) Cavity #1 within ligand **2**; (**E**) Cavity #2 within ligand **2**; (**F**) Cavity #3 within ligand **2**. Ligand **1** in blue, and ligand **2** in green.

Figure 12 (A–F) shows the ligands atoms RMSD analysis of cafestol (**1**) within cavities #1 (A), #2 (B), and #3 (C); and 15,16-dehydrocafestol (**2**) within cavities #1 (D), #2 (E), and #3 (F). At the CYP7A1 orthosteric site (cavity #1), both cafestol (**1**) and its dehydro-derivative (**2**) remained stably bound throughout the simulation, with ligand RMSD values of 3.0 ± 1.0 Å (Fig. 12A) and 2.7 ± 2.0 Å (Fig. 12D), respectively. In contrast, neither ligand remained stably bound in cavities #2 and #3, as evidenced by significantly higher RMSD values (see Table S19 for statistical data). For ligand 1, the RMSD reached 17.2 ± 0.7 Å (cavity #2) and 11.4 ± 5.0 Å (cavity #3). For ligand 2, the RMSD values were even larger, at 32.9 ± 12 Å (cavity #2) and 24.4 ± 14 Å (cavity #3). These results are consistent with the initial molecular docking predictions.

The root-mean-square fluctuation (RMSF) analysis revealed no significant differences in residue mobility between the holoenzyme (unbound) and ligand-bound complexes (Figure S5, Supplementary Information). Residues exhibiting fluctuations exceeding 2Å are located within the looping regions (residues 410–435) and an alpha-helix segment (residues 40–50). Notably, in the complexes involving all cavities, residues 100–125, which are part of an alpha-helix and a loop region providing access to the heme prosthetic group (cavity #1), and residues 150–175 exhibited fluctuations greater than 2Å (Figure S5 and Supplementary Information for statistical data). These fluctuations are likely associated with the entry and exit of the ligands from the cavities.

**Fig. 13:**
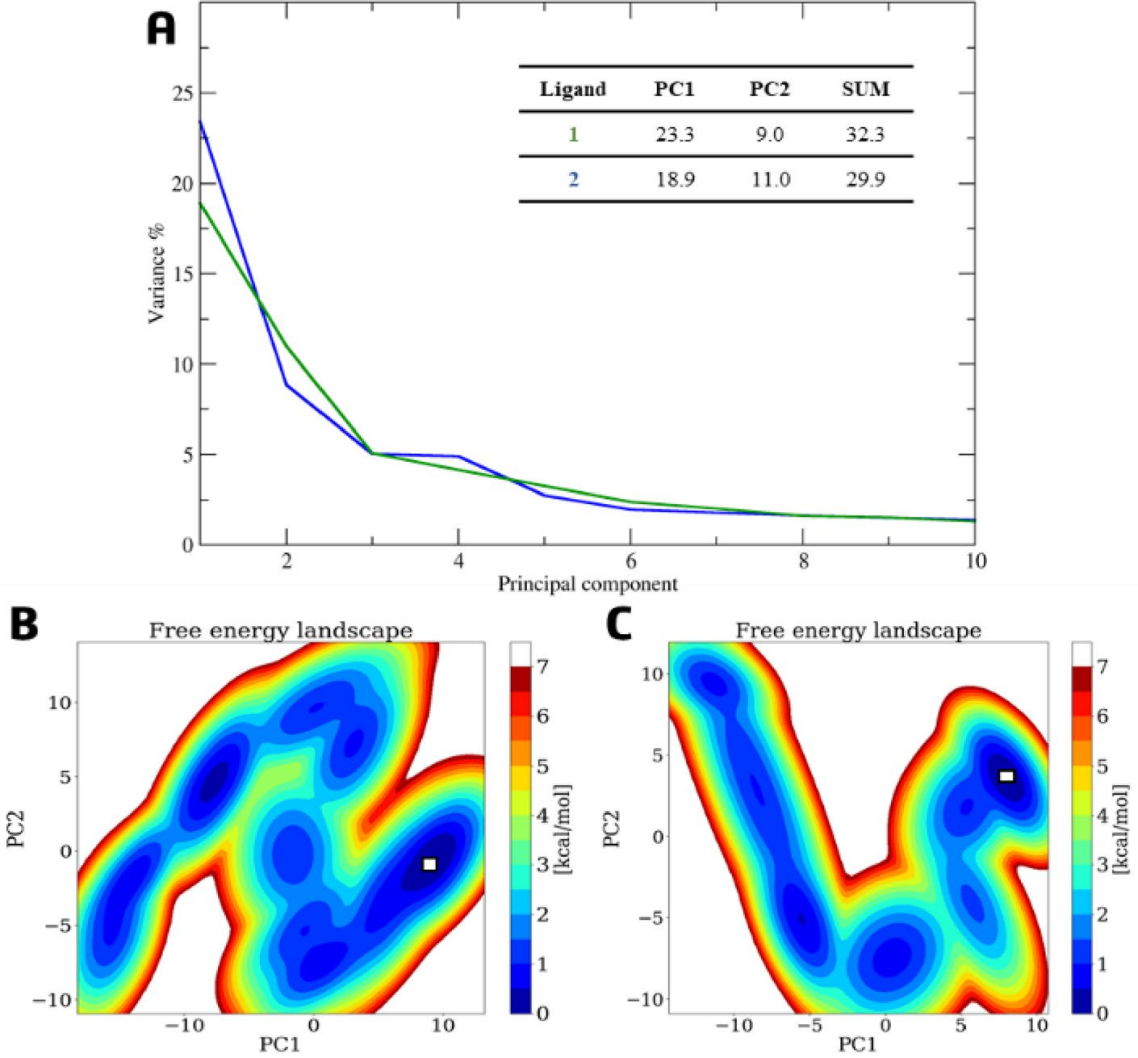
(**A**) Variance percentage plot depicting the eigenvectors of the first ten principal components (PC) for ligand **1** (blue line) and ligand **2** (green line); a table offers detailed values for PC1 and PC2, including their sums. The free energy landscape (FEL) map, generated from the first two principal components (PC1 and PC2) using the InfleCS program, highlights the lowest-energy (blue) and highest-energy (red) conformations for ligand **1** (**B**) and ligand **2** (**C**).

Given that both ligands remained stable only in cavity #1, we focused subsequent analyses on these trajectories. Principal component analysis (PCA) of the first two eigenvectors (PC1 and PC2) indicated that both systems explored a diverse conformational space rather than being dominated by a single motion^74^. The variance captured by PC1 and PC2 was 23% and 9% for ligand 1, and 18% and 11% for ligand **2**, respectively (Fig. 13A). The corresponding free energy landscape (FEL) maps show multiple energy minima (blue regions), with two predominant low-energy wells (dark blue), one of which corresponds to the most populated conformational cluster (Figs. 13B–C). The cumulative variance captured by the first ten principal components (PC1–PC10) was up to 53% for ligand **1** and up to 51% for ligand 2 (Table S20, Supplementary Information). It is noteworthy that regions exhibiting high RMSF, predominantly in loops, may be associated with the system’s flexibility, and thus reflect low variance^74^.

**Fig. 14:**
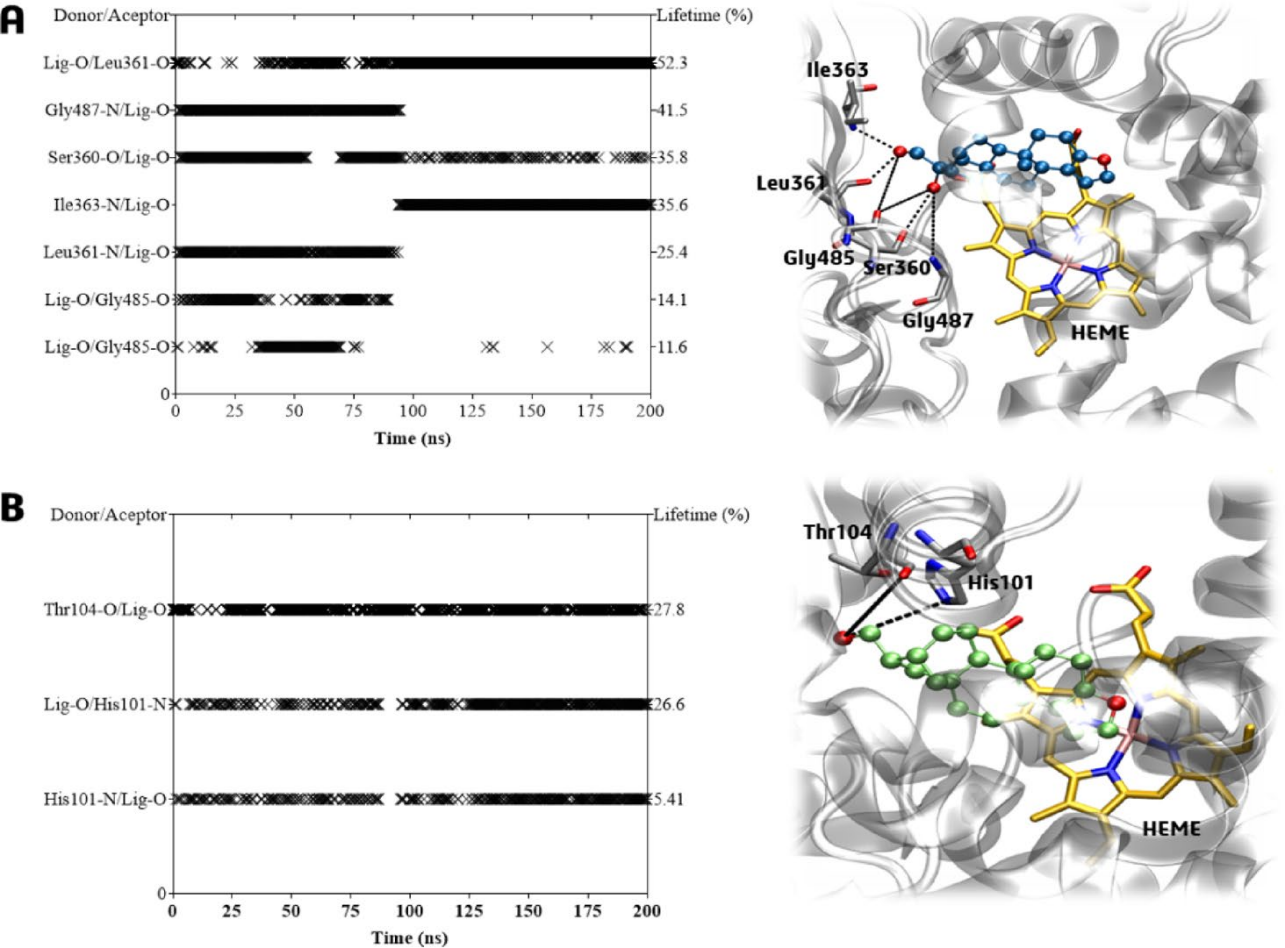
Hydrogen bonds and lifetime (%) plot, and the representative frame to CYP7A1-ligands complexes, to 200 ns of molecular dynamics simulations within cavity #1 of (**A**) Ligand **1** (ball and sticks in blue) and (**B**) Ligand **2** (ball and sticks in green). Grey sticks represent residues that form H-bonds (dashed black lines) with the ligands.

Hydrogen bond analysis revealed that ligand **1** maintained persistent interactions in cavity #1, with occupancies ranging from 11% to 52% (Fig. 14A). These interactions, which involved catalytic residues Leu361 (52.3% and 14.1%), for two distinct interactions, Gly487 (41.5%), Ser360 (35.8%), Ile363 (35.6%), and Gly485 (11.6%), consistent with the binding mode of the competitive inhibitor 7KCh near the heme group (Fig. 14A). In contrast, the dehydration in derivative **2** altered its interaction pattern. Although it occupied the same pocket, it shifted its position, losing aforementioned H-bonds and forming only two transient H-bonds with His101 and Thr104 (both with ~ 27% occupancy) (Fig. 14B).

Despite the different interaction patterns, the binding free energy (ΔG_bind_) calculations showed a similar affinity for both ligands, with ΔG_bind_ ≈ − 19 kcal/mol (Table 4). A detailed analysis of the energy components revealed that cafestol (**1**) had a more favorable electrostatic contribution (ΔE_elec_ = − 17.2 ± 1.8 kcal/mol) compared to 15,16-dehydrocafestol (**2**) (ΔE_elec_ = − 4.80 ± 1.7 kcal/mol). However, this advantage was counterbalanced by a higher polar solvation penalty for ligand **1** (ΔE_solv_ = + 34.7 ± 2.9 kcal/mol) compared to ligand **2** (ΔE_solv_ = + 21.6 ± 3.1 kcal/mol).

**Table 4:** The entropy (− TΔS) and binding free energy (ΔG_bind_) terms ± SD obtained from the MM-PBSA calculations of ligands **1** and **2** in complex with CYP7A1 (in kcal/mol). Van der Waals (ΔE_vdW_), electrostatic (ΔE_elec_), solvation (ΔE_solv_), and solvent-accessible surface area (ΔE_SASA_).

Ligand	−TΔS	ΔE_vdW_	ΔE_elec_	ΔE_solv_	ΔE_SASA_	ΔG_bind_
1	+ 1.70 ± 0.1	−39.4 ± 1.7	−17.2 ± 1.8	+ 34.7 ± 2.9	−3.77 ± 0.1	−19.4 ± 2.9^a^
2	+ 2.69 ± 0.6	−40.7 ± 1.9	−4.80 ± 1.7	+ 21.6 ± 3.1	−3.78 ± 0.1	−19.2 ± 3.3^b^

Different letters indicate statistical differences according to the Scott-Knott test.

**Fig. 15:**
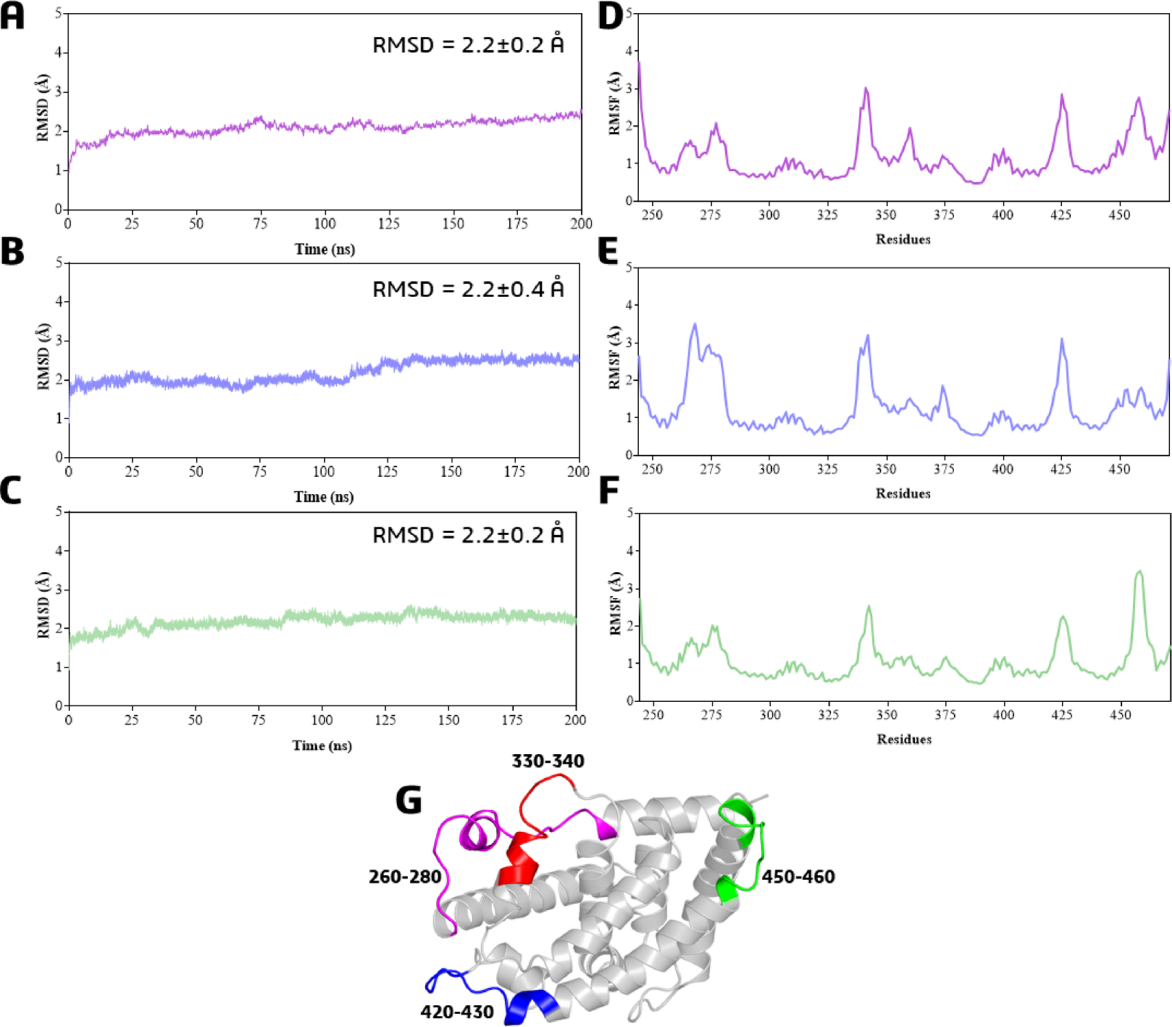
RMSD analysis of Cα atoms in the FXR-ligand protein complexes from molecular dynamics simulations (200 ns). (**A**) Holo enzyme form; (**B**) Ligand 1; (**C**) Ligand 2. RMSF analysis of Cα atoms in these complexes (**D**) Holo enzyme form; (**E**) Ligand 1; (**F**) Ligand 2; (**G**) 3D structure of FXR highlighting residues with fluctuations ≥ 2 Å.

Similarly, for the FXR receptor, MDS were focused on the orthosteric site (cavity #1) with both ligands **1** and **2**. When comparing the Cα-atom RMSD values between the holoenzyme and both ligands, we observed minimal variation, remaining within 2Å. This indicates that the complexes with ligands **1** and **2** are stable (see Figs. 15A–C and Table S21 for statistical data). Furthermore, the RMSF analysis showed that the most flexible regions were loops, specifically residues 330–340, 420–430, and 450–460 (Figs. 15D–G). Notably, the complex with ligand 1 induced increased flexibility in the 260–280 residue range (Figs. 15E, G and Supplementary Information for statistical data).

**Fig. 16:**
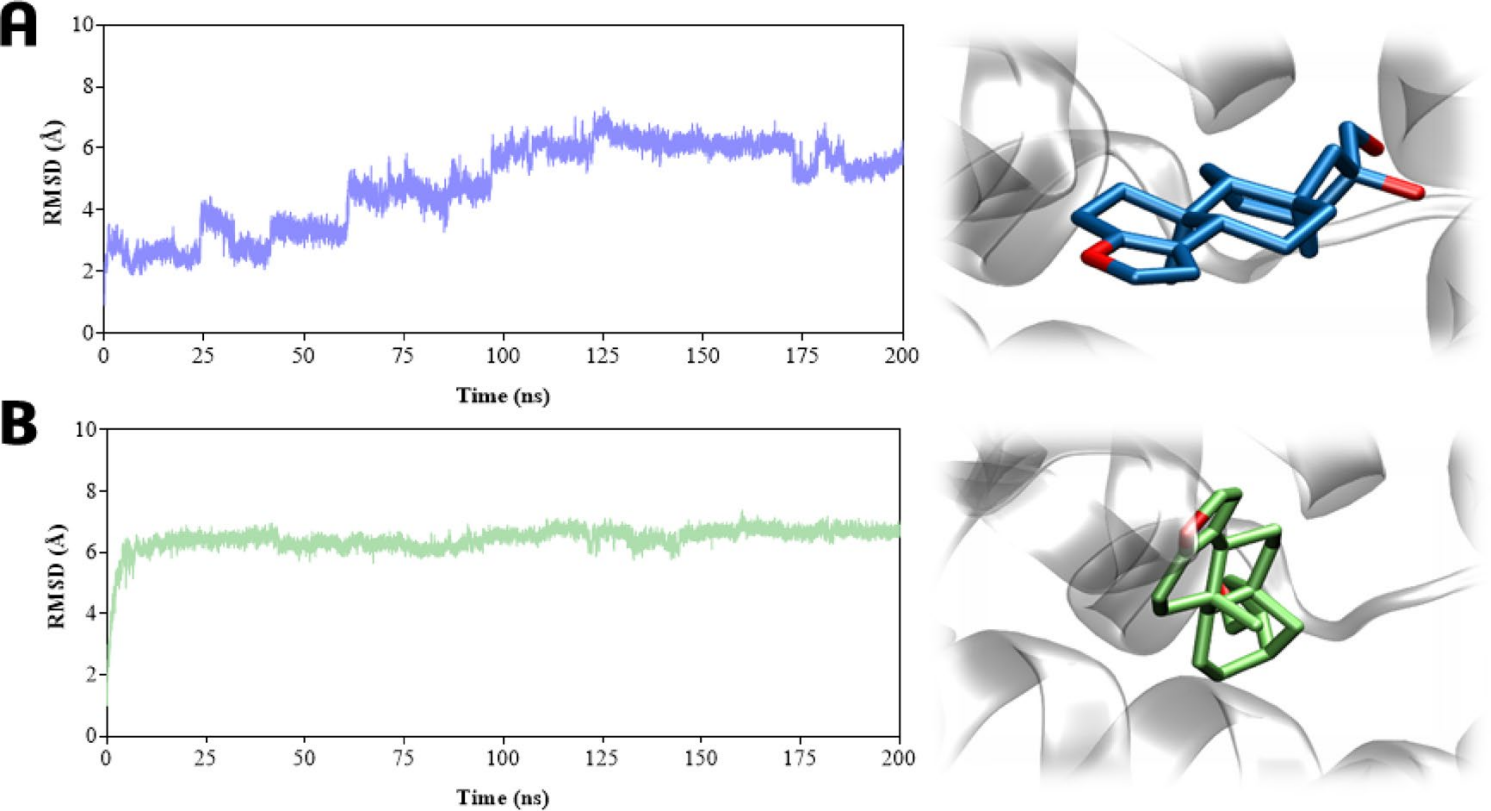
RMSD analysis of ligands atoms in molecular dynamics simulations (200 ns) on the FXR enzyme: (**A**) Ligand **1** (blue); (**B**) Ligand **2** (green).

The ligand RMSD values were 4.79 ± 2.5 Å for ligand **1** and 6.42 ± 0.6 Å for ligand **2**, confirming that both ligands maintained a stable binding mode within the orthosteric site throughout the simulation (Fig. 16 and Table S21 for statistical data). The lower RMSD for ligand **1** suggests a more consistent binding pose, while the slightly higher value for ligand **2** indicates greater conformational flexibility or variation in its binding pose.

**Fig. 17:**
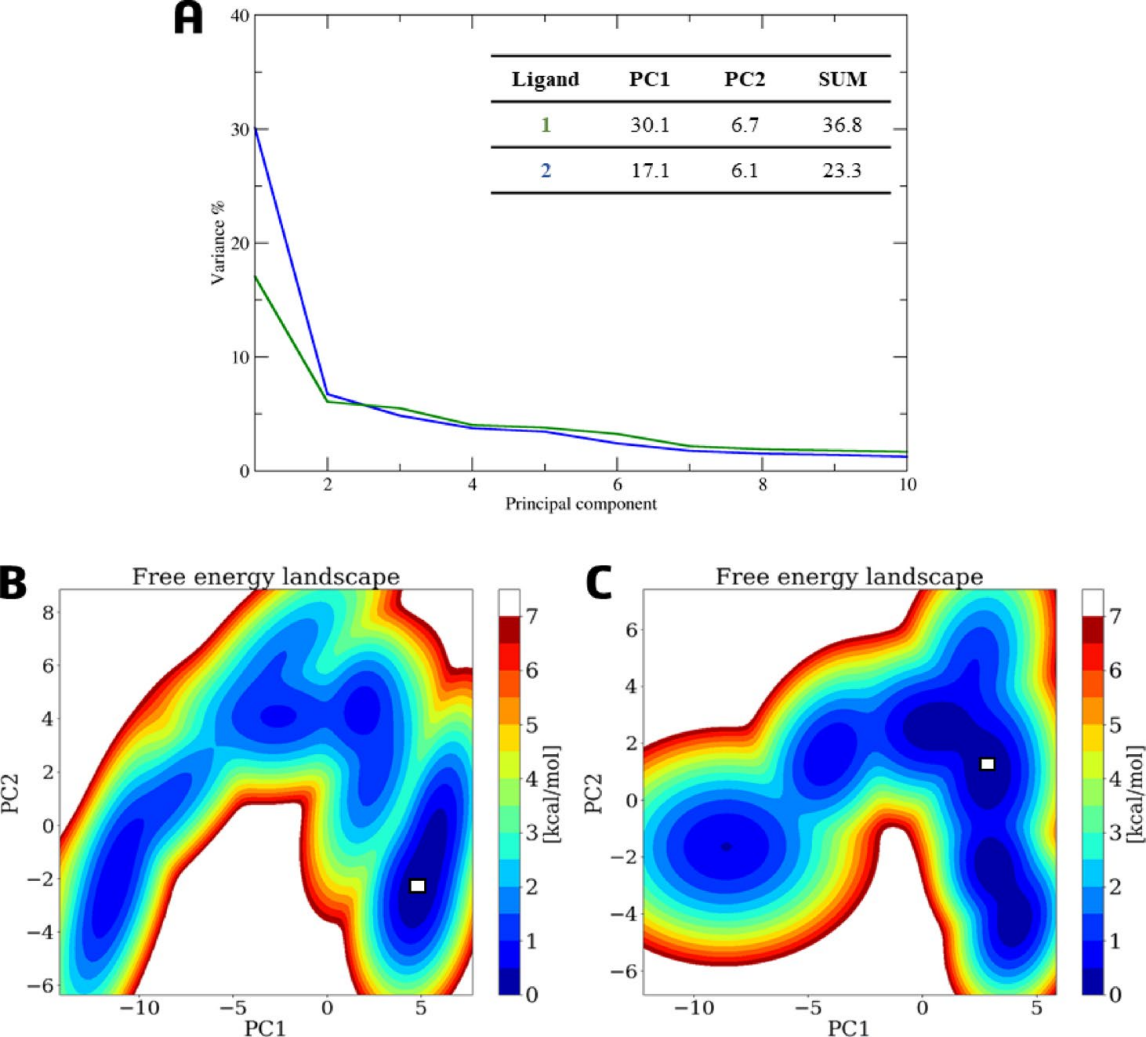
(**A**) Variance percentage plot depicting the eigenvectors of the first ten principal components (PC) for ligand **1** (blue line) and ligand **2** (green line); a table offers detailed values for PC1 and PC2, including their sums. The free energy landscape (FEL) map, generated from the first two principal components (PC1 and PC2) using the InfleCS program, highlights the lowest-energy (blue) and highest-energy (red) conformations for ligand **1** (**B**) and ligand **2** (**C**).

PCA of FXR complexes showed that ligand **1** induced a more defined conformational state, with PC1 accounting for 30% of the total variance and PC2 for 7%. In contrast, the complex with ligand **2** exhibited a broader conformational spectrum, with PC1 and PC2 accounting for 17% and 6% of the motion, respectively (Fig. 17A).

Furthermore, the FEL map for ligand **1** shows a single low-energy well (dark blue), corresponding to the most populated cluster (Fig. 17B), whereas ligand **2** explores multiple minima (Fig. 17C). The cumulative variance for PC1–PC10 was 57% for ligand **1** and 47% for ligand 2 (Table S22, Supplementary Information). In addition to CYP7A1, the FXR has regions with high RMSF, predominantly in loops, which may be associated with the system’s flexibility and thus reflect low variance^75^.

**Fig. 18:**
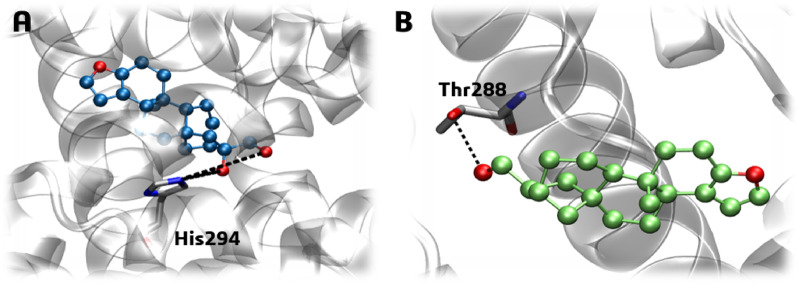
Hydrogen bonds of the representative frame to FXR-ligand complexes, to 200 ns of molecular dynamics simulations: (**A**) Ligand **1** (ball and sticks in blue) and (**B**) Ligand **2** (ball and sticks in green). Grey sticks represent residues that form H-bonds (dashed black lines).

Ligand **1** formed a single transient H-bond with His294 (24.9% occupancy) (Fig. 18A), while ligand **2** interacted with Thr288 (19% to 39.6% occupancy) (Fig. 18B). Despite non-persistent H-bonds, the ligands demonstrated favorable free binding energies, likely mainly due to hydrophobic interactions with the FXR binding pocket (Figs. 18A–B).

The calculated ΔG_bind_ values were − 25.8 ± 3.0 kcal/mol for ligand 1 and − 20.8 ± 3.1 kcal/mol for ligand **2**; the difference was not statistically significant, suggesting similar affinities (Table 5). Ligand **1** presented more favorable van der Waals (ΔEvdW = − 42.1 ± 2.1 kcal/mol) and electrostatic (ΔEele = − 11.9 ± 2.5 kcal/mol) contributions compared to ligand 2 (ΔEvdW = − 36.6 ± 2.3 kcal/mol; ΔEele = − 8.69 ± 2.1 kcal/mol). Although ligand **1** incurred a greater penalty for polar solvation, its stronger enthalpic interactions yielded a similar overall binding free energy. The lower RMSD, higher PC1 variance, and more defined FEL for ligand **1** collectively suggest that it adopts a more rigid and stable conformation within the FXR binding pocket.

**Table 5:** The entropy (− TΔS) and binding free energy (ΔG_bind_) terms ± SD obtained from the MM-PBSA calculations of ligands **1** and **2** in complex with FXR (in kcal/mol).

Ligand	−TΔS	ΔE_vdW_	ΔE_elec_	ΔE_solv_	ΔE_SASA_	ΔG_bind_
1	+ 3.45 ± 0.5	**−**42.1 ± 2.1	**−**11.9 ± 2.5	+ 28.9 ± 2.1	**−**3.66 ± 0.1	**−**25.8 ± 3.0^a^
2	+ 2.84 ± 0.6	**−**36.6 ± 2.3	**−**8.69 ± 2.1	+ 21.8 ± 2.5	**−**3.77 ± 0.1	**−**20.8 ± 3.1^a^

Van der Waals (ΔE_vdW_), electrostatic (ΔE_elec_), solvation (ΔE_solv_), and solvent-accessible surface area (ΔE_SASA_). Different letters indicate statistical differences according to the Scott-Knott test.

In summary, the molecular dynamics results reinforce the initial findings from molecular docking. For CYP7A1, both ligands were stable only in the orthosteric site (cavity #1), where they exhibited similar binding affinities (ΔG_bind_ ≈ − 19 kcal/mol), consistent with the docking scores. For FXR, both ligands also stayed stably bound in the orthosteric site, with ΔG_bind_ being statistically significant same.

### Pharmacophore model

**Fig. 19:**
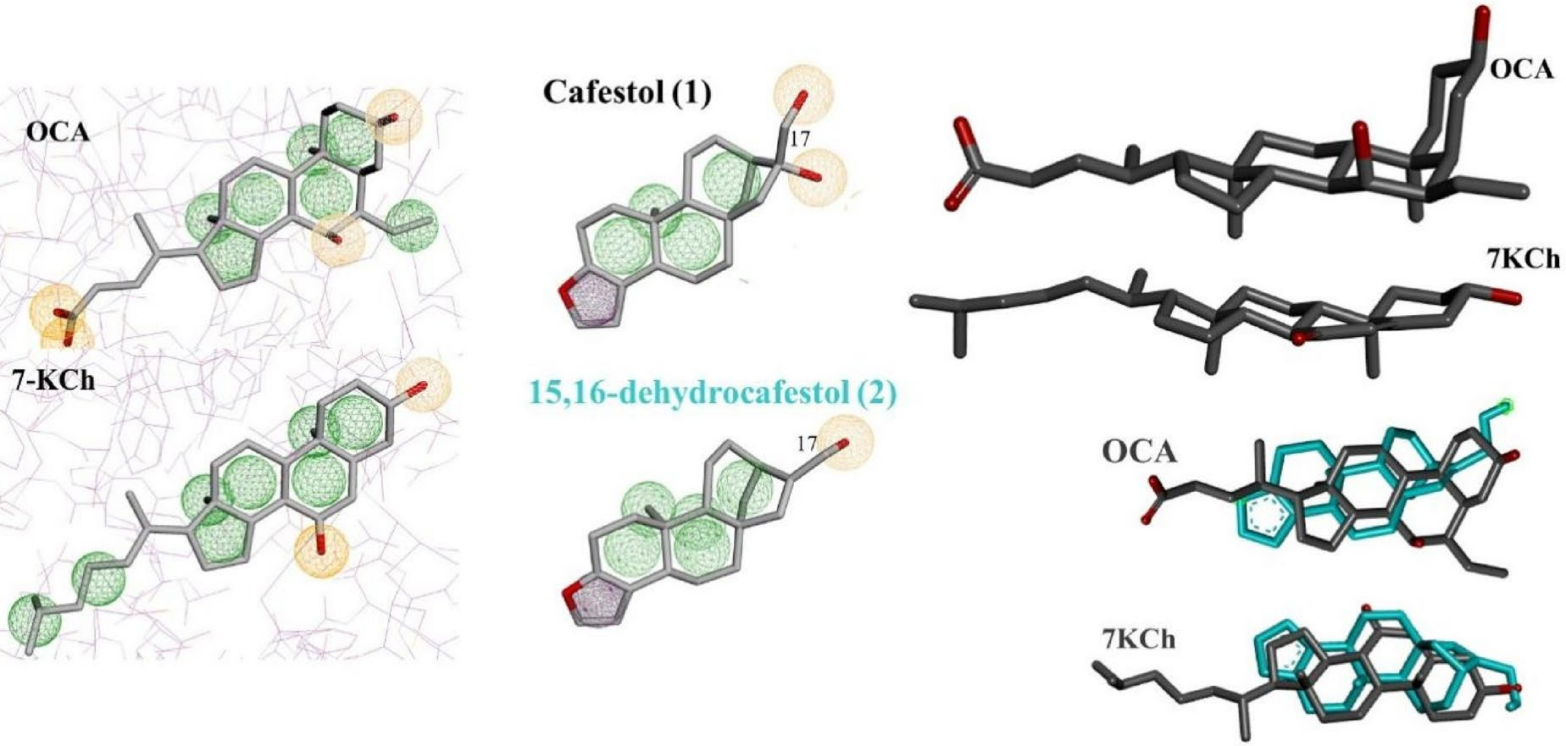
Structures of the diterpenes *ent-*kauranes cafestol (**1**) and 15,16-dehydrocafestol (**2**), obeticholic acid (OCA) and 7-ketocholesterol (7KCh), with the respective pharmacophore features: hydrophobic (green), hydrogen donor/acceptor (orange) and aromatic (purple).

To highlight the structural characteristics that allow interaction of *ent*-kaurane ligands with FXR and CYP7A1, the mapping of pharmacophore groups was performed (Fig. 19). Features of the pharmacophore profile for both targets are provided in Table S23 (Supplementary Information). In the ligands, hydrophobic regions stand out, characteristic of steroidal and *ent*-kaurane-type polycyclic systems, which provide conformational rigidity.

Although cafestol and its derivatives have a furan ring, binding modes for FXR have shown that it acts as a hydrophobic or aromatic region, whose function is variable according to the group of substituents of the ligand. The focus on the furan ring is due to the aromatic system being essential for the Nrf2-dependent anticarcinogenic and antioxidant activity of cafestol, two relevant activities for the diterpenoid^20,75^,. Therefore, evaluating the role of the furan ring in FXR agonism and CYP7A1 inhibition activities contributes to expanding knowledge of the structure-activity relationship (SAR) of cafestol.

By rotating the structure of OCA and 7KCh, it is possible to superimpose the steroidal nucleus with the *ent*-kaurane skeleton, in which the furan ring coincides with the steroid D-ring (cyclopentane) (Fig. 19). The possibility of superimposition was supported by the poses of 15,16-dehydrocafestol (**2**), the main cafestol roasting derivative, obtained by docking (Fig. 19).

Hydrogen bond acceptor and donor regions were the second most important structural attributes for the ligands, represented by the hydroxyl group. Hydroxyl group at C3 in OCA and 7KCh coincides spatially with the same group at C17 in cafestol and some analogues (Fig. 19) Therefore, this simple structure-activity relationship approach contributes to the explanation of similar binding modes between cafestol derivatives and reference ligands in the receptor (FXR) and enzyme (CYP7A1) binding sites.

### ADME profile of cafestol roasting derivatives

Table 6 summarizes some of the properties calculated on the SwissADME server for the cafestol main roasting derivatives **2** (15,16-dehydrocafestol) and **3** (cafestal). By comparing the calculated physicochemical properties, it is observed that modifications of the side chains have a minimal impact on solubility, with no great variation in bioaccessibility and bioavailability (Table 6). Both derivatives presented lower TPSA (topological polar surface area) values (**2**, 33.37 Å²; and **3**, 30.21 Å²) and higher consensus Log*P*_o/w_ (logarithm of the 1-octanol/water partition coefficient) values (**2**, 3.88; and **3**, 4.09) than cafestol (TPSA = 53.60 Å² and consensus Log*P*_o/w_ = 3.25). The identical bioavailability radar (Figures S6–S8, Supplementary Information) for the roasting derivatives shows great similarity to that described by Rendón-Rodríguez et al.^61^ for cafestol, differing only by reduction of the polar region and size due to the loss of hydroxyl groups. Like cafestol, **2** is predicted to be a substrate for the P-gp (permeability glycoprotein) efflux pump, while **3** is predicted to be a non-substrate. Unlike cafestol, both derivatives (**2** and **3**) are predicted to be inhibitors of CYP1A2 and CYP2C9.

**Table 6:** Predicted ADME properties for the main roasting products of Cafestol in the SwissADME server.

Physicochemical	Cafestol (1)	15,16-Dehydrocafestol (2)	Cafestal (3)
Formula	C_20_H_28_O_3_	C_20_H_26_O_2_	C_20_H_26_O_2_
Molecular weight (g/mol)	316.43	298.42	298.42
No. heavy atoms	23	22	22
No. aromatic heavy atoms	5	5	5
Fraction C*sp*^3^	0.80	0.70	0.75
No. rotatable bonds	1	1	1
No. H-bond acceptors	3	2	2
No. H-bond donors	2	1	0
TPSA (Å²)	53.60	33.37	30.21
Lipophilicity			
LogP_o/w_ (iLOGP)	2.76	3.06	2.94
Log*P*_o/w_ (XLOGP3)	3.28	4.04	4.60
Log*P*_o/w_ (WLOGP)	3.64	4.44	4.73
Log*P*_o/w_ (MLOGP)	2.89	3.68	3.68
Log*P*_o/w_ (SILICOS-IT)	3.68	4.19	4.51
Consensus Log*P*_o/w_	3.25	3.88	4.09
Pharmacokinetics			
GI absorption	High	High	High
BBB permeant	Yes	Yes	Yes
P-gp substrate	Yes	Yes	No
CYP1A2 inhibitor	No	Yes	Yes
CYP2C19 inhibitor	No	No	No
CYP2C9 inhibitor	No	Yes	Yes
CYP2D6 inhibitor	Yes	Yes	Yes
CYP3A4 inhibitor	No	No	No

## Conclusions

Molecular docking demonstrated that roasting derivatives and some phase I metabolites of cafestol interact with FXR and CYP7A1 similarly to their steroidal reference ligands, an agonist and inhibitor, respectively. The structural similarity between the *ent*-kaurane skeleton and steroids reinforces the ligands’ affinity profiles for the target proteins. For complexes with CYP7A1, furan rings and substituents in the metabolites’ structures were determinants for the interaction with the heme cofactor. Molecular dynamics simulations validated the orthosteric site preference (cavity #1) determined by docking and provided affinity estimates (ΔG_bind_), which were remarkably similar or equal for both ligands in CYP7A1 and FXR, respectively. This study has limitations that prevent the proposal of ligand activity profiles, as it involves only computational simulations. These in silico data require biological validation to assess whether these structural diterpene analogs act as FXR agonists and CYP7A1 inhibitors, and to elucidate whether the modulation of these protein targets contributes to the increase in serum cholesterol already reported for this coffee diterpene. Despite the lack of experimental validation, these initial theoretical data highlight the importance of investigating adverse effects of cafestol derivatives, compounds whose bioactivity is poorly studied in literature. Future research will be fundamental to understanding the role of these compounds in the cardiovascular risk associated with excessive consumption of unfiltered coffee.

## Supplementary Information

Below is the link to the electronic supplementary material.

Supplementary Material 1

Supplementary Material 2

## Data Availability

All data generated or analyzed during this study are included in this published article (and its Supplementary Information file).

## References

[1] Silva, M. A. E., Brand, A. L. M., Novaes, F. J. M. & Rezende, C. M. Cafestol, Kahweol and their acylated derivatives: antitumor potential, pharmacokinetics, and chemopreventive profile. *Food Rev. Int.* **39** (9), 7048–7080. 10.1080/87559129.2022.2141776 (2023).

[2] Moeenfard, M. & Alves, A. New trends in coffee diterpenes research from technological to health aspects. *Food Res. Int.* **134**, 109207. 10.1016/j.foodres.2020.109207 (2020). 32517949 10.1016/j.foodres.2020.109207

[3] Ren, Y., Wang, C., Xu, J. & Wang, S. Cafestol and kahweol: A review on their bioactivities and Pharmacological properties. *Int. J. Mol. Sci.* **20** (17), 4238. 10.3390/ijms20174238 (2019). 31480213 10.3390/ijms20174238 PMC6747192

[4] Thelle, D. S., Arnesen, E. & Førde, O. H. The Tromsø heart study: does coffee Raise serum cholesterol? *N. Engl. J. Med.* **308** (24), 1454–1457. 10.1056/NEJM198306163082405 (1983). 6855815 10.1056/NEJM198306163082405

[5] Hao, W. R. et al. The association between Cafestol and cardiovascular diseases: A comprehensive review. *Medicina* **60** (6), 867. 10.3390/medicina60060867 (2024). 38929484 10.3390/medicina60060867 PMC11205330

[6] Boekschoten, M. V., Hooiveld, G. J. E. & J.The dyslipidemic effect of coffee diterpenes. in Coffee: Consumption and Health Implications (ed. Farah, A.), ch.26, 541–547 *R. Soc. Chem.* , 10.1039/9781788015028-00541 (2019).

[7] Novaes, F. J. M. et al. Cafestol and Kahweol. in Coffee and Human Health HealthChemistry and Mechanisms of Action (ed. Grosso, G.), vol.45, ch.5, 71–113 10.1039/9781839166853-00071 (2025).

[8] Ricketts, M. L. Does coffee Raise cholesterol? *Future Lipidol.* **2** (4), 373–377. 10.2217/17460875.2.4.373 (2007).

[9] Guercia, E. et al. On the cholesterol Raising effect of coffee diterpenes Cafestol and 16-O-methylcafestol: interaction with farnesoid X receptor. *Int. J. Mol. Sci.* **25** (11), 6096. 10.3390/ijms25116096 (2024). 38892285 10.3390/ijms25116096 PMC11173301

[10] Ricketts, M. L. et al. The cholesterol-raising factor from coffee beans, cafestol, as an agonist ligand for the farnesoid and pregnane X receptors. *Mol. Endocrinol.* **21** (7), 1603–1616. 10.1210/me.2007-0133 (2007). 17456796 10.1210/me.2007-0133

[11] Post, S. M. et al. Cafestol increases serum cholesterol levels in Apolipoprotein E*3-Leiden Transgenic mice by suppression of bile acid synthesis. *Arterioscler. Thromb. Vasc. Biol.* **20** (6), 1551–1556. 10.1161/01.atv.20.6.1551 (2000). 10845871 10.1161/01.atv.20.6.1551

[12] Miyazaki, T. et al. Novel FXR agonist nelumal A suppresses colitis and inflammation-related colorectal carcinogenesis. *Sci. Rep.* **11** (1), 492. 10.1038/s41598-020-79916-5 (2021). 33436792 10.1038/s41598-020-79916-5 PMC7804240

[13] Modica, S., Gadaleta, R. M. & Moschetta, A. Deciphering the nuclear bile acid receptor FXR paradigm. *Nucl. Recept. Signal.* **8**, e005. 10.1621/nrs.08005 (2010). 21383957 10.1621/nrs.08005 PMC3049226

[14] Miao, J. et al. Bile acid signaling pathways increase stability of small heterodimer partner (SHP) by inhibiting ubiquitin-proteasomal degradation. *Genes Dev.* **23** (8), 986–996. 10.1101/gad.1773909 (2009). 19390091 10.1101/gad.1773909 PMC2675865

[15] Brown, A. J., Sharpe, L. J., Rogers, M. J. & Oxysterols From physiological tuners to Pharmacological opportunities. *Br. J. Pharmacol.* **178** (16), 3089–3103. 10.1111/bph.15073 (2021). 32335907 10.1111/bph.15073

[16] Post, S. M., De Wit, E. C. M. & Princen, H. M. G. Cafestol, the cholesterol-raising factor in boiled coffee, suppresses bile acid synthesis by downregulation of cholesterol 7α-hydroxylase and sterol 27-hydroxylase in rat hepatocytes. *Arteriosclerosis Thrombosis Vascular Biol.* 17(11), 3064–3070 10.1161/01.atv.17.11.3064 (1997). 10.1161/01.atv.17.11.3064 9409294

[17] Berman, H. M. et al. The protein data bank. *Nucleic Acids Res.* **28** (1), 235–242. 10.1093/nar/28.1.235 (2000). 10592235 10.1093/nar/28.1.235 PMC102472

[18] Novaes, F. J., da Silva, M. A., Silva, D. C., Neto, A., Rezende, C. M. & F. R., & Extraction of diterpene-phytochemicals in Raw and roasted coffee beans and beverage preparations and their relationship. *Plants* **12** (8), 1580. 10.3390/plants12081580 (2023). 37111804 10.3390/plants12081580 PMC10145731

[19] Andriolo, C. V., Novaes, F. J. M., Pereira, H. M. G., Sardela, V. F. & Rezende, C. M. Metabolic study of Cafestol using in Silico approach, zebrafish water tank experiments and liquid chromatography high-resolution mass spectrometry analyses. *J. Chromatogr. B*. **1186**, 123028. 10.1016/j.jchromb.2021.123028 (2021). 10.1016/j.jchromb.2021.123028 34801941

[20] Lam, L. K. T., Sparnins, V. L. & Wattenberg, L. W. Effects of derivatives of Kahweol and Cafestol on the activity of glutathione S-transferase in mice. *J. Med. Chem.* **30** (8), 1399–1403. 10.1021/jm00391a022 (1987). 3612687 10.1021/jm00391a022

[21] Speer, K. & Kölling-Speer, I. The lipid fraction of the coffee bean. *Braz. J. Plant. Physiol.* **18** (1), 201–216. 10.1590/S1677-04202006000100014 (2006).

[22] Sridevi, V., Giridhar, P. & Ravishankar, G. A. Evaluation of roasting and brewing effect on antinutritional diterpenes-cafestol and Kahweol in coffee. *Global J. Med. Res.* **11** (5), 17–22 https://medicalresearchjournal.org/index.php/GJMR/article/view/91/65 (2011).

[23] Chaachouay, N. & Zidane, L. Plant-derived natural products: A source for drug discovery and development. *Drugs Drug Candidates*. **3** (1), 184–207. 10.3390/ddc3010011 (2024).

[24] Phansalkar, P. S., Zhang, Z., Verenich, S. & Gerk, P. M. Pharmacokinetics and Bioavailability Enhancement of Natural Products. In Natural Products for Cancer Chemoprevention (eds Pezzuto, J., & Vang, O.) ch.4, 109–141Springer, Cham, 10.1007/978-3-030-39855-2_4 (2020).

[25] Luca, S. V. et al. Bioactivity of dietary polyphenols: the role of metabolites. *Crit. Rev. Food Sci. Nutr.* **60** (1), 626–659. 10.1080/10408398.2018.1546669 (2020). 30614249 10.1080/10408398.2018.1546669

[26] Atanasov, A. G. et al. Discovery and resupply of Pharmacologically active plant-derived natural products: A review. *Biotechnol. Adv.* **33** (8), 1582–1614. 10.1016/j.biotechadv.2015.08.001 (2015). 26281720 10.1016/j.biotechadv.2015.08.001 PMC4748402

[27] Goldstone, J. V. et al. Identification and developmental expression of the full complement of cytochrome P450 genes in zebrafish. *BMC Genom.* **11**, 643. 10.1186/1471-2164-11-643 (2010). 10.1186/1471-2164-11-643 PMC3012610 21087487

[28] Singh, S., Bani Baker, Q. & Singh, D. B. Molecular docking and molecular dynamics simulation. in Bioinformatics: Methods and Applications (eds Singh, D. B., & Pathak, R. K.) ch.18, 291–304 Elsevier, 10.1016/B978-0-323-89775-4.00014-6 (2022).

[29] Wang, S., Xie, J., Pei, J. & Lai, L. C. P. : An integrated platform for comprehensive protein cavity detection and property analyses with user-friendly tools and cavity databases. *J. Mol. Biol.* 435(14), 16814110.1016/j.jmb.2023.168141 (2023). 10.1016/j.jmb.2023.168141 37356903

[30] UniProt Consortium UniProt: the universal protein knowledgebase in 2025. *Nucleic Acids Res.* **53(D1)**, D609–D617. 10.1093/nar/gkae1010 (2025). 39552041 10.1093/nar/gkae1010 PMC11701636

[31] Tian, S. Y., Chen, S. M., Pan, C. X. & Li, Y. F. X. R. Structures, biology, and drug development for NASH and fibrosis diseases. *Acta Pharmacol. Sin.* **43** (5), 1120–1132. 10.1038/s41401-021-00849-4 (2022). 35217809 10.1038/s41401-021-00849-4 PMC9061771

[32] Tempel, W. et al. Structural characterization of human cholesterol 7α-hydroxylase. *J. Lipid Res.* **55** (9), 1925–1932. 10.1194/jlr.M050765 (2014). 24927729 10.1194/jlr.M050765 PMC4617357

[33] Pellicciari, R. et al. 6α-Ethyl-chenodeoxycholic acid (6-ECDCA), a potent and selective FXR agonist endowed with anticholestatic activity. *J. Med. Chem.* **45** (17), 3569–3572. 10.1021/jm025529g (2002). 12166927 10.1021/jm025529g

[34] Mi, L. Z. et al. Structural basis for bile acid binding and activation of the nuclear receptor FXR. *Mol. Cell*. **11** (4), 1093–1100. 10.1016/s1097-2765(03)00112-6 (2003). 12718893 10.1016/s1097-2765(03)00112-6

[35] Groom, C. R., Bruno, I. J., Lightfoot, M. P. & Ward, S. C. The Cambridge structural database. *Acta. Crystallogr. Sect. B*. **72** (Pt 2), 171–179. 10.1107/S2052520616003954 (2016). 10.1107/S2052520616003954 PMC4822653 27048719

[36] Guercia, E., Berti, F., Navarini, L., Demitri, N. & Forzato, C. Isolation and characterization of major diterpenes from C. canephora roasted coffee oil. *Tetrahedron Asymmetry*. **27** (14–15), 649–656. 10.1016/j.tetasy.2016.06.008 (2016).

[37] Lam, S. D., Das, S., Sillitoe, I. & Orengo, C. An overview of comparative modelling and resources dedicated to large-scale modelling of genome sequences. *Acta Crystallogr. Sect. D Struct. Biol.* **73**(Pt 8), 628–640 10.1107/S2059798317008920 (2017). 10.1107/S2059798317008920 PMC5571743 28777078

[38] He, Q., Jiang, M., Wang, Y. & Zheng, T. Pharmacovigilance of obeticholic acid: an analysis of the food and drug administration adverse event reporting system database. *Br. J. Clin. Pharmacol.* **91** (12), 3445–3460. 10.1002/bcp.70186 (2025). 40776461 10.1002/bcp.70186

[39] Waterhouse, A. et al. SWISS-MODEL: homology modelling of protein structures and complexes. *Nucleic Acids Res.* **46** (W1), W296–W303. 10.1093/nar/gky427 (2018). 29788355 10.1093/nar/gky427 PMC6030848

[40] Guercia, E., Forzato, C., Navarini, L. & Berti, F. Interaction of coffee compounds with serum albumins. Part II: diterpenes. *Food Chem.* **199**, 502–508. 10.1016/j.foodchem.2015.12.051 (2016). 26776001 10.1016/j.foodchem.2015.12.051

[41] Macrae, C. F. et al. Mercury 4.0: from visualization to analysis, design and prediction. *J. Appl. Crystallogr. 53(Pt*. **1**, 226–235. 10.1107/S1600576719014092 (2020). 10.1107/S1600576719014092 PMC6998782 32047413

[42] Guedes, I. A., Krempser, E. & Dardenne, L. E. DockThor 2.0: a free web server for protein-ligand virtual screening. XIX SBQT–Simpósio Brasileiro de Química Teórica (2017). http://fig.if.usp.br/~sbqt/sites/default/files/440-resumo.pdf.

[43] Huey, R., Morris, G. M. & Forli, S. Using AutoDock 4 and AutoDock Vina with AutoDockTools: A Tutorial. In The Scripps Research Institute Molecular (2012). https://autodock.scripps.edu/faqs-help/tutorial/using-autodock-4-with-autodocktools/2012_ADTtut.pdf.

[44] McDermott, L. et al. A solution to the anti-Bredt olefin synthesis problem. *Science* **386** (6721), eadq3519. 10.1126/science.adq3519 (2024). 39480919 10.1126/science.adq3519 PMC12450110

[45] Ramírez, D. & Caballero, J. Is it reliable to take the molecular docking top scoring position as the best solution without considering available structural data? *Molecules* 23(5), 1038 10.3390/molecules23051038 (2018). 10.3390/molecules23051038 PMC6102569 29710787

[46] Abraham, M. J. et al. GROMACS: High performance molecular simulations through multi-level parallelism from laptops to supercomputers. *SoftwareX* 1–2, 19–25 10.1016/j.softx.2015.06.001 (2015).

[47] Huang, J. & MacKerell, A. D. CHARMM36 all-atom additive protein force field: validation based on comparison to NMR data. *J. Comput. Chem.* **34** (25), 2135–2145. 10.1002/jcc.23354 (2013). 23832629 10.1002/jcc.23354 PMC3800559

[48] Pimentel, R. D. P. et al. Statine-based peptidomimetics as SARS-CoV-2 papain-like protease inhibitors: in Silico and in vitro studies. *Sci. Rep.* **15** (1), 26319. 10.1038/s41598-025-11599-2 (2025). 40685447 10.1038/s41598-025-11599-2 PMC12277432

[49] Yu, W., He, X., Vanommeslaeghe, K. & MacKerell, A. D. Extension of the CHARMM general force field to sulfonyl-containing compounds and its utility in biomolecular simulations. *J. Comput. Chem.* **33** (31), 2451–2468. 10.1002/jcc.23067 (2012). 22821581 10.1002/jcc.23067 PMC3477297

[50] Turner, P. J. XMGRACE (v.5.1.19). Center for Coastal and Land-Margin Research, Oregon Graduate Institute of Science and Technology, Beaverton, OR https://plasma-gate.weizmann.ac.il/pub/grace/src/grace5/ (2005).

[51] Camargo, P. G. et al. In Silico evaluation of N-aryl-1,10-phenanthroline-2-amines as potential inhibitors of T. cruzi GP63 zinc-metalloprotease by Docking and molecular dynamics simulations. *Sci. Rep.* **15** (1), 6036. 10.1038/s41598-025-90088-y (2025). 39971997 10.1038/s41598-025-90088-y PMC11839977

[52] Ferrari, B. S., Lima, C. H. S. & Albuquerque, M. G. Development, validation and analysis of a human profurin 3D model using comparative modeling and molecular dynamics simulations. *J. Biomol. Struct. Dynamics*. **42** (10), 5428–5446. 10.1080/07391102.2023.2231546 (2024). 10.1080/07391102.2023.2231546 37449759

[53] Westerlund, A. M., Delemotte, L. & InfleCS Clustering free energy landscapes with Gaussian mixtures. *J. Chem. Theory Comput.* **15** (12), 6752–6759. 10.1021/acs.jctc.9b00454 (2019). 31647864 10.1021/acs.jctc.9b00454

[54] Westerlund, A. M., Harpole, T. J., Blau, C. & Delemotte, L. Inference of calmodulin’s Ca²⁺-dependent free energy landscapes via Gaussian mixture model validation. *J. Chem. Theory Comput.* **14** (1), 63–71. 10.1021/acs.jctc.7b00346 (2018). 29144736 10.1021/acs.jctc.7b00346

[55] Valdés-Tresanco, M. S., Valdés-Tresanco, M. E., Valiente, P. A., Moreno, E. & gmx_MMPBSA A new tool to perform end-state free energy calculations with GROMACS. *J. Chem. Theory Comput.* **17** (10), 6281–6291. 10.1021/acs.jctc.1c00645 (2021). 34586825 10.1021/acs.jctc.1c00645

[56] Humphrey, W., Dalke, A. & Schulten, K. V. M. D. Visual molecular dynamics. *J. Mol. Graph.* **14** (1), 33–38. 10.1016/0263-7855(96)00018-5 (1996). 8744570 10.1016/0263-7855(96)00018-5

[57] Ferreira, D. F. & SISVAR A computer analysis system to fixed effects split plot type designs. *Brazilian J. Biometrics*. **37** (4), 529–535. 10.28951/rbb.v37i4.450 (2019).

[58] Tantithamthavorn, C., McIntosh, S., Hassan, A. E. & Matsumoto, K. The impact of automated parameter optimization on defect prediction models. *IEEE Trans. Software Eng.* **45** (7), 683–711. 10.1109/TSE.2018.2794977 (2019).

[59] Sunseri, J. & Koes, D. R. Pharmit: interactive exploration of chemical space. *Nucleic Acids Res.* **44** (W1), W442–W448. 10.1093/nar/gkw287 (2016). 27095195 10.1093/nar/gkw287 PMC4987880

[60] Daina, A., Michielin, O. & Zoete, V. SwissADME: a free web tool to evaluate pharmacokinetics, drug-likeness and medicinal chemistry friendliness of small molecules. *Sci. Rep.* **7**, 42717. 10.1038/srep42717 (2017). 28256516 10.1038/srep42717 PMC5335600

[61] Rendón-Rodríguez, J. J., Lopera-Rodríguez, J. A., Sanabria-Chanaga, E. & Röthlisberger, S. Potential inhibitory action of Cafestol on apoptosis proteins: an in-silico study. *Coffee Sci.* **20** (2025), e202266. 10.25186/.v20i.2266 (2025).

[62] Chakravarty, D., Lee, M. & Porter, L. L. Proteins with alternative folds reveal blind spots in AlphaFold-based protein structure prediction. *Curr. Opin. Struct. Biol.* **90**, 102973. 10.1016/j.sbi.2024.102973 (2025). 39756261 10.1016/j.sbi.2024.102973 PMC11791787

[63] Yusuf, D., Davis, A. M., Kleywegt, G. J. & Schmitt, S. An alternative method for the evaluation of Docking performance: RSR vs RMSD. *J. Chem. Inf. Model.* **48** (7), 1411–1422. 10.1021/ci800084x (2008). 18598022 10.1021/ci800084x

[64] Guedes, I. A. et al. DockThor-VS: A free platform for receptor-ligand virtual screening. *J. Mol. Biol.* **436** (17), 168548. 10.1016/j.jmb.2024.168548 (2024). 39237203 10.1016/j.jmb.2024.168548

[65] Martins, V. C., Silva, M. A. E., Veiga Jr, V. F., Pereira, H. M. & Rezende, C. M. Ent-kaurane diterpenoids from coffea genus: an update of chemical diversity and biological aspects. *Molecules* **30** (1), 59. 10.3390/molecules30010059 (2024). 39795116 10.3390/molecules30010059 PMC11722336

[66] Di Matteo, S. et al. The FXR agonist obeticholic acid inhibits the cancerogenic potential of human cholangiocarcinoma. *PLoS One*. **14** (1). 10.1371/journal.pone.0210077 (2019). 10.1371/journal.pone.0210077 PMC6345424 30677052

[67] Jang, H., Han, N., Staatz, C. E., Kwak, J. H. & Baek, I. Effect on lipid profile and clinical outcomes of obeticholic acid for the treatment of primary biliary cholangitis and metabolic dysfunction-associated steatohepatitis: A systematic review and meta-analysis. *Clin. Res. Hepatol. Gastroenterol.* **47** (10), 102227. 10.1016/j.clinre.2023.102227 (2023). 37884091 10.1016/j.clinre.2023.102227

[68] Fang, Y., Hegazy, L., Finck, B. N. & Elgendy, B. Recent advances in the medicinal chemistry of farnesoid X receptor. *J. Med. Chem.* **64** (24), 17545–17571. 10.1021/acs.jmedchem.1c01017 (2021). 34889100 10.1021/acs.jmedchem.1c01017

[69] Beigneux, A., Hofmann, A. F., Young, S. G. & Human CYP7A1 deficiency: progress and enigmas. *J. Clin. Invest.* **110** (1), 29–31. 10.1172/JCI16076 (2002). 12093884 10.1172/JCI16076 PMC151039

[70] Breuer, O., Sudjana-Sugiaman, E., Eggertsen, G., Chiang, J. Y. L. & Björkhem, I. Cholesterol 7α‐hydroxylase is up‐regulated by the competitive inhibitor 7‐oxocholesterol in rat liver. *Eur. J. Biochem.* **215** (3), 705–710. 10.1111/j.1432-1033.1993.tb18082.x (1993). 8354276 10.1111/j.1432-1033.1993.tb18082.x

[71] Boekschoten, M. V. et al. Coffee oil consumption increases plasma levels of 7α-hydroxy-4- cholesten-3-one in humans. *J. Nutr.* **135** (4), 785–789. 10.1093/jn/135.4.785 (2005). 15795435 10.1093/jn/135.4.785

[72] Matsubara, T., Li, F. & Gonzalez, F. J. FXR signaling in the enterohepatic system. *Mol. Cell. Endocrinol.* **368** (1–2), 17–29. 10.1016/j.mce.2012.05.004 (2013). 22609541 10.1016/j.mce.2012.05.004 PMC3491147

[73] Morrison, A. & Elgendy, B. Tailoring FXR modulators for intestinal specificity: recent progress and insights. *Molecules* **29** (9). 10.3390/molecules29092022 (2024). 38731514 10.3390/molecules29092022 PMC11085346

[74] Rahimi, M., Taghdir, M. & Joozdani, F. A. Dynamozones are the most Obvious sign of the evolution of conformational dynamics in HIV 1 protease. *Sci. Rep.* **13** (1), 14179. 10.1038/s41598-023-40818-x (2023). 37648682 10.1038/s41598-023-40818-x PMC10469195

[75] Higgins, L. G., Cavin, C., Itoh, K., Yamamoto, M. & Hayes, J. D. Induction of cancer chemopreventive enzymes by coffee is mediated by transcription factor Nrf2. Evidence that the coffee-specific diterpenes Cafestol and Kahweol confer protection against acrolein. *Toxicol. Appl. Pharmcol.* **226** (3), 328–337. 10.1016/j.taap.2007.09.018 (2008). 10.1016/j.taap.2007.09.018 18028974

