# Supplementary material for: Cafestol-derivatives as potential FXR agonists and CYP7A1 inhibitors and their impact on hypercholesterolemia: an in silico study
Source: Sci Rep. 2026 Feb 3;16:7102. doi: 10.1038/s41598-026-37519-6 (PMC12920617; doi:10.1038/s41598-026-37519-6)

**Supplementary Information**

**Cafestol-derivatives as potential FXR agonists and CYP7A1 inhibitors
and their impact on hypercholesterolemia: an *in silico* study**

Maria Alice Esteves da SILVA¹^,^², Priscila Goes CAMARGO³, Camilo Henrique da Silva LIMA²,
Carlos Rangel RODRIGUES³, Magaly Girão ALBUQUERQUE², Claudia Moraes de REZENDE¹^,^*

¹ Universidade Federal do Rio de Janeiro, Instituto de Química, Programa de Pós-Graduação em Ciência de Alimentos (PPGCAL), Departamento de Química Orgânica, Laboratório de Análise de Aromas CEP 21941-909, Cidade Universitária, Rio de Janeiro, RJ, Brazil.

² Universidade Federal do Rio de Janeiro, Instituto de Química, Departamento de Química Orgânica, Laboratório de Modelagem Molecular (LabMMol), CEP 21941-909, Cidade Universitária, Rio de Janeiro, RJ, Brazil.

³ Universidade Federal do Rio de Janeiro, Departamento de Fármacos e Medicamentos, Faculdade de Farmácia, Laboratório de Modelagem Molecular e QSAR (ModMolQSAR), CEP 21941-170, Cidade Universitária, Rio de Janeiro, RJ, Brazil.

*crezende@iq.ufrj.br

**Table S1.** Smiles code of ligands references obeticholic acid (OCA) and 7-ketocholesterol (7KCh), cafestol (**1**) and its derivatives (**2**–**14**).

| # | SMILES |
| --- | --- |
| OCA^(a)^ | CC[C@@H]1[C@@H]2C[C@@H](CC[C@@]2([C@H]3CC[C@]4([C@H]([C@@H]3[C@@H]1O)CC[C@@H]4[C@H](C)CCC(=O)O)C)C)O |
| 7KCh^(b)^ | C[C@H](CCCC(C)C)[C@H]1CC[C@@H]2[C@@]1(CC[C@H]3[C@H]2C(=O)C=C4[C@@]3(CC[C@@H](C4)O)C)C |
| 1 | C[C@]12[C@@](CC[C@]3(CC4(O)CO)[C@@]2([H])CC[C@]4([H])C3)([H])C5=C(OC=C5)CC1 |
| 2 | C[C@]12[C@@](CC[C@]3(C=C4CO)[C@@]2([H])CC[C@]4([H])C3)([H])C5=C(OC=C5)CC1 |
| 3 | C[C@]12[C@@](CC[C@]3(CC4C([H])=O)[C@@]2([H])CC[C@]4([H])C3)([H])C5=C(OC=C5)CC1 |
| 4 | C[C@]12[C@@](CC[C@]3(C/C4=C([H])\O)[C@@]2([H])CC[C@]4([H])C3)([H)C5=C(OC=C5)CC1 |
| 5 | C[C@]12[C@@](CC[C@]3(C/C4=C(O)\[H])[C@@]2([H])CC[C@]4([H])C3)([H)C5=C(OC=C5)CC1 |
| 6 | C[C@]12[C@@](CC[C@]3(CC4(O)CO)[C@@]2([H])CC[C@]4([H])C3)([H])[C@]5([H])[C@](OCC5)([H])CC1 |
| 7 | C[C@@]1(C2)[C@@](CC[C@]3(CC4(O)CO)[C@@]1([H])CC[C@]4([H])C3)([HC5=C(OC=C5)C2=O |
| 8 | C[C@]12[C@@](C(C[C@]3(CC4(O)CO)[C@@]2([H])CC[C@]4([H])C3)=O)([H])C5=C(OC=C5)CC1 |
| 9 | C[C@]12[C@@]([C@H](O)C[C@]3(CC4(O)CO)[C@@]2([H])CC[C@]4(O)C3)([H])C5=C(OC=C5)CC1 |
| 10 | C[C@]12[C@@]([C@H](O)C[C@]3(CC4(O)CO)[C@@]2([H])CC[C@]4(O)C3)([H])C5=C(OC=C5)CC1 |
| 11 | C[C@]12[C@@]([C@@H](O)C[C@]3(CC4(O)CO)[C@@]2([H])CC[C@]4(O)C3)([H])C5=C(OC=C5)CC1 |
| 12 | C[C@]12[C@@]([C@@H](O)C[C@]3(CC4(O)CO)[C@@]2([H])CC[C@]4([H])C3)([H])C5=C(OC=C5)CC1 |
| 13 | C[C@]12[C@@]([C@@H](O)C[C@]3(CC4(O)CO)[C@@]2([H])CC[C@]4([H])C3)([H])C5=C(OC=C5)CC1 |
| 14 | C[C@]12[C@@]([C@@H](O)C[C@]3(CC4(O)CO)[C@@]2([H])CC[C@]4([H])C3)([H])C5=C(OC=C5)CC1 |
| ^(a)^ OCA: PDB ID: CHC; ^(b)^ 7KCh: PDB ID: 0GV | |

**Table S2.** Advanced parameters for cavity detection described in CavityPlus server.

| Parameter | Value |
| --- | --- |
| ERASER_BALL_RADIUS (Å) | 10 |
| SEPARATE_MIN_DEPTH | 8 |
| MIN_ABSTRACT_LIMIT (Å^3^) | 1500 × 0.125 |
| SEPARATE_MAX_LIMIT (Å^3^) | 6000 × 0.125 |
| MIN_ABSTRACT_DEPTH | 2 |
| MIN_CAVITY_VOLUME (Å^3^) | 100 × 0.125 |
| RANK_SCORE | 1.5 |

**Table S3.** Summary of cavity #1 properties of the farnesoid X receptor (FXR) obtained from CavityPlus server.

| Property | Value |
| --- | --- |
| Pred Max pK_d_ | 11.92 |
| Pred Ave pK_d_ | 6.84 |
| DrugScore | 2913.00 |
| Drugability | Strong |
| Surface Area (Å²) | 1136.25 |
| Center (Å) | 15.75; 40.75; 15.0 |
| Size (Å) | 24.5; 19.5; 15.5 |
| Volume (Å³) | 1503.50 |
| Residues | LEU-58-A, THR-146-A, ALA-93-A, LYS-22-A, ILE-95-A, ARG-91-A, LEU-47-A, LEU-108-A, ILE-122-A, ASN-53-A, ILE-112-A, ILE-29-A, PRO-26-A, LEU-211-A, ASN-21-A, LYS-32-A, VAL-85-A, PHE-96-A, THR-62-A, SER-105-A, GLU-109-A, VAL-55-A, GLN-23-A, MET-25-A, VAL-57-A, PRO-101-A, TRP-229-A, HIS-104-A, LYS-98-A, MET-88-A, PHE-89-A, ARG-24-A, SER-118-A, MET-210-A, THR-48-A, GLN-27-A, ILE-117-A, SER-19-A, PHE-61-A, GLU-94-A, ALA-87-A, LEU-225-A, ASN-31-A, GLN-56-A, TYR-20-A, ASN-43-A, TYR-142-A, PHE-44-A, ALA-51-A, GLU-49-A, VAL-59-A, ASP-106-A, HIS-54-A, GLU-60-A, LEU-90-A, ARG-111-A, PHE-126-A, GLN-139-A, HIS-207-A, TYR-121-A, MET-125-A, LYS-99-A, TRP-214-A, ILE-46-A, GLY-116-A, MET-50-A, SER-92-A, ILE-33-A, THR-30-A, ARG-113-A, PHE-221-A, ASN-97-A, SER-115-A, TYR-129-A, THR-123-A, THR-52-A |

**Table S4.** Summary of cavity #2 properties of the farnesoid X receptor (FXR) obtained from CavityPlus server.

| Property | Value |
| --- | --- |
| Pred Max pK_d_ | 7.53 |
| Pred Ave pK_d_ | 6.79 |
| DrugScore | −842.00 |
| Drugability | Weak |
| Surface Area (Å²) | 236.25 |
| Center (Å) | −1.0; 33.5; 20.5 |
| Size (Å) | 8.0; 10.0; 13.0 |
| Volume (Å³) | 750.38 |
| Residues | PRO-124-A, ILE-122-A, ARG-215-A, LEU-211-A, GLU-209-A, GLU-120-A, HIS-205-A, SER-118-A, ALA-208-A, MET-210-A, ASP-119-A, MET-212-A, ILE-117-A, HIS-206-A, HIS-207-A, TYR-121-A, MET-125-A, TRP-214-A, GLY-116-A, PHE-203-A, SER-213-A, THR-123-A |
| ID Score (CorrSite 1.0) | −0.17 |

**Table S5.** Summary of cavity #3 properties of the farnesoid X receptor (FXR) obtained from CavityPlus server.

| Property | Value |
| --- | --- |
| Pred Max pK_d_ | 8.39 |
| Pred Ave pK_d_ | 5.49 |
| DrugScore | −1292.00 |
| Drugability | Weak |
| Surface Area (Å²) | 137.75 |
| Center (Å) | 9.25; 24.5; 20.5 |
| Size (Å) | 11.0; 10.5; 7.5 |
| Volume (Å³) | 237.00 |
| Residues | LEU-211-A, GLU-209-A, VAL-85-A, TRP-229-A, HIS-205-A, GLU-227-A, ALA-208-A, CYS-226-A, SER-83-A, GLU-86-A, LEU-80-A, HIS-206-A, ALA-84-A, ARG-201-A, HIS-207-A, VAL-231-A, ASP-230-A, LYS-81-A, GLY-82-A, PHE-203-A, ASN-204-A, ILE-228-A, ARG-155-A |
| ID Score (CorrSite 1.0) | 1.66 |

**Table S6.** Summary of cavity #1 properties of the cholesterol-7-alpha-hydroxylase (CYP7A1) obtained from CavityPlus server.

| Property | Value |
| --- | --- |
| Pred Max pK_d_ | 10.52 |
| Pred Ave pK_d_ | 6.94 |
| DrugScore | 3524.00 |
| Drugability | Strong |
| Surface Area (Å²) | 1324.00 |
| Center (Å) | 12.25; 10.75; −41.25 |
| Size (Å) | 20.5; 20.5; 26.5 |
| Volume (Å³) | 1443.38 |
| Residues | ARG-364-A, PHE-102-A, ILE-114-A, THR-442-A, PHE-100-A, ILE-454-A, ALA-221-A, SER-358-A, GLY-446-A, GLY-440-A, THR-121-A, SER-360-A, LEU-280-A, ILE-350-A, ASN-124-A, GLY-43-A, LEU-263-A, TYR-41-A, LYS-277-A, SER-286-A, ARG-260-A, ASP-127-A, PRO-445-A, MET-72-A, ILE-291-A, PHE-270-A, GLY-438-A, ASP-115-A, LEU-42-A, PRO-391-A, LEU-141-A, HIS-101-A, ILE-488-A, PHE-109-A, ALA-293-A, GLU-453-A, ILE-187-A, ALA-218-A, VAL-220-A, THR-290-A, LEU-354-A, PRO-217-A, ALA-288-A, VAL-281-A, THR-278-A, GLU-123-A, PHE-216-A, CYS-444-A, VAL-282-A, LEU-71-A, SER-482-A, THR-294-A, GLU-274-A, ALA-106-A, ALA-108-A, ALA-285-A, SER-105-A, ASN-289-A, THR-132-A, GLY-487-A, ALA-484-A, LEU-361-A, LEU-133-A, ALA-103-A, LEU-448-A, TRP-284-A, SER-113-A, ASN-264-A, ARG-483-A, ALA-441-A, LEU-486-A, GLN-287-A, PHE-437-A, ILE-443-A, PHE-129-A, ALA-359-A, ASN-126-A, ASN-362-A, ILE-363-A, LEU-256-A, HIS-279-A, THR-104-A, THR-128-A, MET-435-A, SER-439-A, SER-268-A, PRO-436-A, LYS-98-A, ALA-450-A, ILE-130-A, ILE-451-A, LEU-283-A, TYR-434-A, CYS-44-A, PHE-449-A, ARG-447-A, GLY-485-A, TRP-97-A, PRO-40-A, HIS-111-A, LYS-99-A, ILE-125-A |

**Table S7.** Summary of cavity #2 properties of the cholesterol-7-alpha-hydroxylase (CYP7A1) obtained from CavityPlus server.

| Property | Value |
| --- | --- |
| Pred Max pK_d_ | 10.96 |
| Pred Ave pK_d_ | 6.97 |
| DrugScore | 97.00 |
| Drugability | Medium |
| Surface Area (Å²) | 525.25 |
| Center (Å) | 6.5; 15.75; −29.5 |
| Size (Å) | 13.0; 18.5; 15.0 |
| Volume (Å³) | 845.25 |
| Residues | GLN-481-A, PHE-209-A, SER-482-A, LEU-206-A , LYS-210-A, LYS-214-A, LEU-489-A, LEU-46-A, ILE-291-A, PHE-174-A, PRO-491-A, PHE-228-A, PHE-212-A, GLN-211-A, ASN-289-A, MET-227-A, GLN-47-A, PRO-292-A, LEU-492-A, TYR-176-A, SER-173-A, ASP-480-A, ALA-45-A, ILE-488-A, ILE-495-A, CYS-44-A, PRO-490-A, TRP-284-A, ASP-494-A, ALA-218-A, MET-171-A, PHE-295-A, THR-290-A, ARG-483-A, GLY-43-A, TYR-41-A, GLN-287-A, TYR-172-A, LEU-223-A, VAL-215-A, PRO-217-A, ALA-288-A, ASP-213-A, ASP-207-A, PHE-216-A, ASN-493-A, LEU-219-A |
| ID Score (CorrSite 1.0) | 0.88 |

**Table S8.** Summary of cavity #3 properties of the cholesterol-7-alpha-hydroxylase (CYP7A1) obtained from CavityPlus server.

| Property | Value |
| --- | --- |
| Pred Max pKd | 11.51 |
| Pred Ave pKd | 6.94 |
| DrugScore | 761.00 |
| Drugability | Strong |
| Surface Area (Å²) | 882.25 |
| Center (Å) | 1.0; 35.75; −42.75 |
| Size (Å) | 18.0; 17.5; 19.5 |
| Volume (Å³) | 1619.25 |
| Residues | ARG-194-A, ASP-191-A, LEU-206-A, LYS-210-A, ALA-165-A, GLU-329-A, LYS-500-A, LYS-162-A, ASN-148-A, LEU-203-A, ARG-250-A, PHE-174-A, ASP-196-A, ARG-151-A, ALA-164-A, LEU-192-A, MET-461-A, THR-197-A, PHE-501-A, ILE-152-A, LEU-149-A, TYR-499-A, THR-163-A, GLY-189-A, PRO-156-A, ARG-177-A, LEU-185-A, VAL-157-A, TYR-176-A, SER-173-A, VAL-178-A, PRO-332-A, GLU-181-A, GLU-147-A, GLY-330-A, VAL-167-A, PRO-155-A, PHE-180-A, PHE-465-A, LYS-199-A, MET-153-A, TYR-464-A, ILE-202-A, SER-158-A, GLN-150-A, LEU-328-A, ARG-154-A, GLU-169-A, ARG-190-A, ARG-195-A, ALA-200-A, GLN-198-A, ASN-331-A, MET-179-A, MET-146-A |
| ID Score (CorrSite 1.0) | 1.87 |

**Table S9.** Features of the best poses for cafestol (**1**) and its derivatives (**2**–**14**). Data determined through docking simulations in the predicted cavity #1 of the farnesoid X receptor (FXR) using the DockThor server. Obethicolic acid (OCA). Not determined (n.d.).

| FXR (Cavity #1)^(a)^ | | | | | |
| --- | --- | --- | --- | --- | --- |
| # | **Score** | **E_Total_** | **E_Internal_** | **E_vdW_** | **E_Elec_** |
| OCA | −11.309 | −1.678 | −53.699 | −37.989 | −15.710 |
| 1 | −10.062 | 37.620 | −31.761 | −30.848 | −0.913 |
| 2 | −9.835 | 5.841 | −31.050 | −30.529 | −0.521 |
| 3 | −9.874 | 7.706 | −31.182 | −30.110 | −1.072 |
| 4 | n.d. | n.d. | n.d. | n.d. | n.d. |
| 5 | −10.095 | 6.322 | −31.387 | −29.840 | −1.547 |
| 6 | −9.731 | 81.998 | −36.360 | −30.970 | −5.390 |
| 7 | −9.989 | 19.911 | −31.574 | −30.462 | −1.112 |
| 8 | −9.957 | 21.999 | −31.955 | −30.688 | −1.267 |
| 9 | −10.132 | 19.944 | −32.973 | −30.168 | −2.805 |
| 10 | −10.086 | 19.311 | −33.675 | −29.096 | −4.579 |
| 11 | −10.124 | 17.678 | −32.711 | −28.531 | −4.180 |
| 12 | −10.131 | 18.253 | −32.572 | −30.178 | −2.394 |
| 13 | −10.063 | 11.643 | −32.527 | −28.430 | −4.097 |
| 14 | −10.132 | 17.189 | −31.902 | −30.893 | −1.009 |
| ^(a)^ E_Total_ (Total Energy, kcal/mol); E_Internal_ (Internal Energy, kcal/mol); E_vdW_ (van der Waals Energy, kcal/mol); E_Elec_ (Electrostatic Energy, kcal/mol) | | | | | |

**Table S10.** Features of the best poses for cafestol (**1**) and its derivatives (**2**–**14**). Data determined through docking simulations in the predicted cavity #2 of the farnesoid X receptor (FXR) using the DockThor server. Not determined (n.d.).

| FXR (Cavity #2)^(a)^ | | | | | |
| --- | --- | --- | --- | --- | --- |
| # | **Score** | **E_Total_** | **E_Internal_** | **E_vdW_** | **E_Elec_** |
| 1 | −8.303 | 19.549 | −26.137 | −18.804 | −7.333 |
| 2 | −7.759 | 11.528 | −20.263 | −15.795 | −4.468 |
| 3 | −8.027 | 25.955 | −19.610 | −17.883 | −1.727 |
| 4 | n.d. | n.d. | n.d. | n.d. | n.d. |
| 5 | −8.479 | 22.973 | −20.563 | −19.134 | −1.429 |
| 6 | −7.589 | 17.998 | −23.133 | −14.356 | −8.777 |
| 7 | −7.896 | 32.284 | −26.975 | −11.811 | −15.164 |
| 8 | −7.989 | 28.556 | −25.496 | −11.088 | −14.408 |
| 9 | −7.964 | 25.099 | −27.918 | −10.787 | −17.131 |
| 10 | n.d. | n.d. | n.d. | n.d. | n.d. |
| 11 | n.d. | n.d. | n.d. | n.d. | n.d. |
| 12 | −7.000 | 20.497 | −31.595 | 4.422 | −36.017 |
| 13 | −7.176 | 23.854 | −28.797 | −4.775 | −24.022 |
| 14 | −6.984 | 19.534 | −31.377 | 4.855 | −36.232 |
| ^(a)^ E_Total_ (Total Energy, kcal/mol); E_Internal_ (Internal Energy, kcal/mol); E_vdW_ (van der Waals Energy, kcal/mol); E_Elec_ (Electrostatic Energy, kcal/mol) | | | | | |

**Table S11.** Features of the best poses for cafestol (**1**) and its derivatives (**2–14**). Data determined through docking simulations in the predicted cavity #3 of the farnesoid X receptor (FXR) using the DockThor server. Not determined (n.d.).

| FXR (Cavity #3)^(a)^ | | | | | |
| --- | --- | --- | --- | --- | --- |
| # | **Score** | **E_Total_** | **E_Internal_** | **E_vdW_** | **E_Elec_** |
| 1 | −8.311 | 76.667 | 22.595 | 21.604 | 0.991 |
| 2 | −8.274 | 45.711 | 13.747 | 13.016 | 0.731 |
| 3 | −8.280 | 49.879 | 10.085 | 10.293 | −0.208 |
| 4 | n.d. | n.d. | n.d. | n.d. | n.d. |
| 5 | −7.419 | 13.264 | −25.648 | −12.346 | −13.302 |
| 6 | −7.332 | 101.118 | 54.680 | 64.101 | −9.421 |
| 7 | n.d. | n.d. | n.d. | n.d. | n.d. |
| 8 | n.d. | n.d. | n.d. | n.d. | n.d. |
| 9 | n.d. | n.d. | n.d. | n.d. | n.d. |
| 10 | n.d. | n.d. | n.d. | n.d. | n.d. |
| 11 | n.d. | n.d. | n.d. | n.d. | n.d. |
| 12 | n.d. | n.d. | n.d. | n.d. | n.d. |
| 13 | n.d. | n.d. | n.d. | n.d. | n.d. |
| 14 | n.d. | n.d. | n.d. | n.d. | n.d. |
| ^(a)^ E_Total_ (Total Energy, kcal/mol); E_Internal_ (Internal Energy, kcal/mol); E_vdW_ (van der Waals Energy, kcal/mol); E_Elec_ (Electrostatic Energy, kcal/mol) | | | | | |

**Table S12.** Features of the best poses for cafestol (**1**) and its derivatives (**2–14**). Data determined through docking simulations in the predicted cavity #1 of the farnesoid X receptor (FXR) using AutoDock program. Obethicolic acid (OCA).

| FXR (Cavity #1)^(a)^ | | | | | | |
| --- | --- | --- | --- | --- | --- | --- |
| # | **E_Bind_** | **K_i_** | **CR** | **E_Intermol_** | **E_Internal_** | **E_Elec_** |
| OCA | −12.93 | 0.000335 | 1 | −14.42 | −0.62 | −0.57 |
| 1 | −9.16 | 0.914 | 1 | −10.05 | −1.37 | −0.12 |
| 2 | −9.22 | 0.173 | 1 | −9.82 | −0.06 | −0.04 |
| 3 | −9.23 | 0.171 | 1 | −9.53 | −0.14 | −0.04 |
| 4 | −9.01 | 0.246 | 1 | −9.31 | +0.04 | −0.13 |
| 5 | −9.29 | 0.155 | 2 | −9.59 | +0.04 | −0.14 |
| 6 | −9.39 | 0.131 | 1 | −10.28 | −1.29 | −0.10 |
| 7 | −9.22 | 0.175 | 1 | −10.11 | −0.82 | −0.02 |
| 8 | −9.24 | 0.169 | 1 | −10.13 | −1.36 | −0.11 |
| 9 | −8.21 | 0.95 | 1 | −9.70 | −9.59 | −0.11 |
| 10 | −8.44 | 0.0651 | 1 | −9.93 | −1.33 | −0.12 |
| 11 | −8.95 | 0.005 | 1 | −9.23 | −0.10 | −0.02 |
| 12 | −8.05 | 1.26 | 3 | −9.54 | −1.38 | −0.05 |
| 13 | −8.08 | 1.19 | 2 | −9.28 | −1.40 | −0.08 |
| 14 | −8.26 | 0.879 | 2 | −9.45 | −1.41 | −0.14 |
| ^(a)^ E_Bind_ (Binding Energy, kcal/mol); K_i_ (Estimated K_i_, µM); CR (Cluster Rank); E_Intermol_ (Final Intermolecular Energy, kcal/mol); E_Internal_ (Final Total Internal Molecular Energy, kcal/mol); E_Elec_ (Electrostatic Energy, kcal/mol) | | | | | | |

**Table S13.** Features of the best poses for cafestol (**1**) and its derivatives (**2**–**14**). Data determined through docking simulations in the predicted cavity #2 of the farnesoid X receptor (FXR) using AutoDock program.

| FXR (Cavity #2)^(a)^ | | | | | | |
| --- | --- | --- | --- | --- | --- | --- |
| # | **E_Bind_** | **K_i_** | **CR** | **E_Intermol_** | **E_Internal_** | **E_Elec_** |
| 1 | −5.57 | 82.69 | 1 | −5.87 | −0.08 | −0.06 |
| 2 | −5.48 | 95.54 | 1 | −6.08 | −0.07 | −0.06 |
| 3 | −5.58 | 82.60 | 1 | −5.83 | −0.06 | −0.04 |
| 4 | −6.32 | 23.22 | 1 | −6.62 | +0.07 | −0.26 |
| 5 | −5.60 | 78.01 | 1 | −6.50 | −1.30 | −0.32 |
| 6 | −6.16 | 30.38 | 1 | −6.97 | −1.27 | −0.09 |
| 7 | −6.66 | 13.04 | 1 | −7.56 | −1.34 | −0.10 |
| 8 | −6.40 | 20.19 | 1 | −7.30 | −1.36 | −0.09 |
| 9 | −5.40 | 110.92 | 1 | −6.89 | −0.10 | −0.26 |
| 10 | −6.25 | 26.16 | 1 | −6.55 | −0.09 | +0.01 |
| 11 | −6.38 | 21.00 | 1 | −7.84 | −1.31 | −0.22 |
| 12 | −5.70 | 66.75 | 1 | −7.19 | −0.14 | −0.18 |
| 13 | −6.25 | 26.08 | 1 | −7.45 | −1.40 | −0.15 |
| 14 | −6.07 | 35.68 | 1 | −7.26 | −1.41 | −0.09 |
| ^(a)^ E_Bind_ (Binding Energy, kcal/mol); K_i_ (Estimated K_i_, µM); CR (Cluster Rank); E_Intermol_ (Final Intermolecular Energy, kcal/mol); E_Internal_ (Final Total Internal Molecular Energy, kcal/mol); E_Elec_ (Electrostatic Energy, kcal/mol) | | | | | | |

**Table S14.** Features of the best poses for cafestol (**1**) and its derivatives (**2**–**14**). Data determined through docking simulations in the predicted cavity #3 of the farnesoid X receptor (FXR) using AutoDock program.

| FXR (Cavity #3)^(a)^ | | | | | | |
| --- | --- | --- | --- | --- | --- | --- |
| # | **E_Bind_** | **K_i_** | **CR** | **E_Intermol_** | **E_Internal_** | **E_Elec_** |
| 1 | −4.30 | 707.19 | 1 | −5.19 | 1.40 | −0.13 |
| 2 | −5.11 | 179.15 | 1 | −5.71 | −0.04 | −0.13 |
| 3 | −5.30 | 133 | 1 | −5.59 | −0.08 | 0.00 |
| 4 | −5.34 | 121.84 | 1 | −5.64 | +0.04 | −0.08 |
| 5 | −5.29 | 140.73 | 1 | −5.42 | +0.06 | −0.15 |
| 6 | −4.97 | 226.80 | 1 | −5.87 | −1.30 | −0.16 |
| 7 | −6.44 | 19.04 | 1 | −7.33 | −1.44 | −0.14 |
| 8 | −6.92 | 8.42 | 1 | −7.82 | −0.77 | −0.12 |
| 9 | −6.72 | 11.94 | 1 | −8.21 | −1.29 | −0.25 |
| 10 | −6.17 | 30.14 | 1 | −7.66 | −1.06 | −0.27 |
| 11 | −6.26 | 25.75 | 1 | −7.57 | −1.12 | −0.19 |
| 12 | −6.46 | 18.48 | 2 | −7.95 | −1.38 | −0.21 |
| 13 | −7.06 | 6.69 | 2 | −8.25 | −1.36 | −0.32 |
| 14 | −6.22 | 27.80 | 2 | −7.41 | −1.40 | −0.20 |
| ^(a)^ E_Bind_ (Binding Energy, kcal/mol); K_i_ (Estimated K_i_, µM); CR (Cluster Rank); E_Intermol_ (Final Intermolecular Energy, kcal/mol); E_Internal_ (Final Total Internal Molecular Energy, kcal/mol); E_Elec_ (Electrostatic Energy, kcal/mol) | | | | | | |

**Table S15.** Features of the best poses for cafestol (**1**) and its derivatives (**2**–**14**). Data determined through docking simulations in the predicted cavity #1 of the cholesterol−7−alpha−hydroxylase (CYP7A1) using AutoDock program. 7−ketocholesterol (7KCh).

| CYP7A1 (Cavity #1)^(a)^ | | | | | | |
| --- | --- | --- | --- | --- | --- | --- |
| # | **E_Bind_** | **K_i_** | **CR** | **E_Intermol_** | **E_Internal_** | **E_Elec_** |
| 7KCh | −13.98 | 0.0000567 | 1 | −15.47 | +0.00 | +0.01 |
| 1 | −9.63 | 0.087 | 1 | −10.52 | −1.30 | −0.15 |
| 2 | −9.82 | 0.063 | 2 | −10.41 | −0.06 | −0.05 |
| 3 | −9.85 | 0.06 | 2 | −10.15 | −0.10 | −0.15 |
| 4 | −9.86 | 0.06 | 1 | −10.16 | +0.04 | −0.08 |
| 5 | −9.72 | 0.075 | 1 | −10.02 | +0.04 | −0.11 |
| 6 | −9.24 | 0.0167 | 1 | −10.14 | −1.28 | −0.20 |
| 7 | −9.78 | 0.0677 | 1 | −10.67 | −1.17 | −0.20 |
| 8 | −9.72 | 0.0744 | 1 | −10.62 | −1.28 | −0.18 |
| 9 | −8.80 | 0.354 | 1 | −10.29 | −1.25 | −0.12 |
| 10 | −8.25 | 0.894 | 1 | −9.74 | −1.24 | −0.17 |
| 11 | −8.29 | 0.842 | 1 | −9.69 | −1.20 | −0.14 |
| 12 | −8.33 | 0.780 | 1 | −9.82 | −1.25 | −0.20 |
| 13 | −8.42 | 0.124 | 1 | −9.61 | −1.20 | −0.17 |
| 14 | −8.46 | 0.629 | 1 | −9.65 | −1.36 | −0.13 |
| ^(a)^ E_Bind_ (Binding Energy, kcal/mol); K_i_ (Estimated K_i_, µM); CR (Cluster Rank); E_Intermol_ (Final Intermolecular Energy, kcal/mol); E_Internal_ (Final Total Internal Molecular Energy, kcal/mol); E_Elec_ (Electrostatic Energy, kcal/mol) | | | | | | |

**Table S16.** Features of the best poses for cafestol (**1**) and its derivatives (**2**–**14**). Data determined through docking simulations in the predicted cavity #2 of the cholesterol−7−alpha−hydroxylase (CYP7A1) using AutoDock program.

| CYP7A1 (Cavity #2)^(a)^ | | | | | | |
| --- | --- | --- | --- | --- | --- | --- |
| # | **E_Bind_** | **K_i_** | **CR** | **E_Intermol_** | **E_Internal_** | **E_Elec_** |
| 1 | −6.95 | 8.00 | 1 | −7.85 | −1.33 | −0.38 |
| 2 | −6.81 | 10.22 | 1 | −7.40 | −0.03 | −0.30 |
| 3 | −7.32 | 4.28 | 1 | −7.62 | −0.07 | −0.11 |
| 4 | −7.10 | 6.21 | 1 | −7.40 | +0.06 | −0.40 |
| 5 | −6.93 | 8.37 | 1 | −7.22 | +0.04 | −0.16 |
| 6 | −7.94 | 1.52 | 1 | −8.83 | −1.30 | −0.27 |
| 7 | −6.88 | 9.00 | 1 | −7.78 | −1.34 | −0.38 |
| 8 | −6.89 | 8.94 | 1 | −7.78 | −1.34 | −0.43 |
| 9 | −5.96 | 42.44 | 1 | −7.46 | −0.69 | −0.53 |
| 10 | −6.38 | 21.00 | 1 | −7.87 | −1.29 | −0.42 |
| 11 | −6.79 | 10.49 | 1 | −8.28 | −1.15 | −0.26 |
| 12 | −6.75 | 11.27 | 1 | −8.24 | −1.37 | −0.22 |
| 13 | −6.64 | 13.66 | 1 | −7.52 | −0.09 | −0.31 |
| 14 | −6.54 | 15.96 | 1 | −7.74 | −1.43 | −0.09 |
| ^(a)^ E_Bind_ (Binding Energy, kcal/mol); K_i_ (Estimated K_i_, µM); CR (Cluster Rank); E_Intermol_ (Final Intermolecular Energy, kcal/mol); E_Internal_ (Final Total Internal Molecular Energy, kcal/mol); E_Elec_ (Electrostatic Energy, kcal/mol) | | | | | | |

**Table S17.** Features of the best poses for cafestol (**1**) and its derivatives (**2–14**). Data determined through docking simulations in the predicted cavity #3 of the cholesterol−7−alpha−hydroxylase (CYP7A1) using AutoDock program.

| CYP7A1 (Cavity #3) | | | | | | |
| --- | --- | --- | --- | --- | --- | --- |
| # | **E_Bind_** | **K_i_** | **CR** | **E_Intermol_** | **E_Internal_** | **E_Elec_** |
| 1 | −7.04 | 6.92 | 1 | −7.93 | −1.38 | +0.01 |
| 2 | −7.28 | 4.58 | 1 | −7.88 | −0.06 | −0.08 |
| 3 | −6.40 | 20.39 | 1 | −6.53 | −0.15 | −0.17 |
| 4 | −6.58 | 15.01 | 1 | −6.57 | +0.07 | −0.31 |
| 5 | −7.51 | 3.13 | 1 | −7.81 | +0.04 | −0.09 |
| 6 | −6.33 | 22.90 | 1 | −7.23 | −1.34 | −0.06 |
| 7 | −6.92 | 8.47 | 1 | −7.81 | −1.34 | −0.47 |
| 8 | −6.85 | 9.57 | 1 | −7.74 | −1.35 | −0.37 |
| 9 | −6.65 | 13.38 | 1 | −8.14 | −1.66 | −0.27 |
| 10 | −7.58 | 2.79 | 1 | −7.88 | −0.10 | −0.41 |
| 11 | −5.84 | 51.99 | 1 | −7.00 | −1.04 | −0.34 |
| 12 | −6.58 | 15.14 | 1 | −8.07 | −1.34 | −0.36 |
| 13 | −6.38 | 21.12 | 3 | −7.57 | −1.36 | −0.31 |
| 14 | −6.36 | 21.65 | 1 | −7.56 | −1.39 | −0.37 |
| ^(a)^ E_Bind_ (Binding Energy, kcal/mol); K_i_ (Estimated K_i_, µM); CR (Cluster Rank); E_Intermol_ (Final Intermolecular Energy, kcal/mol); E_Internal_ (Final Total Internal Molecular Energy, kcal/mol); E_Elec_ (Electrostatic Energy, kcal/mol) | | | | | | |

**Table S18.** Statistical data on RMSD values from the triplicate molecular dynamics simulations of CYP7A1 Cα atoms in the presence of ligand **1** and ligand **2** on cavities #1–3. Different letters indicate a statistically significant difference according to the Scott-Knott test.

| **Cavity** | **Ligand 1 (Å)** | **Ligand 2 (Å)** |
| --- | --- | --- |
| **1** | 2.2 ± 0.4^a^ | 2.3 ± 0.3^a^ |
| **2** | 2.1 ± 0.1^a^ | 2.2 ± 0.2^a^ |
| **3** | 2.2 ± 0.4^a^ | 2.1 ± 0.4^a^ |

**Table S19.** Statistical data on RMSD values from the triplicate molecular dynamics simulations of ligand **1** and ligand **2** in cavities #1–3 on CYP7A1. Different letters indicate a statistically significant difference according to the Scott-Knott test.

| **Cavity** | **Ligand 1 (Å)** | **Ligand 2 (Å)** |
| --- | --- | --- |
| **1** | 3.0 ± 1.0^a^ | 2.7 ± 2.0^a^ |
| **2** | 17.2 ± 0.7^b^ | 32.9 ± 12^b^ |
| **3** | 11.4 ± 5.0^b^ | 24.4 ± 14^b^ |

**Table S20.** The PC1 to PC10 cumulative variance of ligands **1** and **2** in the complex within CYP7A1 on cavity #1.

| **PC** | **Ligand 1 (%)** | **Ligand 2 (%)** |
| --- | --- | --- |
| **1** | 23.3 | 18.9 |
| **2** | 32.2 | 29.9 |
| **3** | 37.2 | 34.9 |
| **4** | 42.1 | 39.1 |
| **5** | 44.9 | 42.3 |
| **6** | 46.9 | 44.7 |
| **7** | 48.7 | 46.7 |
| **8** | 50.3 | 48.3 |
| **9** | 51.8 | 49.8 |
| **10** | 53.2 | 51.1 |

**Table S21.** Statistical data on RMSD values from the triplicate molecular dynamics simulations of FXR Cα atoms and ligand atoms FXR-ligand **1** and ligand **2** complexes. Different letters indicate a statistically significant difference according to the Scott-Knott test.

| **Complex** | **Ligand atoms (Å)** | **Cα protein atoms(Å)** |
| --- | --- | --- |
| **1** | 4.79 ± 2.5 ^a^ | 2.2 ± 0.2^b^ |
| **2** | 6.42 ± 0.6 ^a^ | 2.2 ± 0.4^b^ |

**Table S22.** The PC1 to PC10 cumulative variance of ligands **1** and **2** in the complex within FXR on cavity #1.

| **PC** | **Ligand 1 (%)** | **Ligand 2 (%)** |
| --- | --- | --- |
| **1** | 30.1 | 17.1 |
| **2** | 36.8 | 23.1 |
| **3** | 41.7 | 28.6 |
| **4** | 45.4 | 32.6 |
| **5** | 48.9 | 36.4 |
| **6** | 51.3 | 39.7 |
| **7** | 53.0 | 41.8 |
| **8** | 54.6 | 43.8 |
| **9** | 55.9 | 45.5 |
| **10** | 57.2 | 47.2 |

**Table S23.** Pharmacophore profile associated to cavity #1 of farnesoid X receptor (FXR) and cholesterol-7-alpha-hydroxylase (CYP7A1). Data obtained from CavPharmer (CavityPlus).

|  | FXR | | CYP7A1 | |
| --- | --- | --- | --- | --- |
| Feature Type^(a)^ | **Number (ligand mode)** | **Number (no ligand mode)** | **Number (ligand mode)** | **Number (no ligand mode)** |
| HBD | 2 | 8 | 1 | 7 |
| HBA | 4 | 6 | 1 | 4 |
| Positive | 0 | 0 | 0 | 0 |
| Negative | 0 | 2 | 0 | 1 |
| Hydrophobic | 10 | 6 | 10 | 5 |
| ^(a)^ HBD (H-Bond Donor Center); HBA (H-Bond Acceptor Center); Positive (Positive Electrostatic Center); Negative (Negative Electrostatic Center); Hydrophobic (Hydrophobic Center) | | | | |

**Figure S1.** Models of farnesoid receptor X (FXR) and cholesterol-7-alpha-hydroxylase (CYP7A1) in complex with obeticholic acid (OCA) and 7-ketocholesterol (7KCh), respectively. The models are available in ModelArchive (modelarchive.org) with the accession codes ma−japwz (FXR model) and ma−qyq0l (CYP7A1 model).

| FXR model (Ligand: obeticholic acid, OCA) | CYP7A1 model (Ligand: 7-ketocholesterol, 7KCh) |
| --- | --- |
| 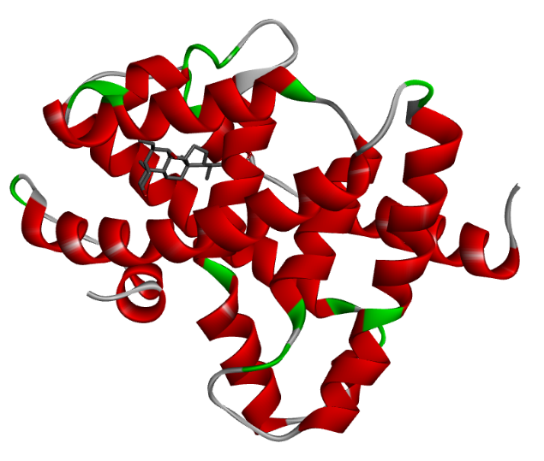 | **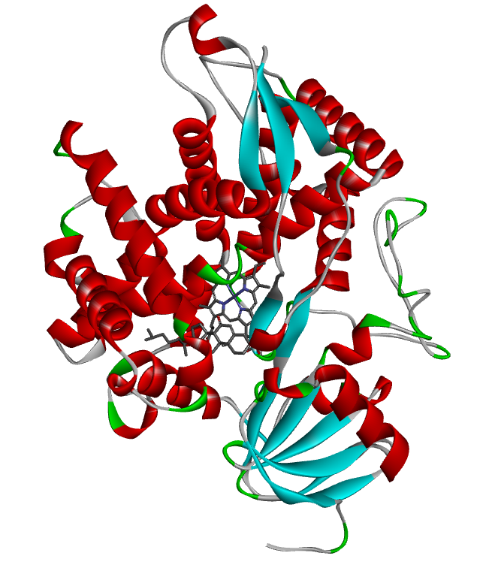** |

**Figure S2.** Overlays of the models obtained by comparative modeling in red (FXR) and green (CYP7A1) with the corresponding models predicted by AlphaFold2 in cyan (FXR) and purple (CYP7A1). The OCA (FXR) and 7KCh (CYP7A1) ligands and the heme cofactor are derived solely from the comparative modeling models.

| **FXR** | **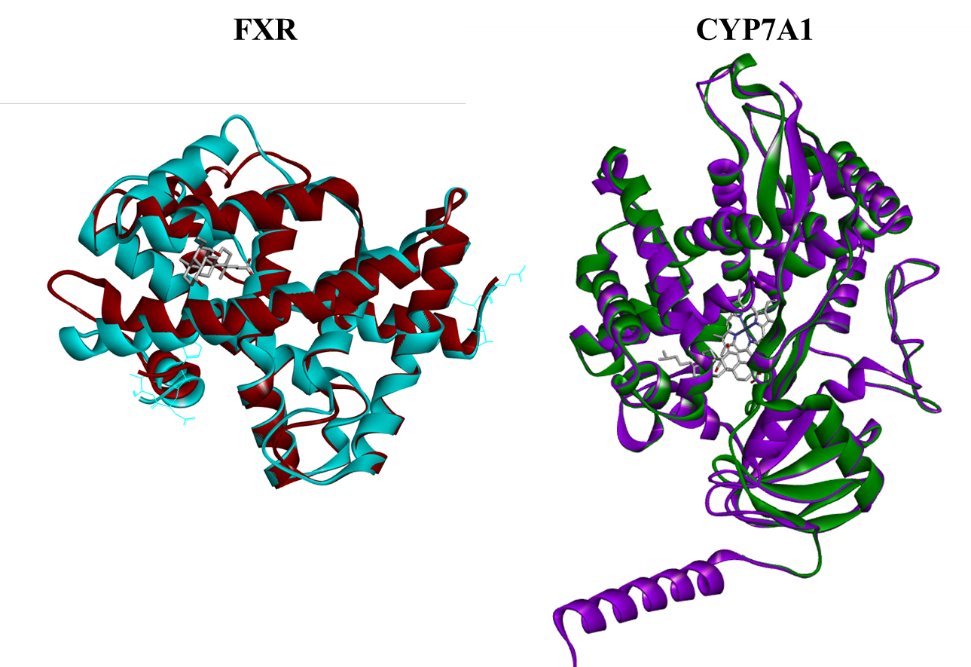** |
| --- | --- |
|  |  |
| **CYP7A1** | **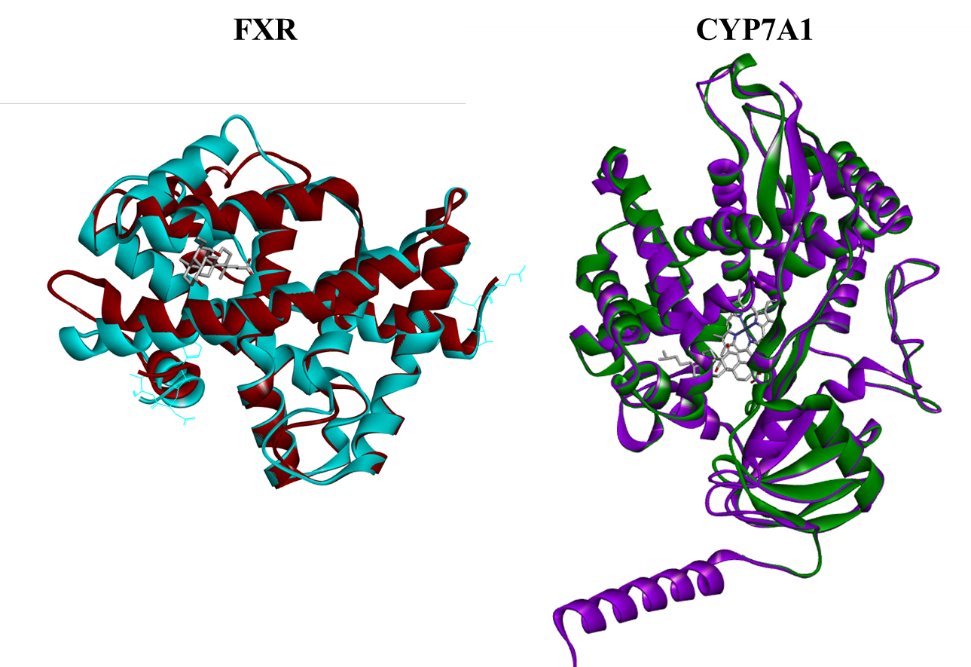** |

**Figure S3**. All the cavities detected for farnesoid X receptor (FXR) (seven cavities) and cholesterol-7-alpha-hydroxylase (CYP7A1) (twenty-three cavities) on the Cavity module of the CavityPlus server.

| **FXR** | 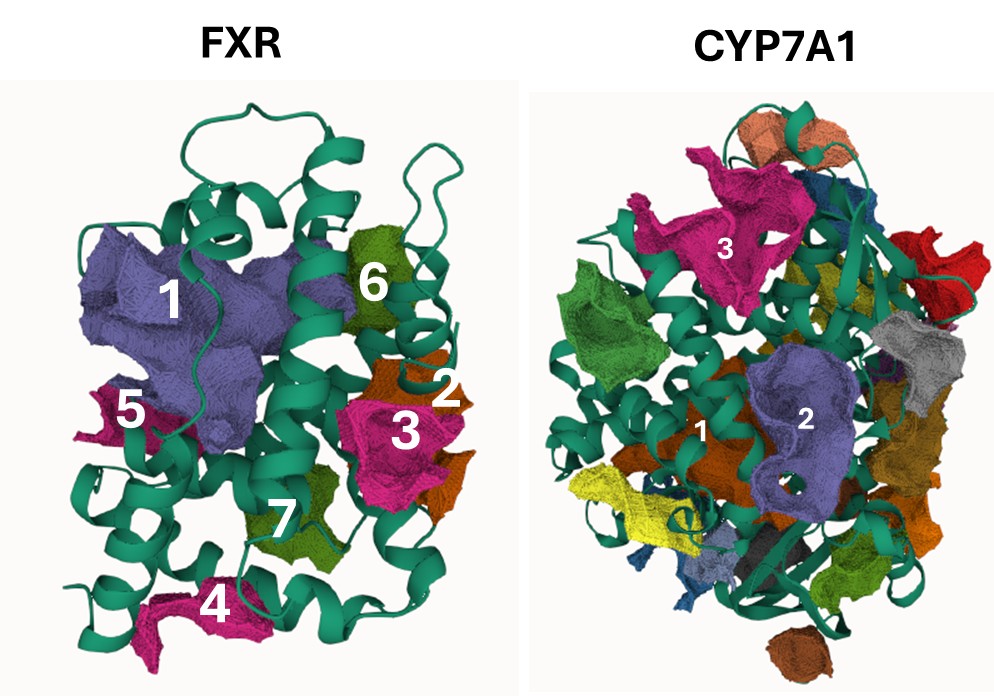 |
| --- | --- |
|  |  |
| **CYP7A1** | 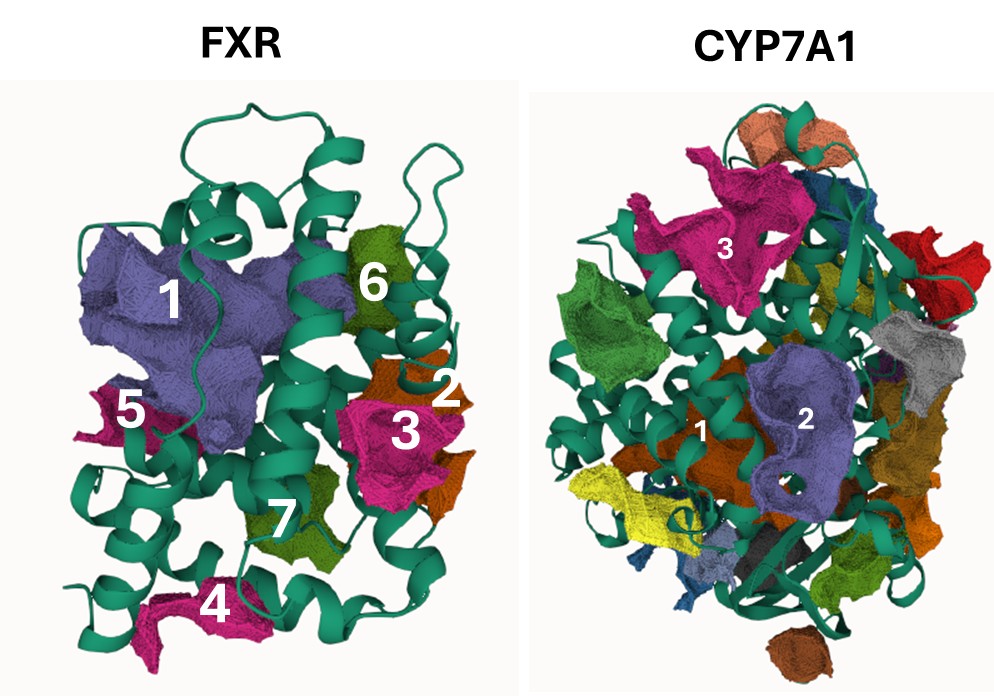 |

**Figure S4.** RMSD analysis of Cα-atoms from protein of holoenzyme (unbound) and CYP7A1-ligand complexes in the molecular dynamics simulations (200 ns). Holoenzyme (purple) (**A**); Ligand **1** (cafestol in blue) in cavity #1 (**B**), cavity #2 (**C**), and cavity #3 (**D**); Ligand **2** (15,16-dehydrocafestol in green) in cavity #1 (**E**), cavity #2 (**F**), and cavity #3 (**G**).

| 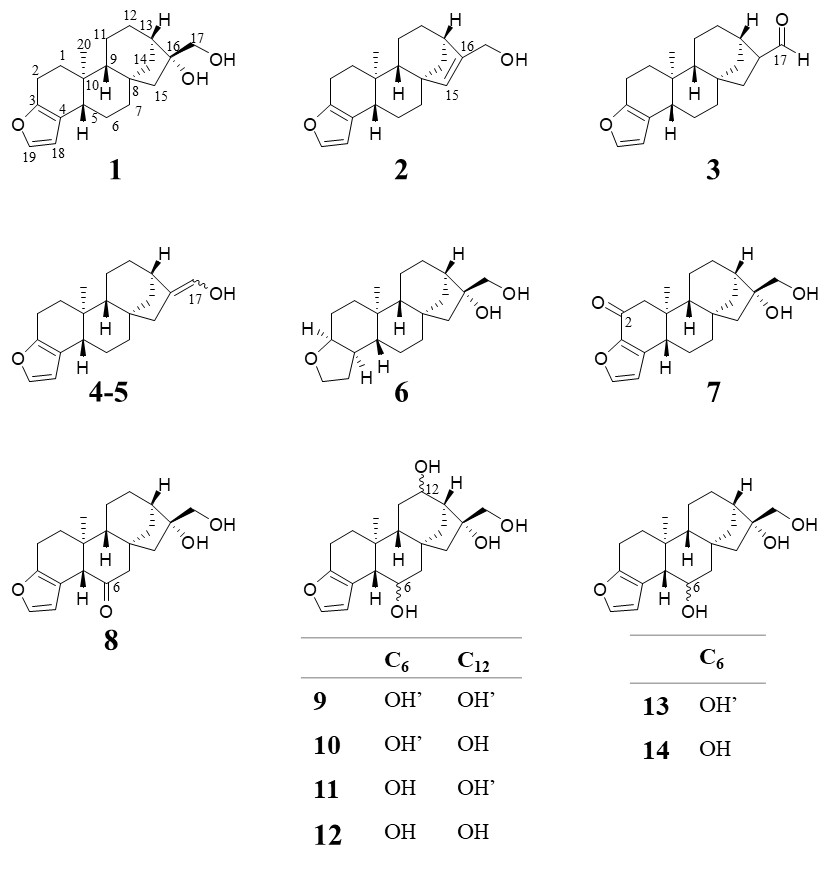 | **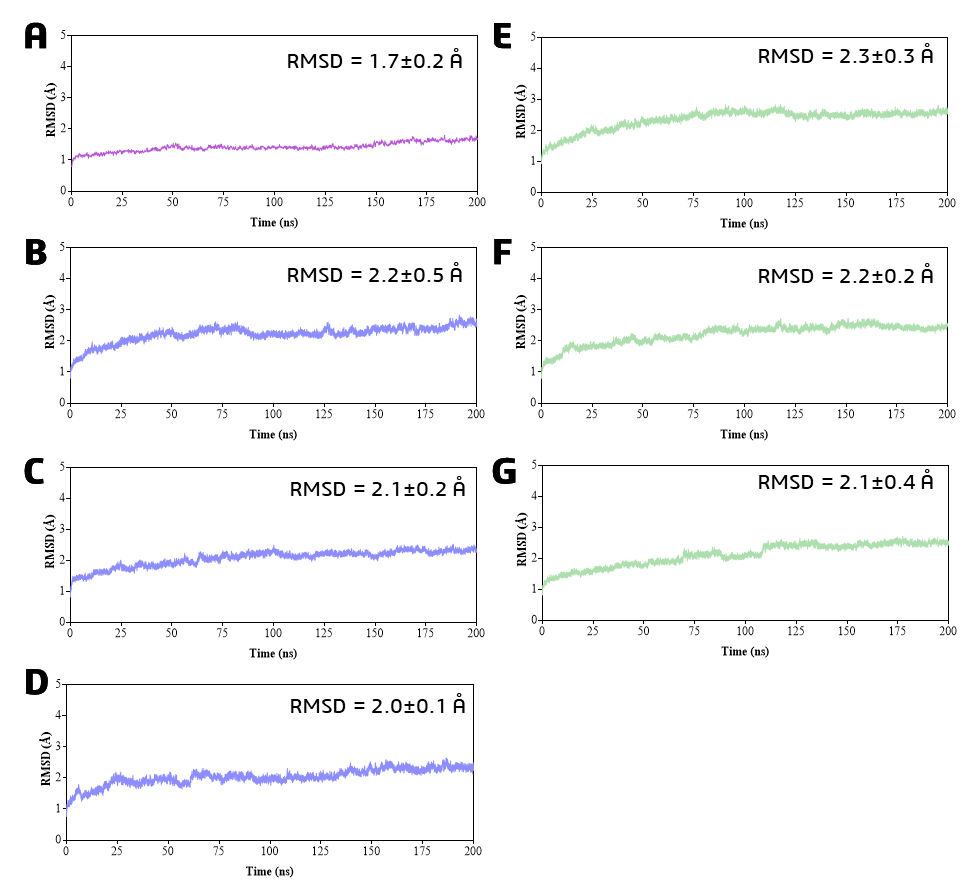** | | 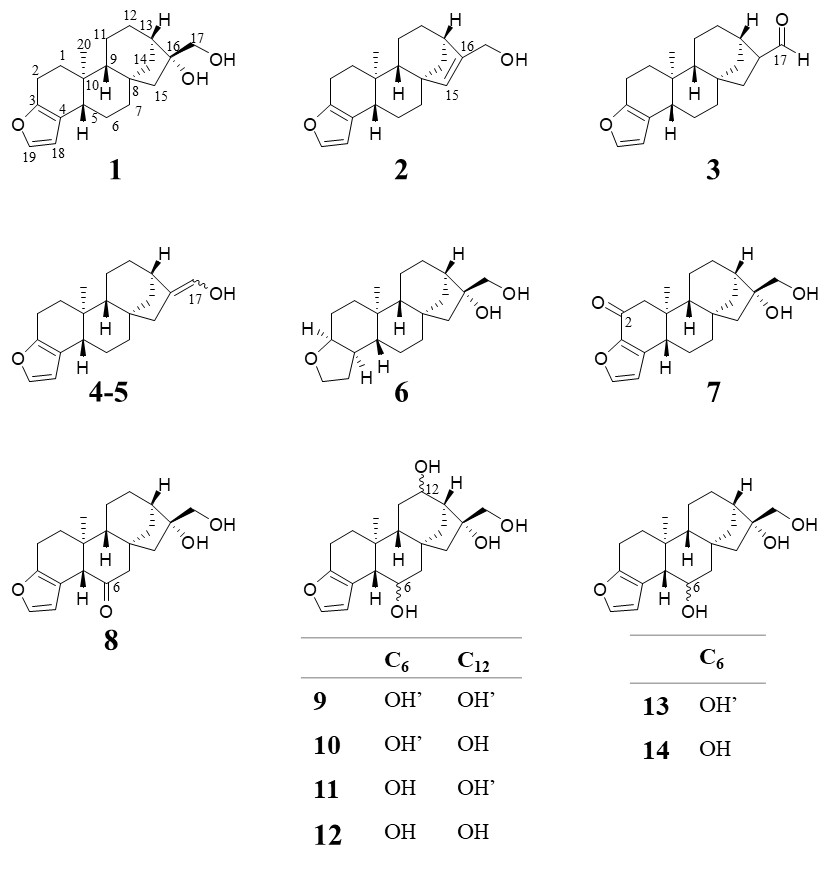 |
| --- | --- | --- | --- |
| **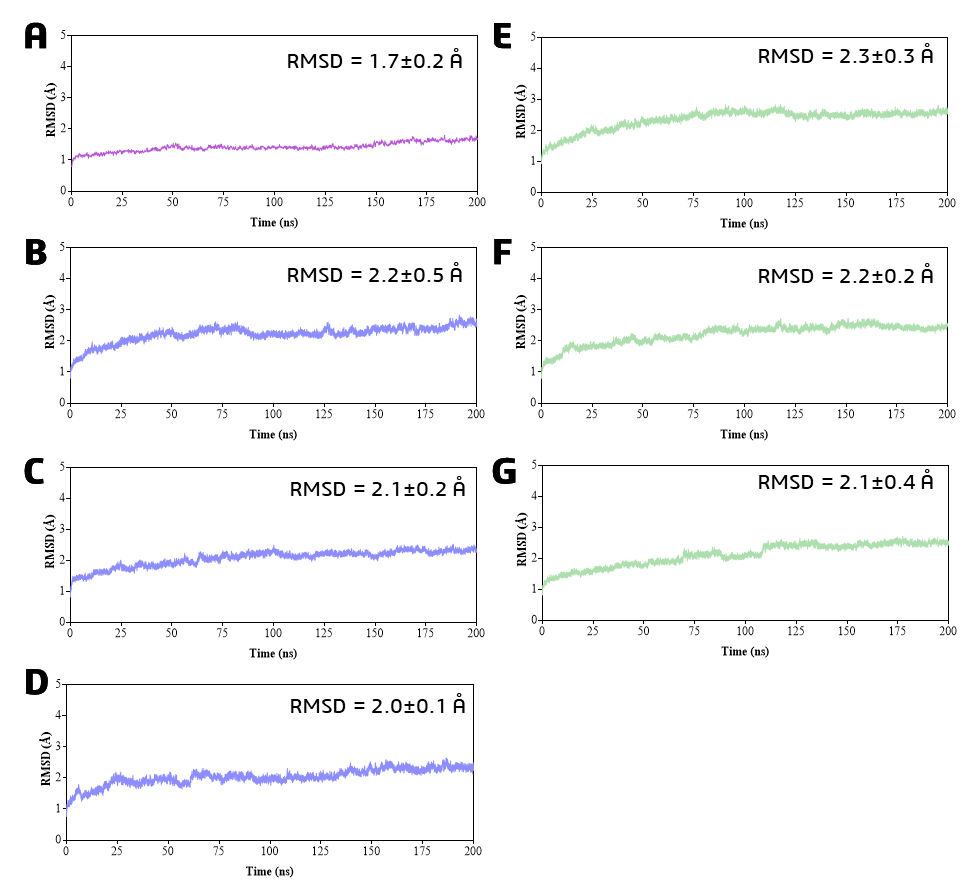** | | **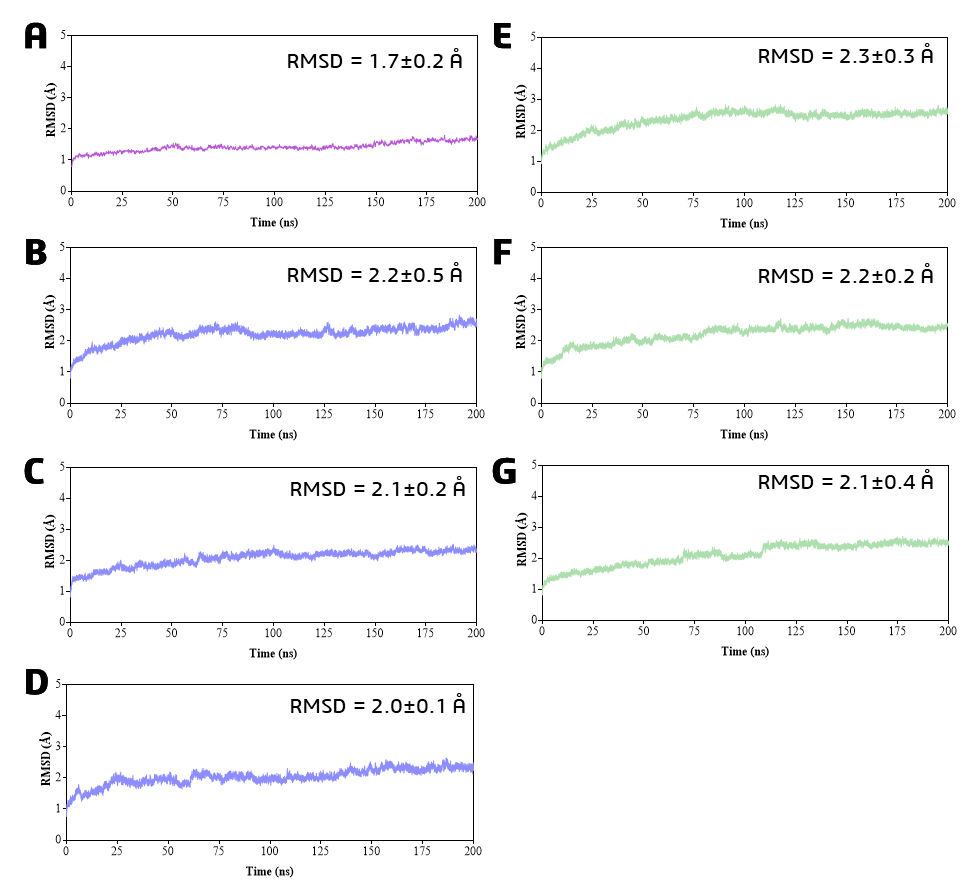** | |

**Figure S5.** RMSF analysis of Cα-atoms from the protein of holoenzyme (unbound) and CYP7A1-ligand complexes in the molecular dynamics simulations (200 ns). Holoenzyme (purple) (**A**); Ligand **1** (cafestol in blue) in cavity #1 (**B**), cavity #2 (**C**), and cavity #3 (**D**); Ligand **2** (15,16-dehydrocafestol in green) in cavity #1 (**E**), cavity #2 (**F**), and cavity #3 (**G**); (**H**) 3D-structure of CYP7A1 highlighting the residues with fluctuations ≥ 2 Å.

| 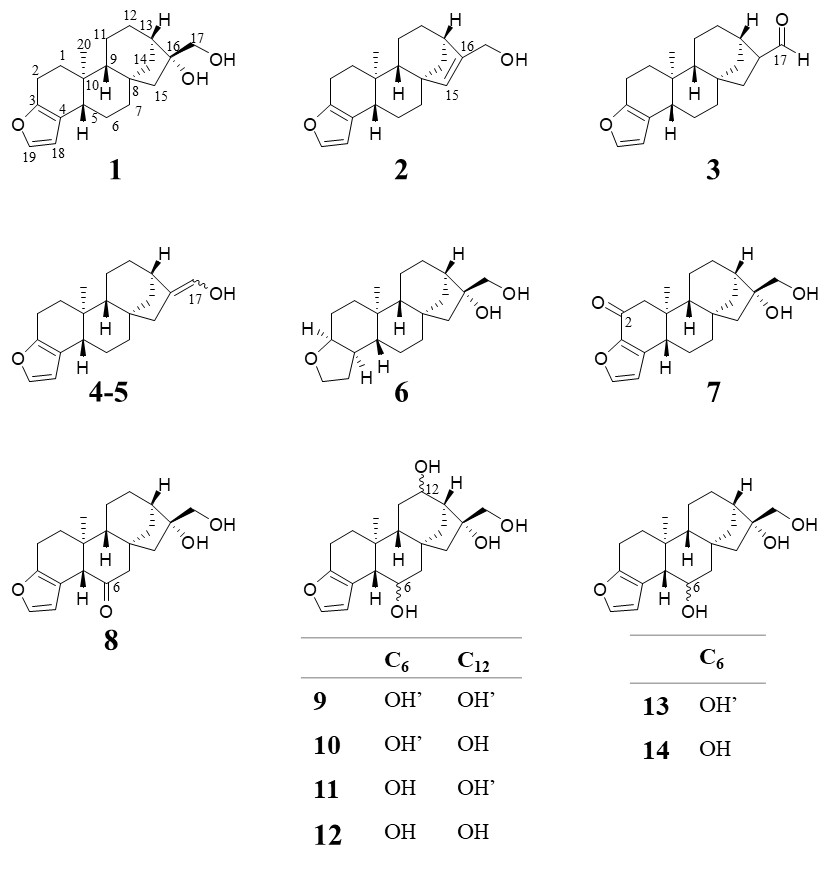 | **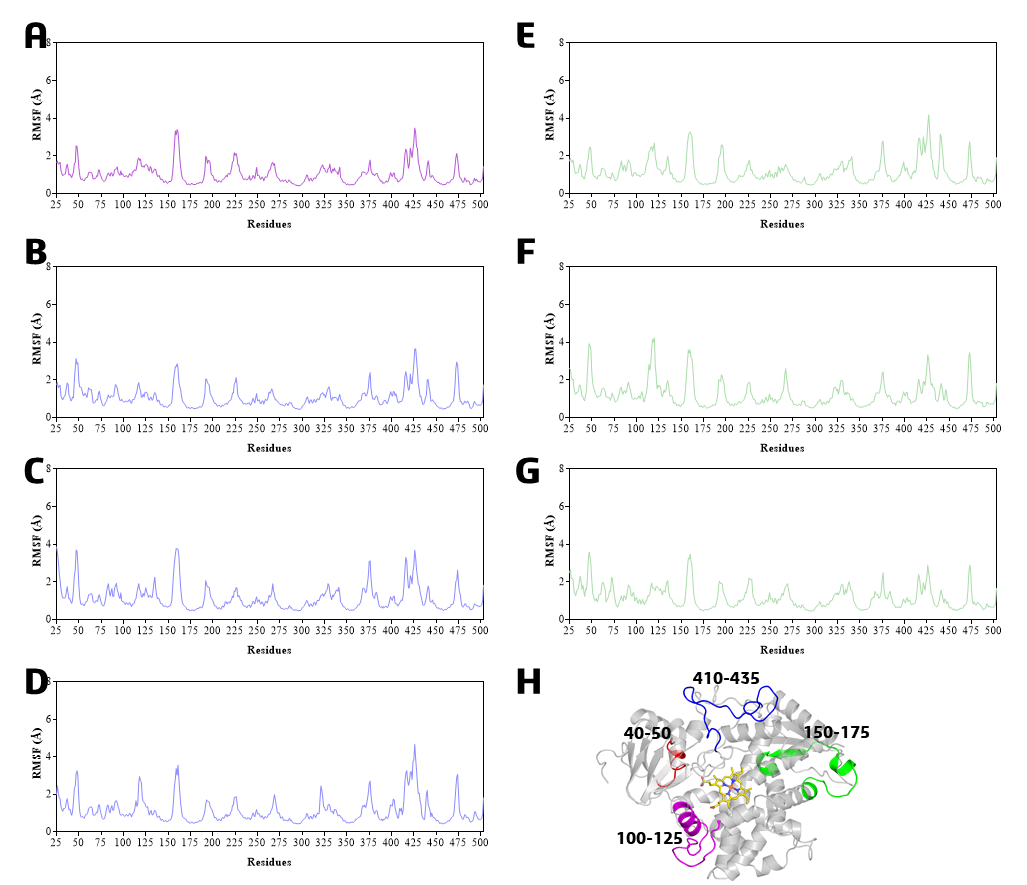** | | 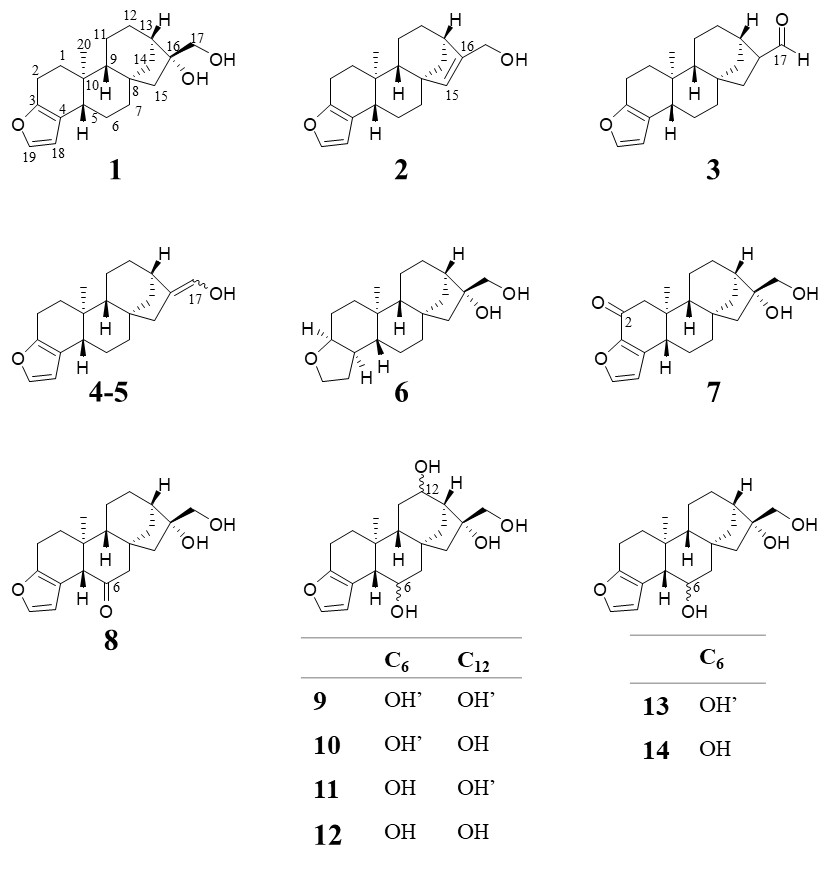 |
| --- | --- | --- | --- |
| **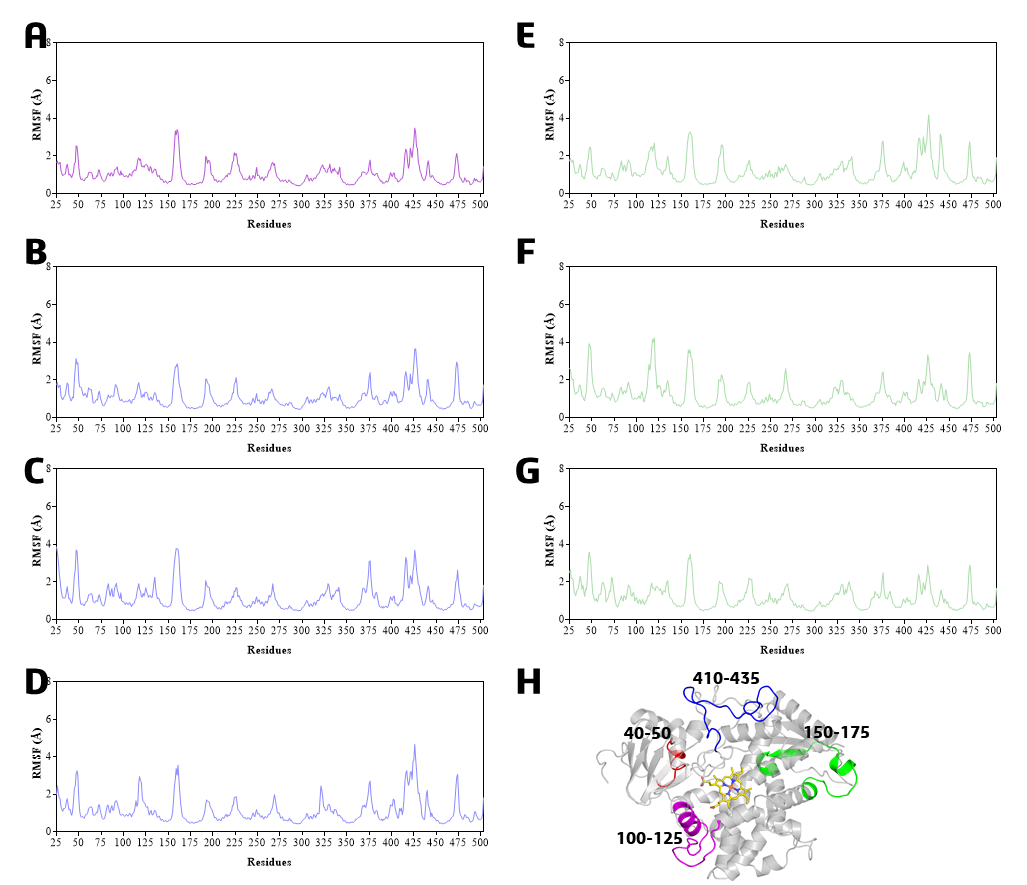** | | **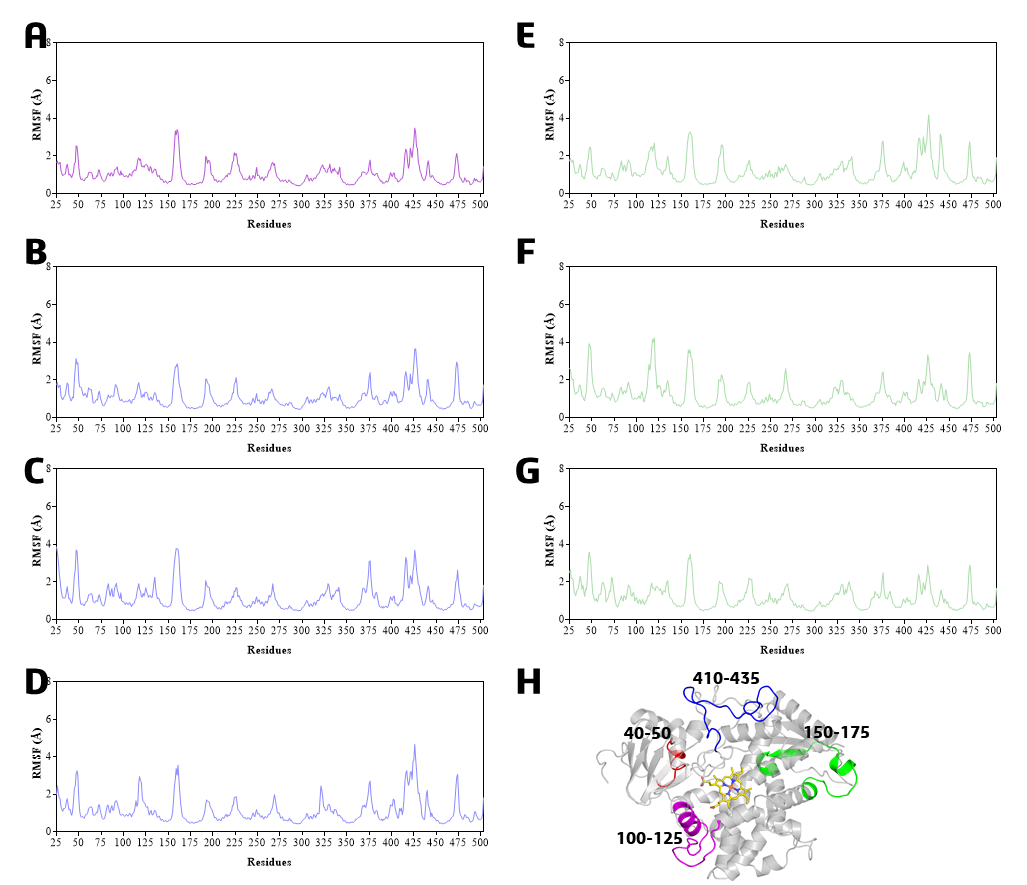** | |
| **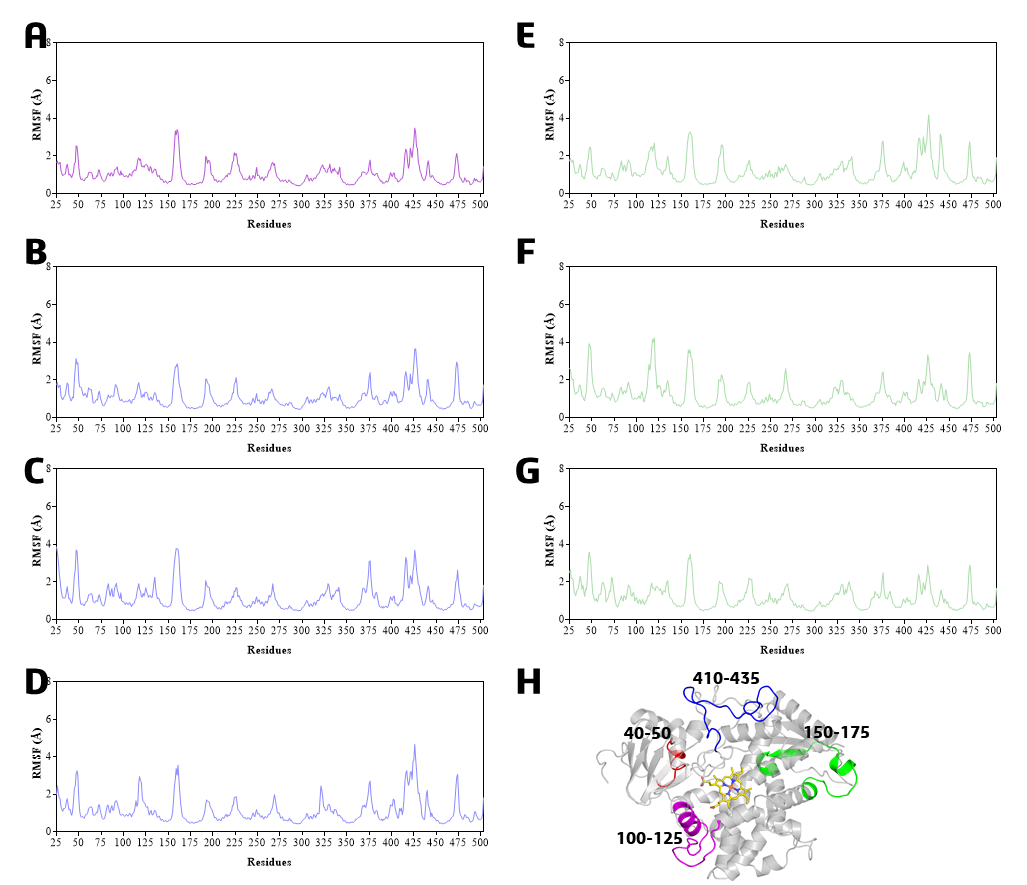** | | | |

**Figure S6.** SwissADME output panel for 15,16-dehydrocafestol (**2**).


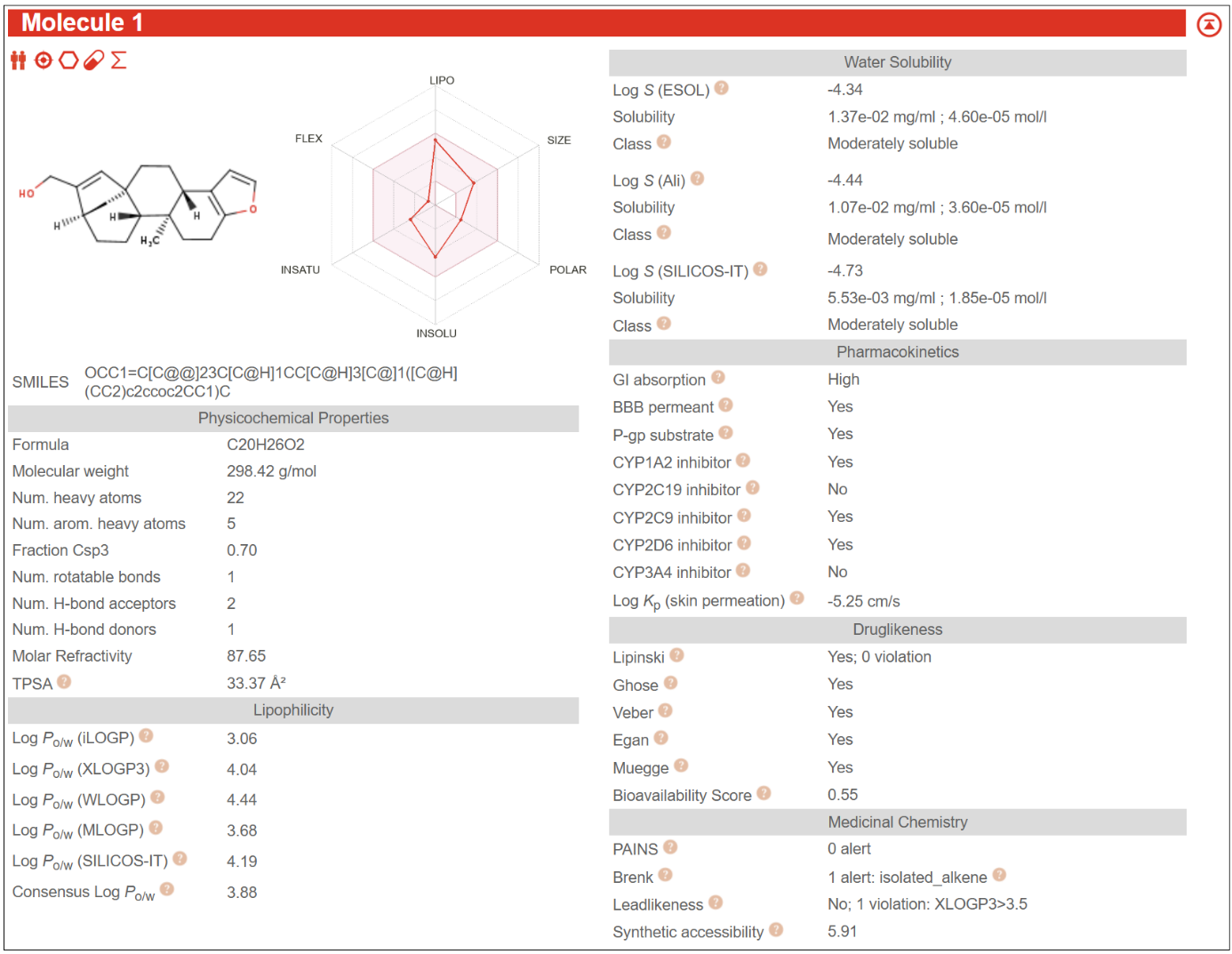


**Figure S7.** SwissADME output panel for cafestal (**3**).


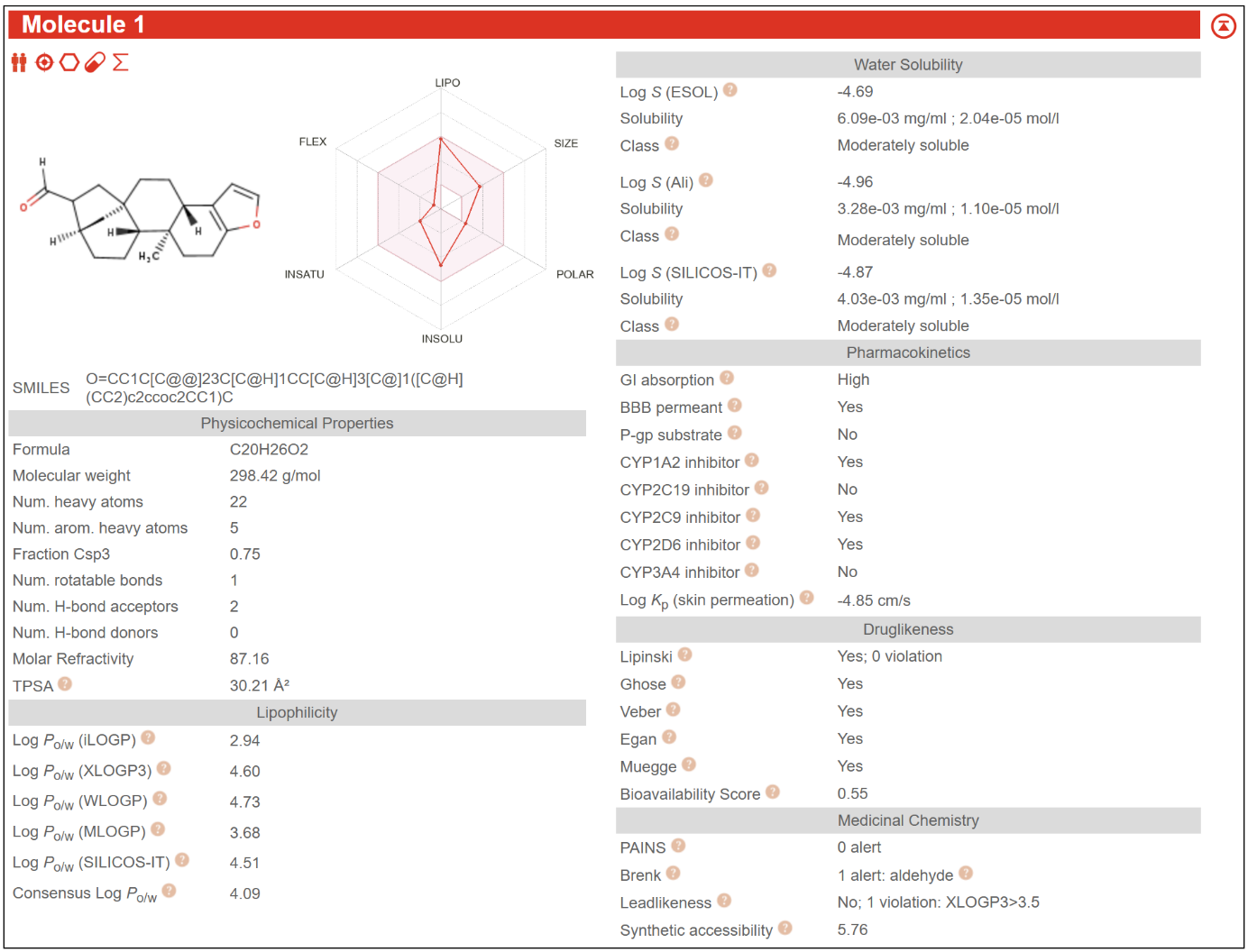


**Figure S8.** SwissADME output panel for cafestol (**1**).


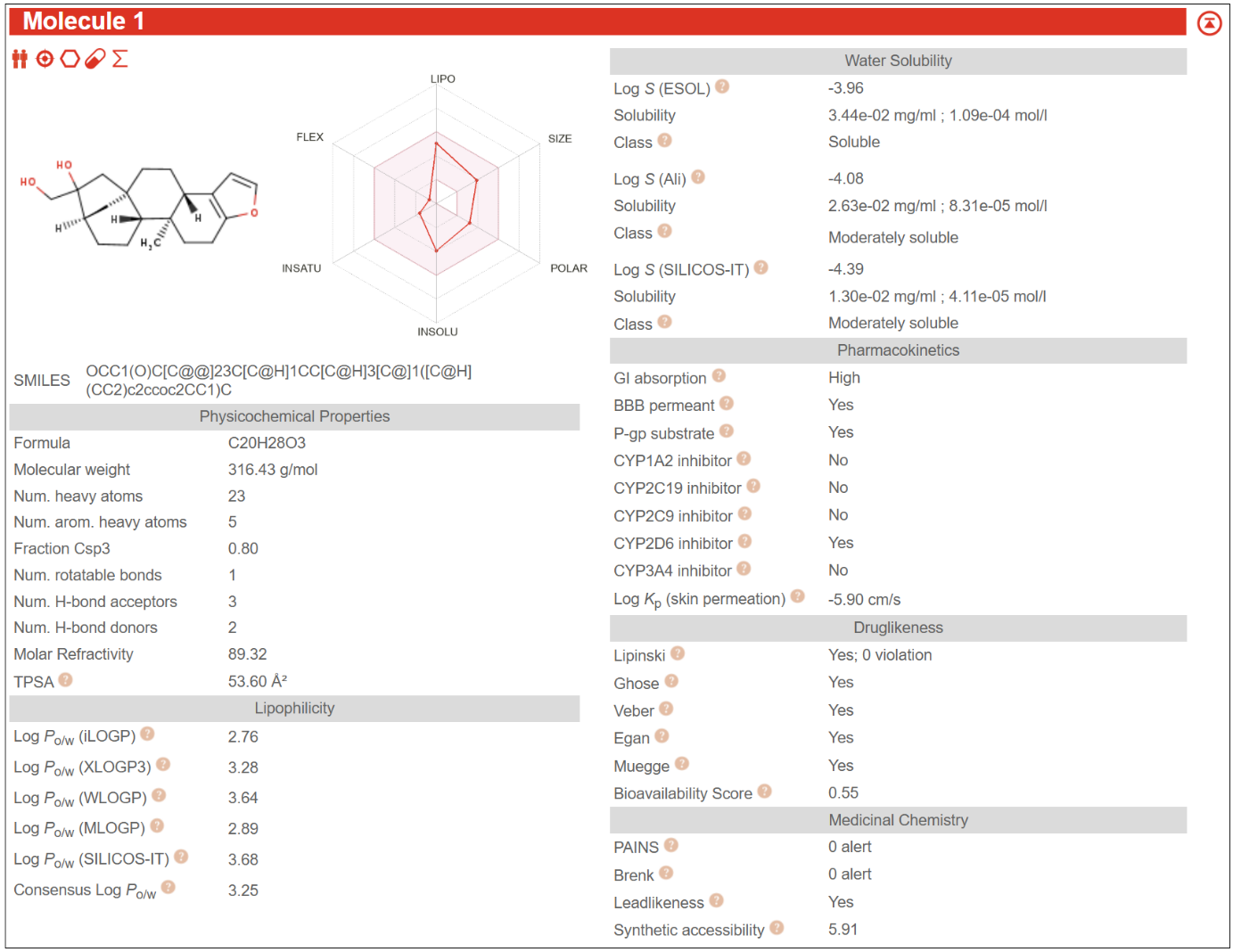

Supplement: Supplementary file 1 — Supplementary Material 1 [file 41598_2026_37519_MOESM1_ESM.docx]
